# Organogelation enables fast organolithium cross-coupling reactions in air†

**DOI:** 10.1039/d3cc00756a

**Published:** 2023-04-17

**Authors:** Paco Visser, Ben L. Feringa

**Affiliations:** a Stratingh Institute for Chemistry, University of Groningen Nijenborgh 4 9747 AG Groningen The Netherlands b.l.feringa@rug.nl

## Abstract

C–C bond formation based on palladium-catalysed cross-coupling reactions using organolithium reagents has seen major breakthroughs in the past decade. However, the use of inert conditions, as well as slow addition of the organolithium species, is generally required. Here we describe the Pd-catalysed cross-coupling of C_36_H_74_-gelated organolithium reagents with aryl bromides. The reaction proceeds in 5 min at room temperature, while eliminating the previously required slow addition, and strict use of inert atmosphere. Crucially, the use of organolithium gels facilitates handling, and offers a tremendously increased process safety, which is illustrated by a gram-scale transformation that does not require any extraordinary safety precautions.

## Abstract 2

Here we describe the Pd-catalyzed cross-coupling of organolithium gels with aryl bromides, which can be safely performed in air. The utility of the reaction is illustrated in a gram-scale reaction that does not require special safety precautions.

## Graphical abstract

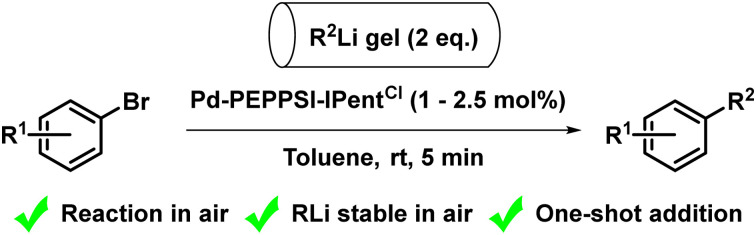

Transition metal-catalysed C–C bond forming reactions are the cornerstone of synthetic chemistry, and are a powerful tool for the efficient construction of functionalised (hetero)aromatics, which find widespread application in the synthesis of agrochemicals,^1^ pharmaceuticals,^2^ natural products,^3^ and materials.^4^ Especially Pd-catalysed cross-coupling procedures using aryl- and alkenyl (pseudo)halides,^3^ such as the Suzuki–Miyaura,^5^ Kumada–Corriu,^6^ Negishi,^7^ Stille,^8^ and Hiyama-Denmark^9^ reactions have been meticulously refined since the late 20th century.^10^ Despite their extensive development, these ‘classical’ methodologies often require the use of elevated temperatures and long reaction times, while several (organometallic) coupling partners are accessed *via* the corresponding organolithium reagent.^5,7,9^ Although seminal work on the direct cross-coupling of organolithium reagents was reported,^11^ further developments stagnated for over 25 years, mainly resulting from a lack of selectivity using these highly reactive compounds.^12^ Building on our previous experience with asymmetric allylic substitutions using alkyllithium compounds,^13^ our group tamed the formidable reactivity of organolithium reagents for direct cross-coupling reactions in 2013. This resulted in efficient Pd-catalysed C–C bond formations with organohalides in fast reactions under mild conditions.^12,14^ Since then, Murahashi–Feringa cross-coupling reactions using a plethora of organolithium reagents and transition-metals have been reported.^12^ Crucially however, the use of inert conditions, and slow addition of the organolithium species, circumventing catalyst poisoning, is generally required.^12,15^ Towards solving the issue of slow addition, Capriati and co-workers have demonstrated that C–C bond formation is possible ‘on-water’, enabling rapid organolithium addition in air (Scheme 1a).^16^ The adaptation of such procedures on larger scale would however require caution due to the severe moisture-sensitivity of organolithium solutions.^15^ Furthermore, in collaboration with the Organ group, we reported that one-shot addition is possible using the Pd-PEPPSI-IPent^Cl^ catalyst, which displays near-diffusion limited reactivity, although requiring strictly inert conditions (Scheme 1b).^17^ In an effort to stabilize organolithium reagents in air, Smith and co-workers recently demonstrated the use of C_36_H_74_ as a low-molecular-weight organogelator (LMWG) to form organolithium gels, which were successfully employed in *e.g.* 1,2-addition- and Li–Br exchange reactions.^18^ LMWGs are known to self-assemble into fibre networks at low gelator concentrations,^19^ and were therefore a suitable candidate to encapsulate *n*BuLi and PhLi in a gel matrix, rendering them significantly less moisture-sensitive. Importantly, these C_36_H_74_-organolithium gels were used in air without requiring any extraordinary safety precautions. As such, their further development might provide also increased safety in the transmetalation of organolithium- to *e.g.* organoboron reagents,^5^ anionic polymerization reactions,^20^ and anion relay chemistry.^21^ Building on the previous experience of our group on the use of organogelators,^19*b*,22^ we describe herein the cross-coupling of C_36_H_74_-gelated alkyl- and phenyllithium reagents with aryl bromides (Scheme 1c). The reaction proceeds in air, requiring only 1–2.5 mol% of Pd-PEPPSI-IPent^Cl^ catalyst, with a reaction time of 5 min, and eliminates the tremendous safety risks associated with handling highly reactive organolithium reagents in air.

**Scheme 1:**
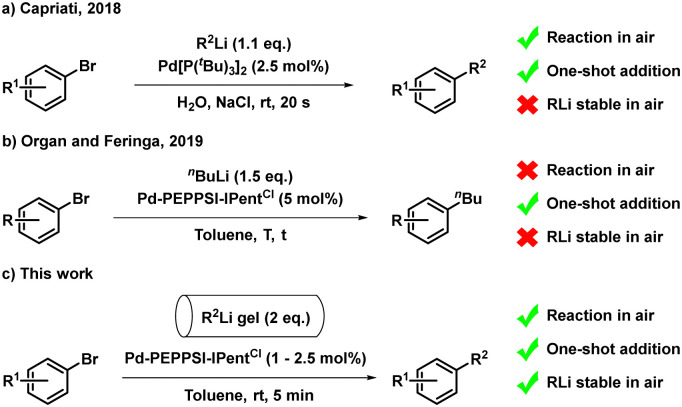
State of the art overview of fast organolithium cross-coupling reactions. (a) Fast cross-couplings 'on-water'. (b) Fast cross-couplings using Pd-PEPPSI-IPent^Cl^. (c) This work.

We initiated our investigations with the reaction between 4-bromoanisole (1) and C_36_H_74_-gelated *n*BuLi, which was exposed to air at rt for 5 min (2.7 eq.) (Table 1). Importantly, performing the reaction in the absence of a transition metal-catalyst for 5 min afforded only 1% of the desired product 2, along with 35% of the dehalogenation product 4 as a result of Li–Br exchange (Entry 1). The cross-coupling reaction (10 min reaction time) in the presence of either Pd-PEPPSI-IPr^23^ (Entry 2) or Pd-PEPPSI-IPent^24^ (Entry 3) (5 mol%) provided 2 in 75% and 73%, respectively. The use of XPhos Pd G4^25^ (Entry 4, 23%), or [Pd(μ-I)P(*t*Bu)_3_]_2_^26^ (Entry 5, 37%) resulted in diminished conversions. Performing the reaction using 5 mol% Pd[P(*t*Bu)_3_]_2_, which was previously successfully employed in our organolithium cross-coupling reactions,^14^ afforded 97% of 2 (Entry 6). Identical selectivity was retained using 0.5 mol% catalyst (Entry 7; see ESI,† Table S1 for full optimization data). Decreasing the reaction time to 5 min reduced the conversion into 2 to 84% (Entry 8). Oxygen-activated Pd[P(*t*Bu)_3_]_2_^27^ was also found to be an efficient catalyst in the transformation, forming 2 in 96% with only 1.25 mol% catalyst loading (Entries 9 and 10). Lowering the catalyst loading to 0.5 mol% slightly decreased the selectivity towards 2 (90%, Entry 11). Using 1.25 mol% catalyst, the reaction time could be reduced to 1 min, forming 2 in 96% (Entry 12). Further decreasing the catalyst loading to 1 mol% afforded 2 in 92% (Entry 13). Gratifyingly, switching the catalyst to Pd-PEPPSI-IPent^Cl^ (5 mol%), which was previously shown to enable near-diffusion limited reactivity in organolithium cross-couplings,^17^ afforded 2 as the sole product with quantitative conversion (Entry 14). The catalyst loading could be reduced to 1 mol% in conjunction with a reaction time of 1 min, resulting in near-identical results (Entry 15). Importantly, performing the reaction without C_36_H_74_-gelation afforded 2 in only 14% (Entry 16). Further lowering the catalyst loading to 0.5 mol% resulted in diminished selectivity (Entry 17). The nearly perfect reaction conditions were found following optimization of the *n*BuLi gel characteristics (ESI,† Table S2), using 1 mol% Pd-PEPPSI-IPent^Cl^, 5 min reaction time, 2 eq. *n*BuLi (lowering the *n*BuLi excess resulted in diminished conversion, and selectivity into 2), and crucially exposing the gel to air at 0 °C, instead of rt, in order to avoid degelation (Entry 18). Slight thermally-induced degelation was only observed when rt was at least 25 °C, which could be efficiently circumvented by the aforementioned pre-equilibration protocol at 0 °C.

**Table 1:** Reaction optimization

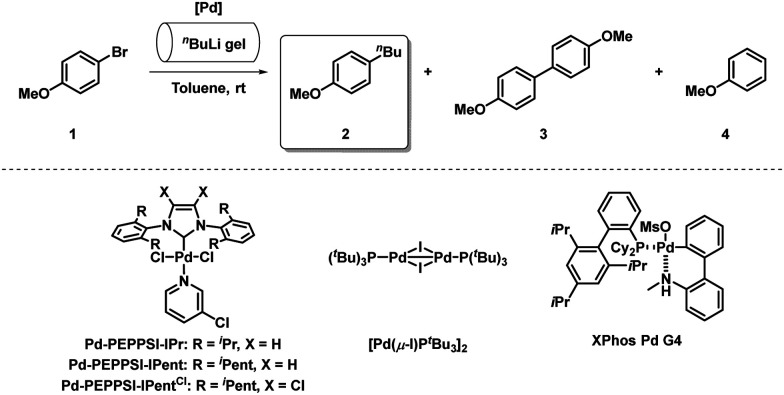				
Entry^a^	[Pd]	[Pd] (mol%)	Time (min)	Conv. (%) 2 : 3:4^b^
1	—	—	5	1 : 0 : 35
2	Pd-PEPPSI-IPr	5	10	75 : 0 : 14
3	Pd-PEPPSI-IPent			73 : 0 : 27
4	XPhos Pd G4			23 : 3 : 40
5	[Pd(*μ*-I)P(*t*Bu)_3_]_2_	2.5		37 : 2 : 22
6	Pd[P(*t*Bu)_3_]_2_	5		97 : 0 : 3
7		0.5		97 : 0 : 3
8			5	84 : 6 : 5
9	Pd[P(*t*Bu)_3_]_2_/O_2_^c^	5	10	96 : 4 : 0
10		1.25		96 : 1 : 3
11		0.5		90 : 0 : 8
12		1.25	1	96 : 0 : 4
13		1		92 : 1 : 6
14	Pd-PEPPSI-IPent^Cl^	5	10	>99 : 0 : 0
15		1	1	99 : 0 : 1
16^d^				14 : 0 : 18
17		0.5	10	90 : 0 : 8
**18** e		**1**	**5**	**99 : 0 : 1**

a Reaction conditions: Catalyst and 1 (0.3 mmol) in toluene (2 mL) was added to *n*BuLi gel (5 min pre-exposure to air at rt; *n*BuLi (2.7 eq.; 1.6 M in hexanes), 0.5 mL hexanes, 4% wt/vol C_36_H_74_). The mixture was vigorously stirred, and quenched by addition of H_2_O after the specified time.

b Determined by gas chromatography-mass spectrometry analysis.

c Formed by purging a solution of Pd[P(*t*Bu)_3_]_2_ in toluene with O_2_.

d No gelation.

e Gel formed with 2 eq. *n*BuLi (1.6 M in hexanes), 0.5 mL hexanes, 4% wt/vol C_36_H_74_, 4 min pre-exposure to air at 0 °C, 1 min at rt. Linear upscaling to 0.5 mmol 1 afforded identical results. See also ESI, Table S2.

We next proceeded to investigate the generality of the transformation (Table 2). Although the conversion and selectivity were usually excellent, chromatographic purification occasionally affected the isolated yield. For instance, anisole 5 could be isolated in 66% yield, while the isolation of 6 and 7 was troublesome, likely resulting from van der Waals (VdW) interactions of the highly apolar products with C_36_H_74_.^28^*N*,*N*-dimethylaniline 8 (76%) and sterically encumbered cumene 9 (73%) could be isolated in satisfactory yields, presumably by disruption of the VdW interactions.

**Table 2:** Generality of the transformation using different aryl bromides

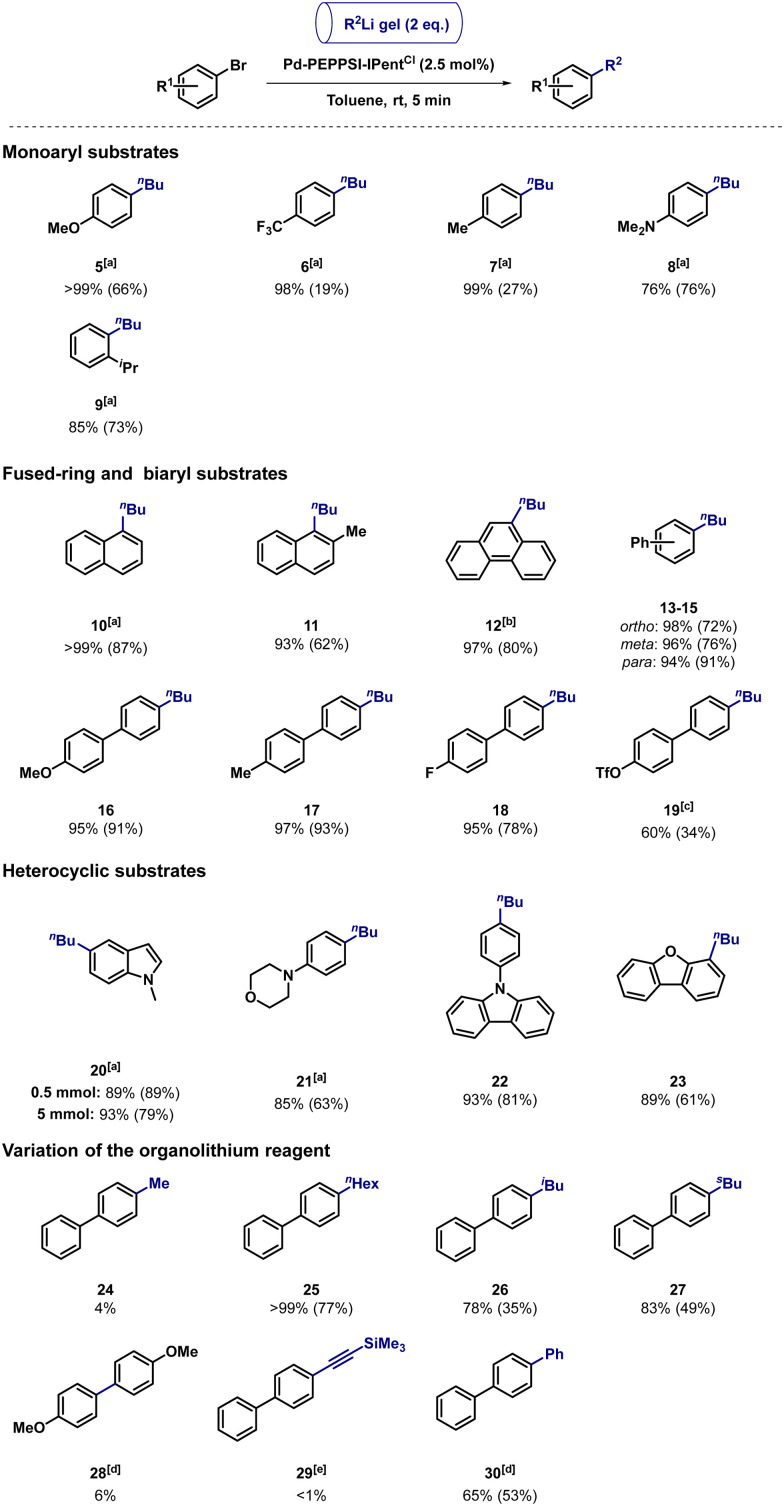

a 1 mol% Pd-PEPPSI-IPent^Cl^.

b Yield corrected for the presence of 7% *s*Bu-isomer.

c 2.5 mol% Pd[P(*t*Bu)_3_]_2_/O_2_ as catalyst.

d 5 mol% Pd-PEPPSI-IPent^Cl^.

e 5 mol% Pd[P(*t*Bu)_3_]_2_/O_2_ as catalyst.

Naphthalenes 10 and 11 were isolated in 87% and 62% yield, respectively. Phenanthrene 12 (80% corrected yield) was isolated as an inseparable mixture with its *s*Bu-isomer,^29^ formed by the competing pathway of *β*-H elimination followed by migratory reinsertion.^30^ In general, the coupling of fused-ring- and biaryl bromides required 2.5 mol% catalyst to suppress the formation of chromatographically inseparable side-products (generally 5–30%) resulting from the competing Li–Br exchange reaction.^12^*Ortho*-, *meta*-, and *para*- (13–15) as well as OMe- (16, 91%) and Me-substituted (17, 93%) biphenyls were isolated in excellent yields. The chemoselectivity of the transformation was demonstrated in the synthesis of fluoride- and triflate-functionalized products 18 and 19, bearing handles for further diversification. In the case of 19, the use of Pd[P(*t*Bu)_3_]_2_/O_2_ (2.5 mol%) as the catalyst was required to achieve high chemoselectivity. The corresponding chloride-substituted product could not be separated from the undesired bis-butylated side-product (see ESI†). We next investigated the cross-coupling of heterocyclic compounds, due to their prevalence as a structural feature in *e.g.* pharmaceuticals.^2^ To our delight, products based on *N*-methylindole- (20), morpholine- (21), carbazole- (22) and dibenzofuran (23) could be isolated in up to 89% yield. Substrates that were incompatible with the reaction conditions, bearing *e.g.* a nitrile or nitro functionality, and 2-bromopyridine, are discussed in the ESI.† Additionally, we aimed to perform the reaction on gram-scale, illustrating the scalability of the protocol. Crucially, we targeted the use of *n*BuLi block gels, formed using 16.7 wt/vol% C_36_H_74_, which are stable under N_2_ for at least one week and can be added to the reaction in air.^18^ The use of such air-stable gel blocks would tremendously decrease the required safety protocols associated with performing organolithium chemistry on preparative scale.^15^ Satisfyingly, *N*-methylindole 20 was efficiently synthesized in 79% isolated yield on 1.05 g (5 mmol) scale. While a large amount of C_36_H_74_ (2.8 g) is required for such transformations, we anticipate that the gelator can be efficiently separated, and recycled by *e.g.* distillation when adapting the protocol to multi-gram scale chemistry. Crucially, C_36_H_74_-gelation enabled us to safely use >5 mL of *n*BuLi solution in air without requiring any extraordinary safety precautions. Although the handling of the organolithium reagent under inert atmosphere was still required during the gel preparation stage, complete circumvention would be accomplished if organolithium gels become commercialized, enabling the direct use of the stabilized reagents. We next investigated the incorporation of different organolithium reagents into the gel matrix, and tested the compatibility of the corresponding gels with the protocol. The use of MeLi afforded only 4% of 24 (96% dehalogenation). This is expected to either arise from i) the lower aggregation state (tetrameric) of MeLi solution in comparison to *n*BuLi solution (hexameric), resulting in a higher reactivity,^31^ or ii) the poor solubility of MeLi in Et_2_O/hexane mixtures, resulting in precipitation, and a potentially diminished reactivity. Based on previous experiences, aggregation states play a key role in the direct cross-coupling of organolithium reagents.^12,14^ Reactions using *n*HexLi (25; 77%), *i*BuLi (26; 35%) and *s*BuLi (27; 49%) afforded the desired products in moderate to good yields, with the diminished isolated yields of 26 and 27 resulting from troublesome chromatographic separation. Underlining the potential applicability of these gels, extremely moisture-sensitive *t*BuLi solution was gelated, although affording mainly dehalogenation (39%) in the homocoupling of 4-bromoanisole (28, 6%, 45% substrate conversion). This was also the case in the Pd[P(*t*Bu)_3_]_2_/O_2_-catalysed reaction using lithium (trimethylsilyl)acetylide gel (29).^32^ It is important to note that in this example we observed the same solubility issues as when using MeLi. Finally, in addition to the use of alkyllithium gels, the C(sp)^2^-C(sp)^2^ cross-coupling of PhLi gel afforded 30 in 53% yield using 5 mol% Pd-PEPPSI-IPent^Cl^.

Having established a wide scope and in order to demonstrate the synthetic applicability of the transformation we selected the synthesis of thianaphthene 32, an intermediate towards perfluorocyclopentene-based photoswitch 33, which shows photochromic properties in hexane (Scheme 2).^33^

**Scheme 2:**
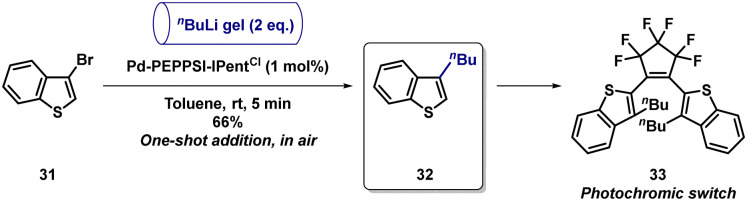
Synthetic applicability of the protocol.

Under our reaction conditions, 32 was obtained in 66% yield using 1 mol% Pd-PEPPSI-IPent^Cl^ in 5 min. In previous work by our group, the desired product was obtained in a higher yield (84%).^27^ In that case however, a higher catalyst loading (5 mol%), as well as the use of strictly inert conditions and slow addition of *n*BuLi were required, emphasizing the simplicity of our developed protocol.

In conclusion, we have demonstrated the cross-coupling of alkyl- and phenyllithium gels with aryl bromides in air. The reaction proceeds rapidly (5 min) using down to 1 mol% Pd-PEPPSI-IPent^Cl^. A variety of fused-ring, biaryl, and heterocyclic products were readily synthesized. Furthermore, an array of organolithium reagents were gelated, showcasing their stability and general applicability. Moreover, the reaction was performed on gram-scale (>5 mL *n*BuLi solution) without requiring any extraordinary safety precautions. The gelation of organometallic reagents therefore opens attractive opportunities towards applicability on larger scales, greatly increasing process safety.

B.L.F. acknowledges financial support from the Netherlands Organisation for Scientific Research, the European Research Council (ERC Advanced Grant 694345), the Royal Netherlands Academy of Arts and Sciences (KNAW), and the Ministry of Education, Culture and Science (Gravitation program 024.001.035). R. Sneep is acknowledged for performing the high-resolution mass spectrometry.

## Conflicts of interest

There are no conflicts to declare.

## Supplementary Material

CC-059-D3CC00756A-s001
