# Supplementary material for: Organogelation enables fast organolithium cross-coupling reactions in air
Source: Chem Commun (Camb). 2023 Apr 17;59(37):5539–42. doi: 10.1039/d3cc00756a (PMC10157804; doi:10.1039/d3cc00756a)
Supplement: CC-059-D3CC00756A-s001 [file CC-059-D3CC00756A-s001.pdf]

Electronic Supplementary Material (ESI) for ChemComm.
This journal is © The Royal Society of Chemistry 2023

Supporting information

Organogelation enables fast organolithium cross-coupling reactions in air

Paco Visser, Ben L. Feringa*

Stratingh Institute for Chemistry, University of Groningen, Nijenborgh 4, 9747 AG Groningen, The Netherlands.

Contents

1. **General methods** 3
2. **General procedures** 4
3. **Table S1 - Full data overview for the catalyst screening** 5
4. **Table S2 – Full data overview for the optimization of the ^{n}BuLi gel characteristics** 6
5. **Formation and stability of organolithium gels** 6
6. **Substrates incompatible with the reaction conditions** 8
7. **Synthesis of products** 9
8. **Supplementary References** 15
9. **Spectral data** 16

1. General methods

Column chromatography: Biotage® Selekt System with Biotage® Sfär Silica HC (25 g) cartridges. TLC: Merck silica gel 60, 0.25 mm. Components were visualized by UV (254 nm, 365 nm) and potassium permanganate or cerium ammonium molybdate staining. Progress of the reaction and conversion were determined by GC-MS (GC, HP6890: MS HP5973) with an HP1 or HP5 column (Agilent Technologies, Palo Alto, CA). Mass spectra were recorded on an AEI-MS-902 mass spectrometer (EI+) or a LTQ Orbitrap XL (ESI+, ESI-, APCI+). ^{1}H-, ^{13}C- and ^{19}F NMR were recorded on a Varian AMX400 (400, 100.59 and 376.38 MHz, respectively) or 300 MHz (300, 75 MHz, respectively for ^{1}H- and ^{13}C NMR) using $CDCl_3$ as solvent. Chemical shift values are reported in ppm with the solvent resonance as the internal standard ($CHCl_3$: δ 7.26 for ^{1}H, δ 77.16 for ^{13}C). Data are reported as follows: chemical shifts, multiplicity (s = singlet, d = doublet, t = triplet, q = quartet, p = pentet, m = multiplet), coupling constants (Hz), and integration. All reactions were carried out under an inert atmosphere using dried glassware and using standard Schlenk techniques unless noted otherwise. THF, toluene, and hexane were dried by a MRBAUN solvent purification system (SPS). All purchased chemicals were directly used without further purification unless stated otherwise. $[Pd(\mu\text{-}I)P(^{t}Bu)_3]_2$,[1] Pd-PEPPSI-IPent^{Cl},[2] and $Pd[P(^{t}Bu)_3]_2/O_2$[3] were synthesized according to reported procedures. The formation of the organolithium gels was adopted from the report by Smith and co-workers.[4]

2. General procedures

General procedure A (GP-A) for the cross-coupling of organolithium gels (0.5 mmol scale)

A 20 mL crimp-top vial was charged with hexatriacontane (66 mg; 4 wt/vol%) and a stirring egg, and was sealed. The vial was evacuated and back-filled with nitrogen thrice. Dry ^{n}Hexane and commercially available organolithium solution (1 mmol; 2 eq.) were then added consecutively (1.64 mL total volume). The suspension was gently heated with a heat-gun until the gelator dissolved, after which the vial was cooled at 0 °C (ice bath) for 1 min to form the organogel. The vial was opened to air at 0 °C for 4 min, and rt for 1 min. A mixture of the substrate (0.5 mmol) and Pd-PEPPSI-IPent^{Cl} (10.8 mg; 2.5 mol%) in toluene (3.4 mL) was then added on top of the gel, and the mixture was vigorously stirred for 5 min at rt, after which the reaction was quenched by addition of MeOH (0.5 mL). A mixture of MeCN and THF (1/1; 10 mL) was added, and the suspension was filtered over a celite plug, which was washed with MeCN/THF (3 x 10 mL). The filtrate was then concentrated onto silica, after which the desired product was obtained by automated flash column chromatography.

General procedure B (GP-B) for the cross-coupling of ^{n}BuLi block gels (5 mmol scale)

A 50 mL crimp-top vial was charged with hexatriacontane (2.76 g; 16.7 wt/vol%) and a stirring egg, and was sealed. The vial was evacuated and back-filled with nitrogen thrice. Dry ^{n}Hexane (10 mL) and commercially available ^{n}BuLi solution (6.4 mL; 10 mmol; 2 eq.) were then added consecutively. The suspension was gently heated with a heat-gun until the gelator dissolved, after which the hot solution was taken up into a 24 mL syringe, which was cooled at 0 °C (ice bath) for 1 min to form the organogel. The syringe was carefully cut open, and the gel was equilibrated to rt for 1 min. It was then added to a vigorously stirred mixture of the substrate (5 mmol) and Pd-PEPPSI-IPent^{Cl} (108 mg; 2.5 mol%) in toluene (34 mL). After the vigorous stirring was continued for 5 min at rt, the reaction was quenched by addition of MeOH (5 mL). A mixture of MeCN and THF (1/1; 50 mL) was added, and the suspension was filtered over a celite plug, which was washed with MeCN/THF (3 x 50 mL). The filtrate was then concentrated onto silica, after which the desired product was obtained by automated flash column chromatography.

3. Table S1 - Full data overview for the catalyst screening

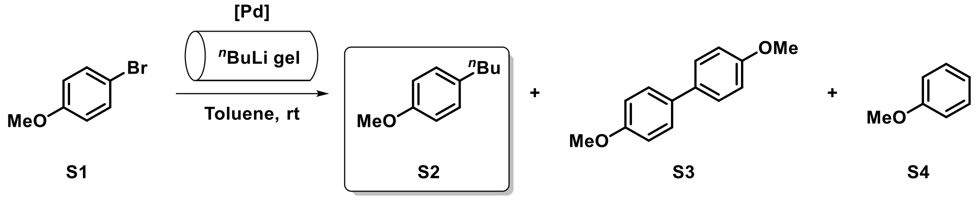

Entry[a]	[Pd]	[Pd] (mol%)	Time (min)	Conv. (%) S2:S3:S4[b]
1	-	-	5	1:0:35
2[c]				2:0:43
3	Pd PEPPSI IPr	5	10	75:0:14
4	Pd PEPPSI IPent			73:0:27
5	XPhos Pd G4			23:3:40
6	C1			11:0:38
7	$[Pd(\mu\text{-}I)P(^tBu)_3]_2$	2.5		37:2:22
8	$Pd[P(^tBu)_3]_2$	5		97:0:3
9		2.5		97:0:3
10		1.25		93:0:5
11		0.5		97:0:3
12			5	84:6:5
13		0.1	10	27:0:38
14	$Pd[P(^tBu)_3]_2/O_2$	5		96:4:0
15		2.5		94:0:6
16		1.25		96:1:3
17			5	95:1:4
18			1	96:0:4
19		1	1	92:1:6
20		0.5	10	86:0:7
21	Pd PEPPSI IPent^{Cl}			>99:0:0
22		2.5		95:0:3
23		1.25		99:0:1
24			5	>99:0:0
25			1	>99:0:0
**26**		**1**	**1**	**99:0:1**
27[c]				14:0:18
28[d]				99:0:1
29		0.5	10	90:0:8

Table S1. [a] Reaction conditions: A solution of the catalyst and **1** (0.3 mmol) in toluene (2 mL) was added to ^{n}BuLi gel (5 min pre-exposure to air at rt; 2.7 eq. ^{n}BuLi (1.6 M in hexanes), 0.5 mL hexanes, 4% wt/vol $C_{36}H_{74}$; 1 mL total volume. See GP-A for experimental details.) under air. The mixture was vigorously stirred, and quenched by addition of H_2O after the specified time. [b] Determined by gas chromatography-mass spectrometry analysis. [c] No gelation. [d] Gel formed with 2 eq. ^{n}BuLi (1.6 M in hexanes), 0.5 mL hexanes, 4% wt/vol $C_{36}H_{74}$; 1 mL total volume.

4. Table S2 – Full data overview for the optimization of the ^{n}BuLi gel characteristics

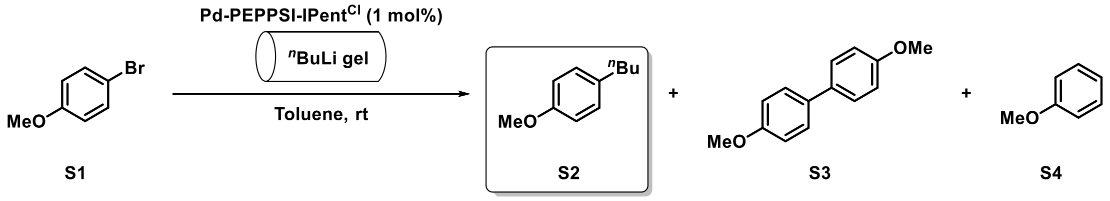

Entry[a]	^{n}BuLi (eq.)	[^{n}BuLi] (M)	$C_{36}H_{74}$ (% wt/vol)	Time (min)	Conv. (%) S2:S3:S4[b]
1[c]	2.7	0.80	4	1	99:0:1
2[d]					81:0:5
3				5	99:0:1
**4**	**2**	**0.60**			**99:0:1**
5	1.5	0.45			82:0:6
6[e]	2		2.7		99:0:1
7[e]		0.60	4		99:0:1

Table S2. [a] Reaction conditions: A solution of the catalyst and 1 (0.3 mmol) in toluene (2 mL) was added to ^{n}BuLi gel (5 min total pre-exposure to air; 2.7 eq. ^{n}BuLi (1.6 M in hexanes), 0.5 mL hexanes, 4% wt/vol $C_{36}H_{74}$; 1 mL total volume. See GP-A for experimental details.) under air. The mixture was vigorously stirred, and quenched by addition of H_2O after the specified time. [b] Determined by gas chromatography-mass spectrometry analysis. [c] 5 min pre-exposure to air at rt (≈ 22 °C). [d] 5 min pre-exposure to air at rt (≈ 25 °C). [e] 4 min pre-exposure to air at 0 °C, 1 min at rt.

Crucially, we observed that de-gelation of the gel could occur at ≈ 25 °C, resulting in diminished conversion, likely due to ^{n}BuLi hydrolysis (Entry 1 vs. 2, 22 °C vs. 25 °C). Equilibrating the gel to ambient atmosphere for 4 min at 0 °C, and 1 min at rt, restored reproducibility (Entry 3). The ^{n}BuLi loading could be lowered to 2 eq. (Entry 4), retaining near-quantitative conversion, although a further decrease reduced the conversion (Entry 5). Reactions performed using the minimum gelator concentration (2.7 wt/vol%)[4] were found to be irreproducible (Entry 6), while those performed using 4 wt/vol% $C_{36}H_{74}$ were consistently reproducible (Entry 7).

5. Formation and stability of organolithium gels

Methyllithium gel

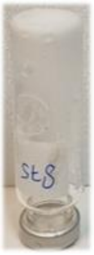

Formed according to GP-A from methyllithium solution (1.6 M in Et_2O; 0.64 mL; 1 mmol), ^{n}Hexane (1 mL) and $C_{36}H_{74}$ (66 mg; 4 wt/vol%). The obtained gel was found to be stable upon inversion, and over the course of the equilibration time.

^{n}Butyllithium gel

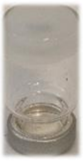

Formed according to GP-A from ^{n}butyllithium solution (1.6 M in hexanes; 0.64 mL; 1 mmol), ^{n}Hexane (1 mL) and $C_{36}H_{74}$ (66 mg; 4 wt/vol%). The obtained gel was found to be stable upon inversion, and over the course of the equilibration time.

^{n}Hexyllithium gel

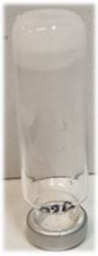

Formed according to GP-A from ^{n}hexyllithium solution (2.3 M in hexane; 0.44 mL; 1 mmol), ^{n}Hexane (1.2 mL) and $C_{36}H_{74}$ (66 mg; 4 wt/vol%). The obtained gel was found to be stable upon inversion, and over the course of the equilibration time.

Isobutyllithium gel

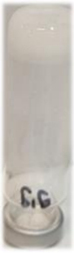

Formed according to GP-A from isobutyllithium solution (1.7 M in heptane; 0.59 mL; 1 mmol), ^{n}Hexane (1.05 mL) and $C_{36}H_{74}$ (66 mg; 4 wt/vol%). The obtained gel was found to be stable upon inversion, and over the course of the equilibration time.

^{s}Butyllithium gel

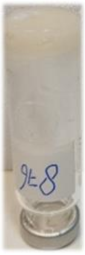

Formed according to GP-A from *sec*-butyllithium solution (1.6 M in cyclohexane; 0.72 mL; 1 mmol), ^{n}Hexane (0.92 mL) and $C_{36}H_{74}$ (66 mg; 4 wt/vol%). The obtained gel was found to be stable upon inversion, and over the course of the equilibration time.

^{t}Butyllithium gel

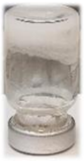

Formed according to GP-A from *tert*-butyllithium solution (1.7 M in pentane; 0.59 mL; 1 mmol), ^{n}Hexane (1.05 mL) and $C_{36}H_{74}$ (66 mg; 4 wt/vol%). The obtained gel was found to be stable upon inversion, and over the course of the equilibration time.

Phenyllithium gel

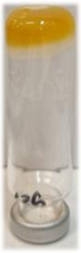

Formed according to GP-A from phenyllithium solution (1.9 M in Bu_2O; 0.53 mL; 1 mmol), ^{n}Hexane (1.11 mL) and $C_{36}H_{74}$ (66 mg; 4 wt/vol%). The obtained gel was found to be stable upon inversion, and over the course of the equilibration time.

Lithium (trimethylsilyl)acetylide gel

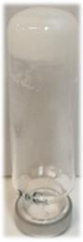

A 20 mL crimp-top vial was charged with hexatriacontane (66 mg; 4 wt/vol%) and a stirring egg, and was sealed. The vial was evacuated and back-filled with nitrogen thrice. Dry THF (0.22 mL), and (trimethylsilyl)acetylene (0.14 mL; 1 mmol) were added, after which the mixture was cooled to 0 °C. ^{n}Butyllithium solution (1.6 M in hexanes; 0.64 mL; 1 mmol) was then added dropwise, and the suspension was diluted with THF (0.64 mL; 1.64 mL total volume). The suspension was gently heated with a heat-gun until the gelator dissolved, after which the vial was cooled at 0 °C (ice bath) for 1 min to form the organogel. The obtained gel was found to be stable upon inversion, and over the course of the equilibration time.

^{n}Butyllithium block gel (0.5 mmol scale)

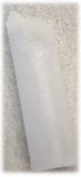

Formed according to GP-B from ^{n}butyllithium solution (1.6 M in hexanes; 0.64 mL; 1 mmol), ^{n}Hexane (1 mL) and $C_{36}H_{74}$ (66 mg; 4 wt/vol%). The obtained gel was found to be stable upon inversion, and over the course of the equilibration time.

6. Substrates incompatible with the reaction conditions

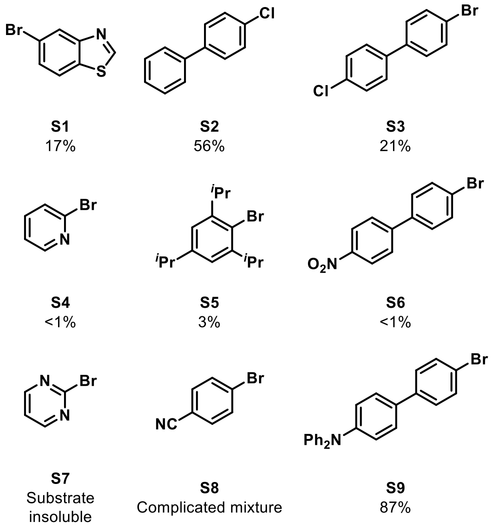

Table S2. Substrates that were found to be incompatible with the reaction conditions. Conversions determined by gas chromatography-mass spectrometry analysis are given.

S1 afforded 17% conversion, along with several unidentified products. The reaction of biphenyl chloride **S2** resulted in catalyst poisoning (56% conversion after 5 min, 58% conversion after 15 min). The attempted chemoselective cross-coupling in the presence of a chloride (**S3**) afforded only 21% of the desired product (60% di-coupling). Attempted cross-coupling of pyridine **S4** did not afford the desired product, while sterically encumbered tri(isopropyl)bromobenzene **S5** was unreactive under the reaction conditions. The product derived from **S6** could not be observed by GC/MS analysis. Pyrimidine **S7** was insoluble under the reaction conditions. The attempted cross-coupling of **S8** afforded a complicated mixture. Although the reaction with diphenylamine-substituted substrate **S9** proceeded efficiently, the product was chromatographically inseparable from an unidentified impurity.

7. Synthesis of products

1-Butyl-4-methoxybenzene (5)

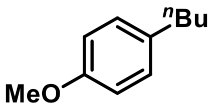

CAS Registry Number: 18272-84-9

Synthesized using GP-A 4-bromoanisole (94 mg) in the presence of 1 mol% Pd-PEPPSI-IPent^{Cl}. The product was obtained as a clear liquid (54 mg; 66%) after purification by automated flash column chromatography (^{n}Pentane to ^{n}Pentane/Et_2O 99/1). ^{1}H NMR (400 MHz, $CDCl_3$) δ 7.12 – 7.07 (m, 2H), 6.83 (dd, *J* = 8.6, 1.3 Hz, 2H), 3.79 (s, 3H), 2.55 (t, *J* = 7.7 Hz, 2H), 1.62 – 1.51 (m, 2H), 1.35 (h, *J* = 7.4 Hz, 2H), 0.92 (td, *J* = 7.4, 1.3 Hz, 3H). ^{13}C NMR (151 MHz, $CDCl_3$) δ 157.69, 135.11, 129.35, 113.74, 55.33, 34.83, 34.03, 22.41, 14.07. Data in accordance with the literature.[5]

1-Butyl-4-(trifluoromethyl)benzene (6)

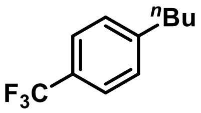

CAS Registry Number: 61342-04-9

Synthesized using GP-A from 4-bromobenzotrifluoride (113 mg) in the presence of 1 mol% Pd-PEPPSI-IPent^{Cl}. The product was obtained as a clear liquid (19 mg; 19%) after purification by automated flash column chromatography (^{n}Pentane). ^{1}H NMR (400 MHz, $CDCl_3$) δ 7.53 (d, *J* = 8.0 Hz, 2H), 7.28 (d, *J* = 8.0 Hz, 2H), 2.67 (t, *J* = 7.8 Hz, 2H), 1.68 – 1.56 (m, 2H), 1.36 (h, *J* = 7.3 Hz, 2H), 0.94 (t, *J* = 7.3 Hz, 3H). ^{13}C NMR (151 MHz, $CDCl_3$) δ 147.14, 128.83, 128.12 (q, *J* = 32.2 Hz), 125.29 (q, *J* = 3.8 Hz), 124.58 (q, *J* = 271.6 Hz), 35.64, 33.48, 22.42, 14.03. ^{19}F NMR (376 MHz, $CDCl_3$) δ -62.27. Data in accordance with the literature.[5]

1-Butyl-4-methylbenzene (7)

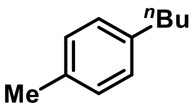

CAS Registry Number: 1595-05-7

Synthesized using GP-A from 1-bromo-4-methylbenzene (86 mg) in the presence of 1 mol% Pd-PEPPSI-IPent^{Cl}. The product was obtained as a clear liquid (20 mg; 27%) after purification by automated flash column chromatography (^{n}Pentane). ^{1}H NMR (400 MHz, $CDCl_3$) δ 7.09 (s, 4H), 2.58 (t, *J* = 7.7 Hz, 2H), 2.33 (s, 3H), 1.66 – 1.54 (m, 2H), 1.36 (h, *J* = 7.4 Hz, 2H), 0.93 (td, *J* = 7.3, 1.0 Hz, 3H). ^{13}C NMR (151 MHz, $CDCl_3$) δ 139.98, 135.08, 129.05, 128.43, 35.35, 33.95, 22.52, 21.13, 14.11. Data in accordance with the literature.[5]

4-Butyl-*N,N*-dimethylaniline (8)

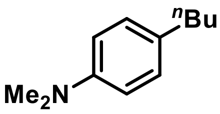

CAS Registry Number: 13330-29-5

Synthesized using GP-A from 4-bromo-*N,N*-dimethylaniline (100 mg) in the presence of 1 mol% Pd-PEPPSI-IPent^{Cl}. The product was obtained as a clear liquid (67 mg; 76%) after purification by automated flash column chromatography (^{n}Pentane to ^{n}Pentane/EtOAc 95/5). ^{1}H NMR (400 MHz, $CDCl_3$) δ 7.07 (d, *J* = 8.6 Hz, 2H), 6.71 (d, *J* = 8.6 Hz, 2H), 2.91 (s, 6H), 2.68 – 2.32 (m, 2H), 1.63 – 1.51 (m, 2H), 1.35 (h, *J* = 7.3 Hz, 2H), 0.92 (t, *J* = 7.3 Hz, 3H). ^{13}C NMR (101 MHz, $CDCl_3$) δ 149.08, 131.41, 129.10, 113.17, 41.13, 34.73, 34.13, 22.52, 14.14. Data in accordance with the literature.[5]

1-Butyl-2-isopropylbenzene (9)

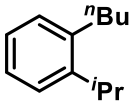

CAS Registry Number: 55169-01-2

Synthesized using GP-A from 1-bromo-2-isopropylbenzene (100 mg) using 1 mol% Pd-PEPPSI-IPent^{Cl}. The product was obtained as a clear liquid (64 mg; 73%) after purification by automated flash column chromatography (^{n}Pentane). ^{1}H NMR (400 MHz, $CDCl_3$) δ 7.30 – 7.23 (m, 1H), 7.18 (td, *J* = 7.1, 2.2 Hz, 1H), 7.15 – 7.07 (m, 1H), 3.18 (hept, *J* = 6.9 Hz, 1H), 2.69 – 2.60 (m, 1H), 1.62 – 1.50 (m, 2H), 1.41 (h, *J* = 7.3 Hz, 2H), 1.24 (d, *J* = 6.9 Hz, 4H), 0.95 (t, *J* = 7.3 Hz, 2H). ^{13}C NMR (101 MHz, $CDCl_3$) δ 146.63, 139.76, 129.49, 126.23, 125.60, 125.32, 34.16, 32.78, 28.68, 24.21, 22.98, 14.18. Data in accordance with the literature.[6]

1-Butylnaphthalene (10)

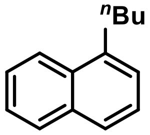

CAS Registry Number: 1634-09-9

Synthesized using GP-A from 1-bromonaphthalene (104 mg) in the presence of 1 mol% Pd-PEPPSI-IPent^{Cl}. The product was obtained as a clear oil (80 mg; 87%) after purification by automated flash column chromatography (^{n}Pentane). ^{1}H NMR (400 MHz, $CDCl_3$) δ 8.11 (d, *J* = 8.2 Hz, 1H), 7.93 – 7.88 (m, 1H), 7.76 (d, *J* = 8.2 Hz, 1H), 7.54 (dqd, *J* = 8.1, 6.7, 1.4 Hz, 2H), 7.49 – 7.42 (m, 1H), 7.38 (d, *J* = 7.0 Hz, 1H), 3.16 – 3.10 (m, 2H), 1.86 – 1.74 (m, 2H), 1.52 (h, *J* = 7.4 Hz, 2H), 1.08 – 1.00 (m, 3H). ^{13}C NMR (101 MHz, $CDCl_3$) δ 139.14, 134.04, 132.07, 128.88, 126.52, 125.99, 125.74, 125.67, 125.48, 124.06, 33.16, 32.96, 23.03, 14.16. Data in accordance with the literature.[5]

1-Butyl-2-methylnaphthalene (11)

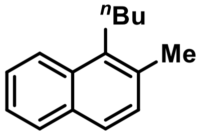

CAS Registry Number: 39036-72-1

Synthesized using GP-A from 1-bromo-2-methylnaphthalene (113 mg). The product was obtained as a clear liquid (61 mg; 62%) after purification by automated flash column chromatography (^{n}Pentane). ^{1}H NMR (400 MHz, $CDCl_3$) δ 8.04 (d, *J* = 8.6 Hz, 1H), 7.81 (dd, *J* = 8.1, 2.0 Hz, 1H), 7.63 (d, *J* = 8.4 Hz, 1H), 7.49 (ddd, *J* = 8.6, 6.8, 1.9 Hz, 1H), 7.42 (td, *J* = 6.8, 3.5 Hz, 1H), 7.31 (dt, *J* = 8.3, 1.8 Hz, 1H), 3.21 – 2.92 (m, 2H), 2.51 (s, 3H), 1.68 – 1.59 (m, 2H), 1.59 – 1.51 (m, 2H), 1.02 (ddd, *J* = 7.2, 5.2, 2.0 Hz, 3H). ^{13}C NMR (101 MHz, $CDCl_3$) δ 159.04, 141.56, 138.29, 133.92, 128.93, 128.12, 126.71, 114.27, 55.48, 35.41, 33.83, 22.56, 14.13. Data in accordance with the literature.[7]

9-Butylphenanthrene (12)

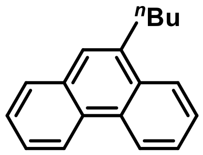

CAS Registry Number: 10394-57-7

Synthesized using GP-A from 9-bromophenanthrene (129 mg). The product was obtained as a white solid (96 mg; 82%) after purification by automated flash column chromatography (^{n}Pentane). ^{1}H NMR (400 MHz, $CDCl_3$) δ 8.81 – 8.71 (m, 1H), 8.66 (d, *J* = 7.8 Hz, 1H), 8.16 – 8.09 (m, 1H), 7.83 (dd, *J* = 7.0, 2.1 Hz, 1H), 7.69 – 7.61 (m, 2H), 7.61 – 7.53 (m, 3H), 3.20 – 2.91 (m, 2H), 1.81 (p, *J* = 7.6 Hz, 2H), 1.57 – 1.45 (m, 3H), 1.03 – 0.97 (m, 3H). ^{13}C NMR (101 MHz, $CDCl_3$) δ 136.96, 131.95, 131.34, 130.69, 129.58, 127.98, 126.54, 126.42, 126.04, 125.94, 125.81, 124.49, 123.19, 122.42. The obtained yield was corrected for the presence of 4% ^{s}Bu-isomer.[8] Data in accordance with the literature.[9]

2-Butyl-1,1'-biphenyl (13)

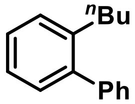

CAS Registry Number: 54532-97-7

Synthesized using GP-A from 2-bromobiphenyl (117 mg). The product was obtained as a clear liquid (75 mg; 72%) after purification by automated flash column chromatography (^{n}Pentane). ^{1}H NMR (400 MHz, $CDCl_3$) δ 7.44 – 7.38 (m, 2H), 7.37 – 7.34 (m, 1H), 7.34 – 7.28 (m, 4H), 7.25 – 7.19 (m, 2H), 2.63 – 2.54 (m, 2H), 1.50 – 1.41 (m, 2H), 1.22 (h, J = 7.3 Hz, 2H), 0.80 (t, J = 7.3 Hz, 3H). ^{13}C NMR (101 MHz, $CDCl_3$) δ 142.19, 141.98, 140.48, 130.15, 129.40, 129.36, 128.10, 127.43, 126.83, 125.64, 33.71, 32.82, 22.64, 13.97. Data in accordance with the literature.[10]

3-Butyl-1,1'-biphenyl (14)

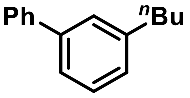

CAS Registry Number: 81782-75-4

Synthesized using GP-A from 3-bromobiphenyl (117 mg). The product was obtained as a clear liquid (80 mg; 76%) after purification by automated flash column chromatography (^{n}Pentane). ^{1}H NMR (400 MHz, $CDCl_3$) δ 7.64 – 7.57 (m, 2H), 7.48 – 7.40 (m, 4H), 7.35 (dtd, J = 8.6, 5.9, 2.6 Hz, 2H), 7.18 (d, J = 7.5 Hz, 1H), 2.73 – 2.64 (m, 2H), 1.72 – 1.60 (m, 2H), 1.40 (ddt, J = 15.3, 8.4, 6.2 Hz, 2H), 0.95 (td, J = 7.3, 1.8 Hz, 3H). ^{13}C NMR (101 MHz, $CDCl_3$) δ 143.53, 141.62, 141.33, 128.83, 128.77, 127.53, 127.50, 127.35, 127.27, 124.64, 35.92, 33.87, 22.57, 14.13. Data in accordance with the literature.[11]

4-Butyl-1,1'-biphenyl (15)

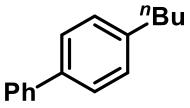

CAS Registry Number: 37909-95-8

Synthesized using GP-A from 4-bromobiphenyl (117 mg). The product was obtained as a clear liquid (96 mg; 91%) after purification by automated flash column chromatography (^{n}Pentane). ^{1}H NMR (400 MHz, $CDCl_3$) δ 7.62 – 7.59 (m, 2H), 7.56 – 7.52 (m, 2H), 7.47 – 7.42 (m, 2H), 7.37 – 7.31 (m, 1H), 7.28 (d, J = 7.9 Hz, 2H), 2.72 – 2.62 (m, 2H), 1.66 (tt, J = 7.8, 6.4 Hz, 2H), 1.42 (h, J = 7.3 Hz, 2H), 0.97 (t, J = 7.3 Hz, 3H). ^{13}C NMR (101 MHz, $CDCl_3$) δ 142.22, 141.35, 138.69, 128.97, 128.84, 127.14, 127.13, 127.09, 35.45, 33.80, 22.57, 14.13. Data in accordance with the literature.[12]

4-Butyl-4'-methoxy-1,1'-biphenyl (16)

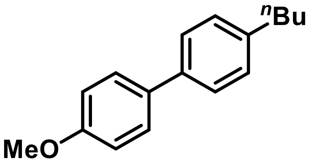

CAS Registry Number: 35684-16-3

Synthesized using GP-A from 4-bromo-4'-methoxybiphenyl (132 mg). The product was obtained as a white solid (109 mg; 91%) after purification by automated flash column chromatography (^{n}Pentane). ^{1}H NMR (400 MHz, $cdcl_3$) δ 7.52 (d, J = 8.6 Hz, 2H), 7.47 (d, J = 8.1 Hz, 2H), 7.23 (d, J = 8.0 Hz, 2H), 6.97 (d, J = 8.7 Hz, 2H), 3.85 (s, 3H), 2.69 – 2.57 (m, 2H), 1.63 (p, J = 7.6 Hz, 2H), 1.39 (h, J = 7.4 Hz, 2H), 0.95 (t, J = 7.3 Hz, 3H). ^{13}C NMR (151 MHz, $CDCl_3$) δ 159.05, 141.57, 138.30, 133.93, 128.93, 128.12, 126.71, 114.28, 55.48, 35.41, 33.82, 22.56, 14.13. Data in accordance with the literature.[13]

4-Butyl-4'-methyl-1,1'-biphenyl (17)

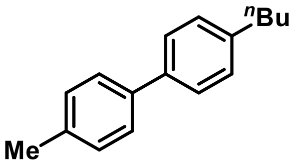

CAS Registry Number: 77117-36-3

Synthesized using GP-A from 4-bromo-4'-methylbiphenyl (124 mg). The product was obtained as a white solid (105 mg; 93%) after purification by automated flash column chromatography (ⁿPentane). ^{1}H NMR (400 MHz, $CDCl_3$) δ 7.49 (dd, *J* = 7.8, 4.5 Hz, 4H), 7.24 (d, *J* = 7.9 Hz, 4H), 2.65 (t, *J* = 7.8 Hz, 3H), 1.64 (p, *J* = 7.6 Hz, 0H), 1.39 (h, *J* = 7.4 Hz, 0H), 0.99 – 0.91 (m, 1H). ^{13}C NMR (101 MHz, $CDCl_3$) δ 141.90, 138.61, 138.46, 136.81, 129.56, 128.93, 126.97, 126.93, 35.44, 33.82, 22.57, 21.23, 14.13. Data in accordance with the literature.[14]

4-Butyl-4'-methyl-1,1'-biphenyl (18)

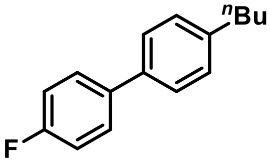

CAS Registry Number: 546109-44-8

Synthesized using GP-A from 4-bromo-4'-fluorobiphenyl (126 mg). The product was obtained as clear crystals (89 mg; 72%) after purification by automated flash column chromatography (^{n}Pentane). ^{1}H NMR (400 MHz, $CDCl_3$) δ 7.56 – 7.49 (m, 2H), 7.45 (d, *J* = 8.2 Hz, 1H), 7.26 – 7.23 (m, 1H), 7.11 (t, *J* = 8.7 Hz, 1H), 2.69 – 2.62 (m, 2H), 1.69 – 1.58 (m, 2H), 1.39 (h, *J* = 7.3 Hz, 2H), 0.95 (t, *J* = 7.4 Hz, 3H). ^{13}C NMR (151 MHz, $CDCl_3$) δ 162.43 (d, *J* = 245.7 Hz), 139.98 (d, *J* = 682.2 Hz), 137.44 (d, *J* = 3.2 Hz), 129.03, 128.62 (d, *J* = 8.1 Hz), 126.98, 115.67 (d, *J* = 21.3 Hz), 35.41, 33.79, 22.54, 14.12. ^{19}F NMR (376 MHz, $cdcl_3$) δ -116.38. Data in accordance with the literature.[15]

4'-Butyl-[1,1'-biphenyl]-4-yl trifluoromethanesulfonate (19)

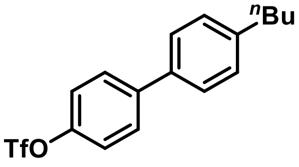

Synthesized using GP-A from 4'-bromo-[1,1'-biphenyl]-4-yl trifluoromethanesulfonate (191 mg) in the presence of 2.5 mol% $Pd[P(^tBu)_3]_2/O_2$ as catalyst. The product was obtained as a colorless solid (61 mg; 34%) after purification by automated flash column chromatography (^{n}Pentane). ^{1}H NMR (400 MHz, $CDCl_3$) δ 7.63 (d, *J* = 9.1 Hz, 2H), 7.47 (d, *J* = 7.8 Hz, 2H), 7.32 (d, *J* = 8.6 Hz, 2H), 7.27 (d, *J* = 8.7 Hz, 2H), 2.66 (t, *J* = 7.8 Hz, 2H), 1.70 – 1.58 (m, 2H), 1.39 (h, *J* = 7.4 Hz, 2H), 0.99 – 0.90 (m, 3H). ^{13}C NMR (151 MHz, CDCl3) δ 148.87, 143.17, 141.82, 136.73, 129.21, 128.80, 127.16, 121.70, 118.93 (q, J = 320.7 Hz), 35.43, 33.72, 22.52, 14.10. ^{19}F NMR (376 MHz, $CDCl_3$) δ -72.81. HRMS (APCI+, *m/z*): calculated for $C_{17}H_{18}F_3O_3$ $[M+H]^+$: 358.08466; found: 358.08450.

5-Butyl-1-methyl-1*H*-indole (20)

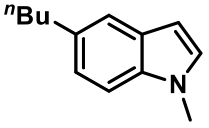

CAS Registry Number: 1683589-10-7

Reaction performed on 0.5 mmol scale: Synthesized using GP-A from 5-bromo-1-methyl-1*H*-indole (105 mg) in the presence of 1 mol% Pd-PEPPSI-IPent^{Cl}. The product was obtained as a pale yellow liquid (83 mg; 89%) after purification by automated flash column chromatography (^{n}Pentane to ^{n}Pentane/EtOAc 95/5). ^{1}H NMR (400 MHz, $CDCl_3$) δ 7.43 (dd, J = 1.6, 0.8 Hz, 1H), 7.26 – 7.22 (m, 1H), 7.08 (dd, J = 8.4, 1.7 Hz, 1H), 7.02 (d, J = 3.1 Hz, 1H), 3.78 (s, 3H), 2.80 – 2.63 (m, 2H), 1.71 – 1.60 (m, 2H), 1.39 (dq, J = 14.7, 7.3 Hz, 2H), 0.94 (t, J = 7.3 Hz, 3H). ^{13}C NMR (101 MHz, $CDCl_3$) δ 135.26, 133.74, 128.79, 128.59, 122.57, 119.94, 108.83, 100.38, 35.72, 34.54, 32.84, 22.40, 14.05. Data in accordance with the literature.[5]

Reaction performed on 5 mmol scale: Synthesized using GP-B from 5-bromo-1-methyl-1*H*-indole (1.05 g). The product was obtained as a pale-yellow liquid (730 mg; 78%) after purification by automated flash column chromatography (^{n}Pentane to ^{n}Pentane/EtOAc 95/5). Spectral data matches that reported for the 0.5 mmol scale reaction.

4-(4-Butylphenyl)morpholine (21)

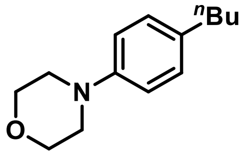

CAS Registry Number: 564483-41-6

Synthesized using GP-A from 4-(4-bromophenyl)morpholine (121 mg) in the presence of 1 mol% Pd-PEPPSI-IPent^{Cl}. The product was obtained as a pink-white solid (69 mg; 63%) after purification by automated flash column chromatography (^{n}Pentane to ^{n}Pentane/EtOAc 95/5). ^{1}H NMR (400 MHz, $CDCl_3$) δ 7.10 (d, *J* = 8.4 Hz, 2H), 6.85 (d, *J* = 8.5 Hz, 2H), 3.86 (dd, *J* = 5.9, 3.7 Hz, 4H), 3.16 – 3.09 (m, 4H), 2.58 – 2.50 (m, 2H), 1.56 (p, *J* = 7.5 Hz, 2H), 1.34 (h, *J* = 7.3 Hz, 2H), 0.96 – 0.88 (m, 3H). ^{13}C NMR (101 MHz, $CDCl_3$) δ 149.42, 134.82, 129.21, 116.01, 67.16, 49.95, 34.83, 33.95, 22.49, 14.12. Data in accordance with the literature.[16]

9-(4-Butylphenyl)-9*H*-carbazole (22)

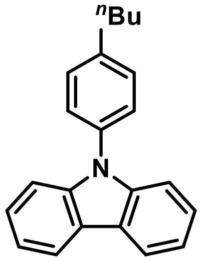

CAS Registry Number: 951659-74-8

Synthesized using GP-A from 9-(4-bromophenyl)-9*H*-carbazole (124 mg). The product was obtained as a white solid (122 mg; 81%) after purification by automated flash column chromatography (^{n}Pentane). ^{1}H NMR (400 MHz, $CDCl_3$) δ 8.15 (d, *J* = 7.7 Hz, 2H), 7.46 (d, *J* = 8.3 Hz, 2H), 7.44 – 7.38 (m, 6H), 7.32 – 7.24 (m, 2H), 2.79 – 2.71 (m, 2H), 1.78 – 1.66 (m, 2H), 1.46 (h, *J* = 7.4 Hz, 2H), 1.00 (t, *J* = 7.3 Hz, 3H). ^{13}C NMR (101 MHz, $CDCl_3$) δ 142.49, 141.21, 135.28, 129.91, 127.09, 125.96, 123.38, 120.38, 119.84, 109.98, 35.54, 33.77, 22.59, 14.15. Data in accordance with the literature.[17]

4-Butyldibenzo[*b,d*]furan (23)

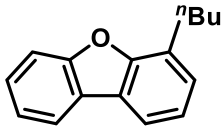

CAS Registry Number: 2086689-83-8

Synthesized using GP-A from 4-bromodibenzofuran (124 mg). The product was obtained as a clear oil (68 mg; 61%) after purification by automated flash column chromatography (^{n}Pentane). ^{1}H NMR (400 MHz, $CDCl_3$) δ 7.95 (d, *J* = 7.7 Hz, 1H), 7.80 (dd, *J* = 5.9, 3.6 Hz, 1H), 7.60 (d, *J* = 8.2 Hz, 1H), 7.50 – 7.41 (m, 1H), 7.34 (t, *J* = 7.5 Hz, 1H), 7.31 – 7.26 (m, 2H), 3.00 (t, *J* = 7.8 Hz, 2H), 1.86 – 1.74 (m, 2H), 1.46 (h, *J* = 7.4 Hz, 2H), 1.03 – 0.94 (m, 3H). ^{13}C NMR (101 MHz, $CDCl_3$) δ 156.16, 154.94, 127.36, 127.02, 126.99, 124.80, 123.90, 122.79, 122.63, 120.79, 118.16, 111.80, 32.21, 29.76, 22.71, 14.12. Data in accordance with the literature.[3]

4-Hexyl-1,1'-biphenyl (25)

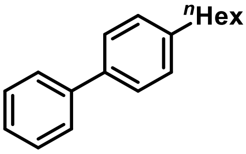

CAS Registry Number: 59662-31-6

Synthesized using GP-A from 4-bromobiphenyl (117 mg). The product was obtained as a white solid (92 mg; 77%) after purification by automated flash column chromatography (^{n}Pentane). ^{1}H NMR (400 MHz, $CDCl_3$) δ 7.62 – 7.57 (m, 2H), 7.56 – 7.49 (m, 2H), 7.48 – 7.40 (m, 2H), 7.38 – 7.30 (m, 1H), 7.29 – 7.25 (m, 3H), 2.69 – 2.63 (m, 2H), 1.70 – 1.61 (m, 2H), 1.42 – 1.29 (m, 6H), 0.93 – 0.87 (m, 3H). ^{13}C NMR (101 MHz, $CDCl_3$) δ 142.12, 141.19, 138.52, 128.82, 128.69, 126.99, 126.98, 126.94, 35.63, 31.76, 31.49, 29.07, 22.64, 14.13. Data in accordance with the literature.[18]

4-Isobutyl-1,1'-biphenyl (26)

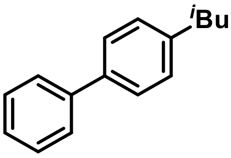

CAS Registry Number: 10468-83-4

Synthesized using GP-A from 4-bromobiphenyl (117 mg). The product was obtained as an off-white solid (37 mg; 35%) after purification by automated flash column chromatography (^{n}Pentane). ^{1}H NMR (400 MHz, $CDCl_3$) δ 7.63 – 7.55 (m, 2H), 7.55 – 7.47 (m, 2H), 7.43 (dd, *J* = 8.5, 6.9 Hz, 2H), 7.35 – 7.29 (m, 1H), 7.25 – 7.19 (m, 2H), 2.52 (d, *J* = 7.2 Hz, 2H), 1.91 (dp, *J* = 13.5, 6.8 Hz, 1H), 0.94 (d, *J* = 6.6 Hz, 6H). ^{13}C NMR (101 MHz, $CDCl_3$) δ 141.33, 141.00, 138.70, 129.68, 128.84, 127.13, 127.09, 126.96, 45.23, 30.40, 22.57. Data in accordance with the literature.[19]

4-(Sec-butyl)-1,1'-biphenyl (27)

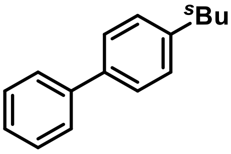

CAS Registry Number: 16236-40-1

Synthesized using GP-A from 4-bromobiphenyl (117 mg). The product was obtained as a pale-yellow liquid (91 mg; 87%) after purification by automated flash column chromatography (^{n}Pentane). ^{1}H NMR (400 MHz, $CDCl_3$) δ 7.63 – 7.56 (m, 2H), 7.53 (d, *J* = 8.0 Hz, 2H), 7.43 (t, *J* = 7.7 Hz, 2H), 7.35 – 7.30 (m, 1H), 7.26 (d, *J* = 8.1 Hz, 2H), 2.65 (h, *J* = 7.0 Hz, 1H), 1.64 (pd, *J* = 7.3, 1.6 Hz, 2H), 1.28 (d, *J* = 7.0 Hz, 3H), 0.87 (t, *J* = 7.4 Hz, 3H). ^{13}C NMR (151 MHz, $CDCl_3$) δ 146.99, 141.34, 138.84, 128.82, 127.61, 127.14, 127.12, 127.07, 41.49, 31.32, 21.96, 12.43. Data in accordance with the literature.[20]

1,1':4',1''-Terphenyl (30)

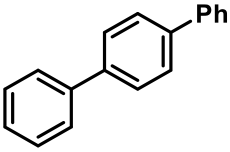

CAS Registry Number: 92-94-4

Synthesized using GP-A from 4-bromobiphenyl (117 mg) using 5 mol% Pd-PEPPSI-IPent^{Cl} as catalyst. The product was obtained as a white solid (61 mg; 53%) after purification by automated flash column chromatography (^{n}Pentane). ^{1}H NMR (400 MHz, $CDCl_3$) δ 7.69 (s, 4H), 7.68 – 7.63 (m, 4H), 7.50 – 7.44 (m, 4H), 7.40 – 7.34 (m, 2H). ^{13}C NMR (101 MHz, $CDCl_3$) δ 140.85, 140.27, 128.97, 127.65, 127.49, 127.20. Data in accordance with the literature.[21]

3-Butylbenzo[*b*]thiophene (32)

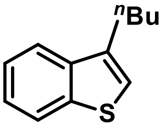

CAS Registry Number: 105230-36-2

Synthesized using GP-A from 3-bromothianaphthene (106.6 mg). The product was obtained as a clear liquid (63 mg; 66%) after purification by automated flash column chromatography (^{n}Pentane). ^{1}H NMR (400 MHz, $CDCl_3$) δ 7.89 – 7.83 (m, 1H), 7.78 – 7.74 (m, 1H), 7.36 (dtd, *J* = 17.7, 7.1, 1.3 Hz, 2H), 7.08 (d, *J* = 1.1 Hz, 1H), 2.89 – 2.81 (m, 2H), 1.80 – 1.69 (m, 2H), 1.46 (h, *J* = 7.4 Hz, 2H), 0.98 (t, *J* = 7.3 Hz, 3H). ^{13}C NMR (101 MHz, $CDCl_3$) δ 140.63, 139.30, 137.36, 124.19, 123.86, 123.00, 121.87, 120.93, 31.47, 28.43, 22.80, 14.10. Data in accordance with the literature.[3]

8. Supplementary References

[1] M. Aufiero, T. Sperger, A. S.-K. Tsang and F. Schoenebeck, *Angew. Chem. Int. Ed.*, 2015, **54**, 10322-10326.

[2] M. K. Kolli, N. M. Shaik, G. Chandrasekar, S. Chidara and R. B. Korupolu, *New J. Chem.*, 2017, **41**, 8187-8195.

[3] D. Heijnen, F. Tosi, C. Vila, M. C. A. Stuart, P. H. Elsinga, W. Szymanski and B. L. Feringa, *Angew. Chem. Int. Ed.*, 2017, **56**, 3354-3359.

[4] P. Slavík, B. R. Trowse, P. O'Brien and D. Smith, *Nat. Chem.*, 2023, DOI: 10.1038/s41557-023-01136-x.

[5] G. Dilauro, A. Francesca Quivelli, P. Vitale, V. Capriati and F. M. Perna, *Angew. Chem. Int. Ed.*, 2019, **58**, 1799-1802.

[6] T. Agrawal and S. P. Cook, *Org. Lett.*, 2013, **15**, 96-99.

[7] R. Muntwyler and W. Keller-Schierlein, *Helv. Chim. Acta*, 1972, **55**, 2071-2094.

[8] N. Sinha, D. Heijnen, B. L. Feringa and M. G. Organ, *Chem. Eur. J.*, 2019, **25**, 9180-9184.

[9] A. Piontek and M. Szostak, *Eur. J. Org. Chem.*, 2017, 7271-7276.

[10] I. Kondolff, H. Doucet and M. Santelli, *Tetrahedron*, 2004, **60**, 3813-3818.

[11] B. Shao, A. L. Bagdasarian, S. Popov and H. M. Nelson, *Science*, 2017, **355**, 1403-1407.

[12] D. Heijnen, J.-B. Gualtierotti, V. Hornillos and B. L. Feringa, *Chem. Eur. J.*, 2016, **22**, 3991-3995.

[13] Y.-B. Zhou, C.-Y. Li, M. Lin, Y.-J. Ding and Z.-P. Zhan, *Adv. Synth. Catal.*, 2015, **357**, 2503-2508.

[14] S. B. Blakey and D. W. C. MacMillan, *J. Am. Chem. Soc.*, 2003, **125**, 6046-6047.

[15] T. Hatakeyama, S. Hashimoto, K. Ishizuka and M. Nakamura, *J. Am. Chem. Soc.*, 2009, **131,** 11949-11963.

[16] J. D. Shields, E. E. Gray and A. G. Doyle, *Org. Lett.*, 2015, **17,** 2166-2169.

[17] H. N. Do, N. M. Quan, B. Van Phuc, D. Van Tinh, N. Q. Tien, T. T. T. Nga, V. T. Nguyen, T. Q. Hung, T. T. Dang and P. Langer, *Synlett*, 2021, **32**, 611-615.

[18] Z. Li, L. Liu, H.-m. Sun, Q. Shen and Y. Zhang, *Dalton Trans.*, 2016, **45**, 17739-17747.

[19] E. Brunard, V. Boquet, E. Van Elslande, T. Saget and P. Dauban, *J. Am. Chem. Soc.*, 2021, **143**, 6407-6412.

[20] W. Liu, J. Li, P. Querard and C.-J. Li, *J. Am. Chem. Soc.*, 2019, **141**, 6755-6764.

[21] N.-N. Ma, J.-A. Ren, X. Liu, X.-Q. Chu, W. Rao and Z.-L. Shen, *Org. Lett.*, 2022, **24**, 1953-1957.

9. Spectral data

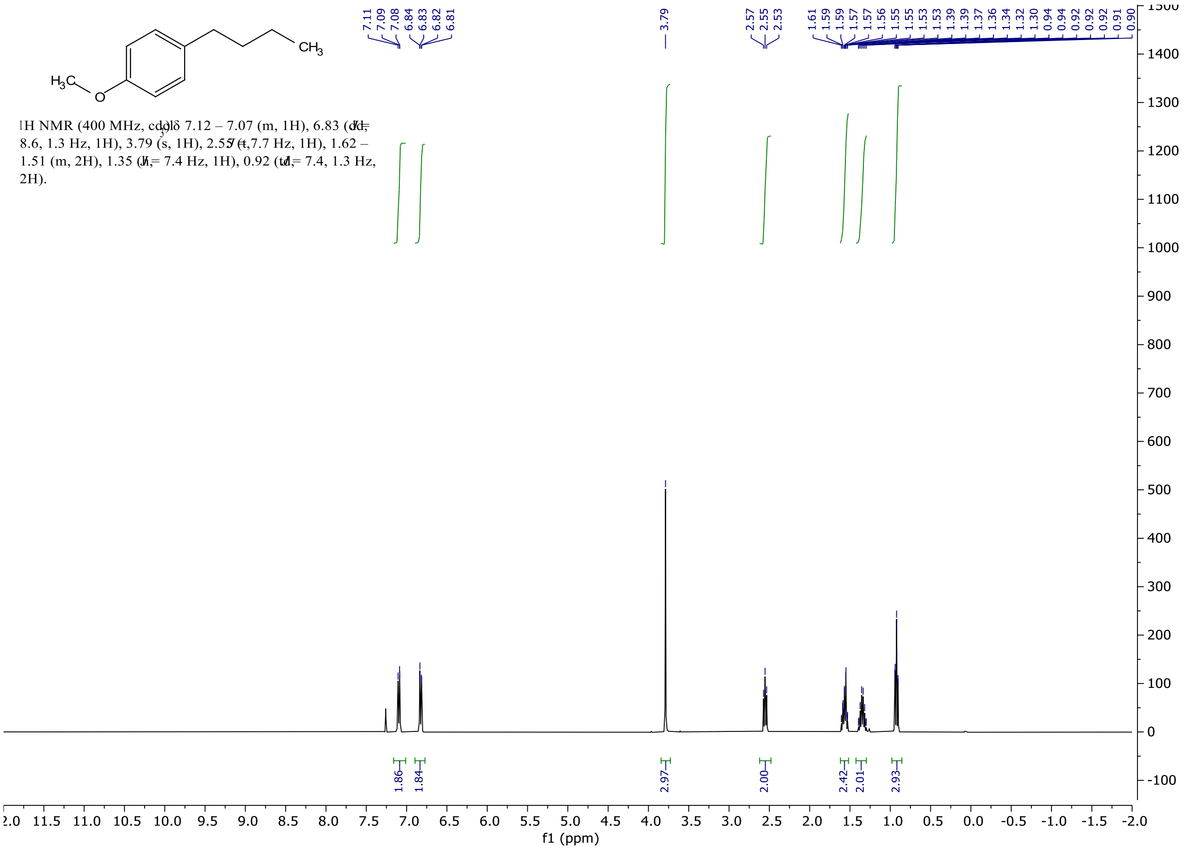

^{1}H NMR (400 MHz, $cdcl_3$) δ 7.12 – 7.07 (m, 1H), 6.83 (dd, J = 8.6, 1.3 Hz, 1H), 3.79 (s, 1H), 2.55 (t, J = 7.7 Hz, 1H), 1.62 – 1.51 (m, 2H), 1.35 (h, J = 7.4 Hz, 1H), 0.92 (td, J = 7.4, 1.3 Hz, 2H).

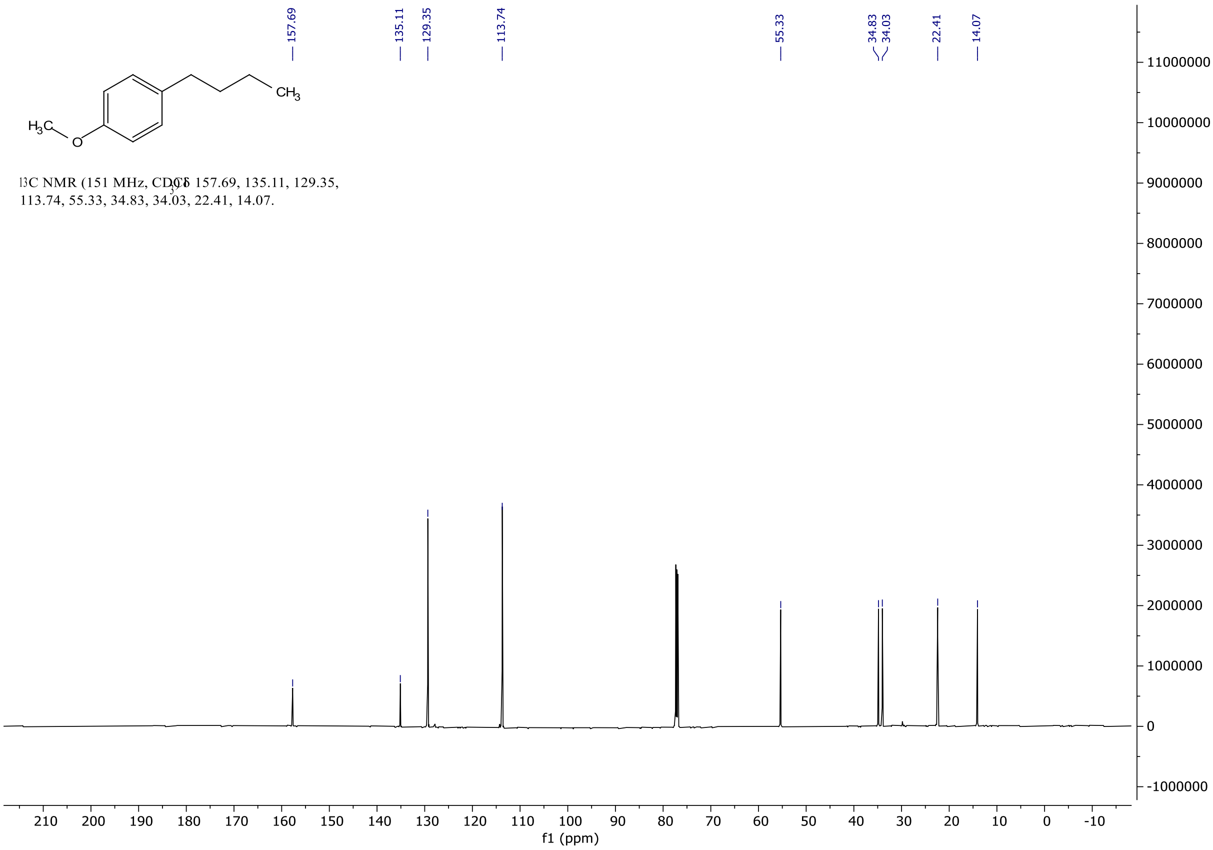

^{13}C NMR (151 MHz, $CDCl_3$) δ 157.69, 135.11, 129.35, 113.74, 55.33, 34.83, 34.03, 22.41, 14.07.

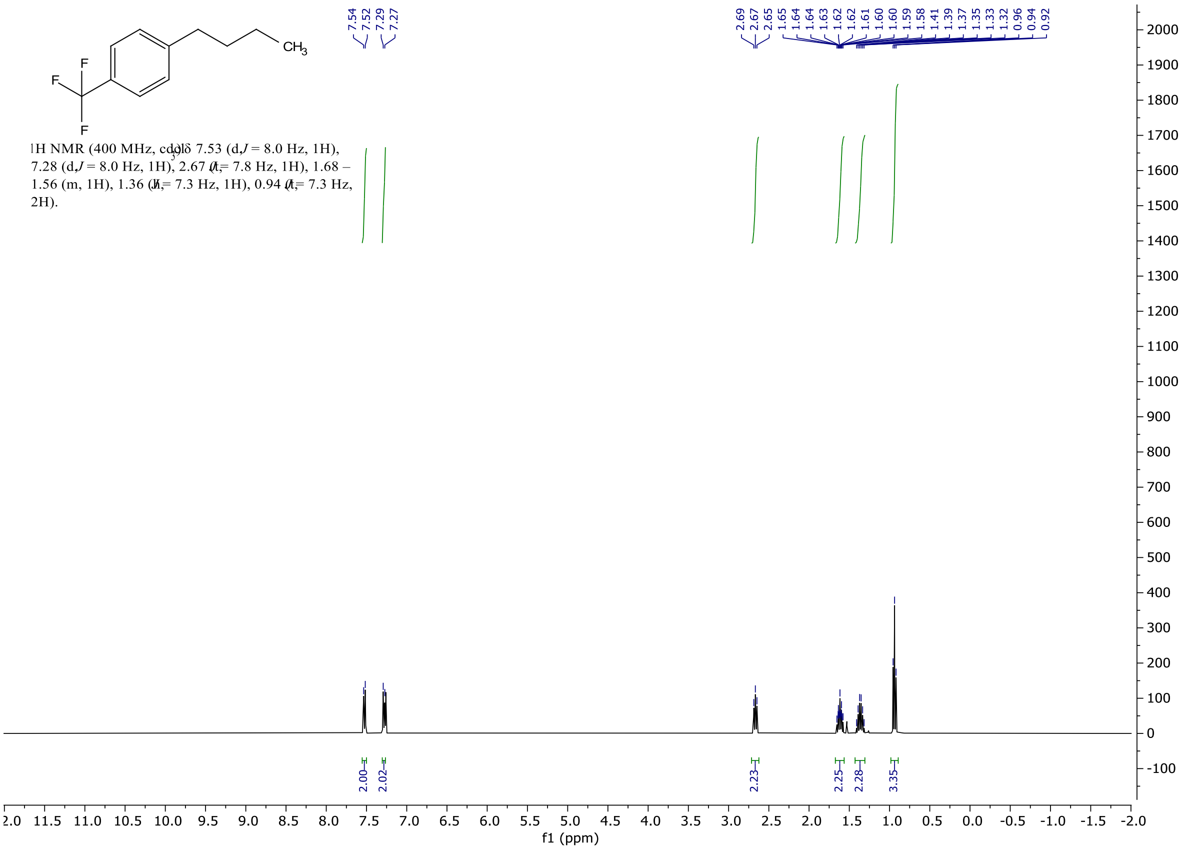
CH3
F
F
F
1H NMR (400 MHz, cdcl3) δ 7.53 (d, J = 8.0 Hz, 1H), 7.28 (d, J = 8.0 Hz, 1H), 2.67 (t, J = 7.8 Hz, 1H), 1.68 – 1.56 (m, 1H), 1.36 (h, J = 7.3 Hz, 1H), 0.94 (t, J = 7.3 Hz, 2H).
7.54
7.52
7.29
7.27
2.69
2.67
2.65
1.65
1.64
1.63
1.62
1.62
1.61
1.60
1.60
1.59
1.58
1.41
1.39
1.37
1.35
1.33
1.32
0.96
0.94
0.92
2.00
2.02
2.23
2.25
2.28
3.35
f1 (ppm)

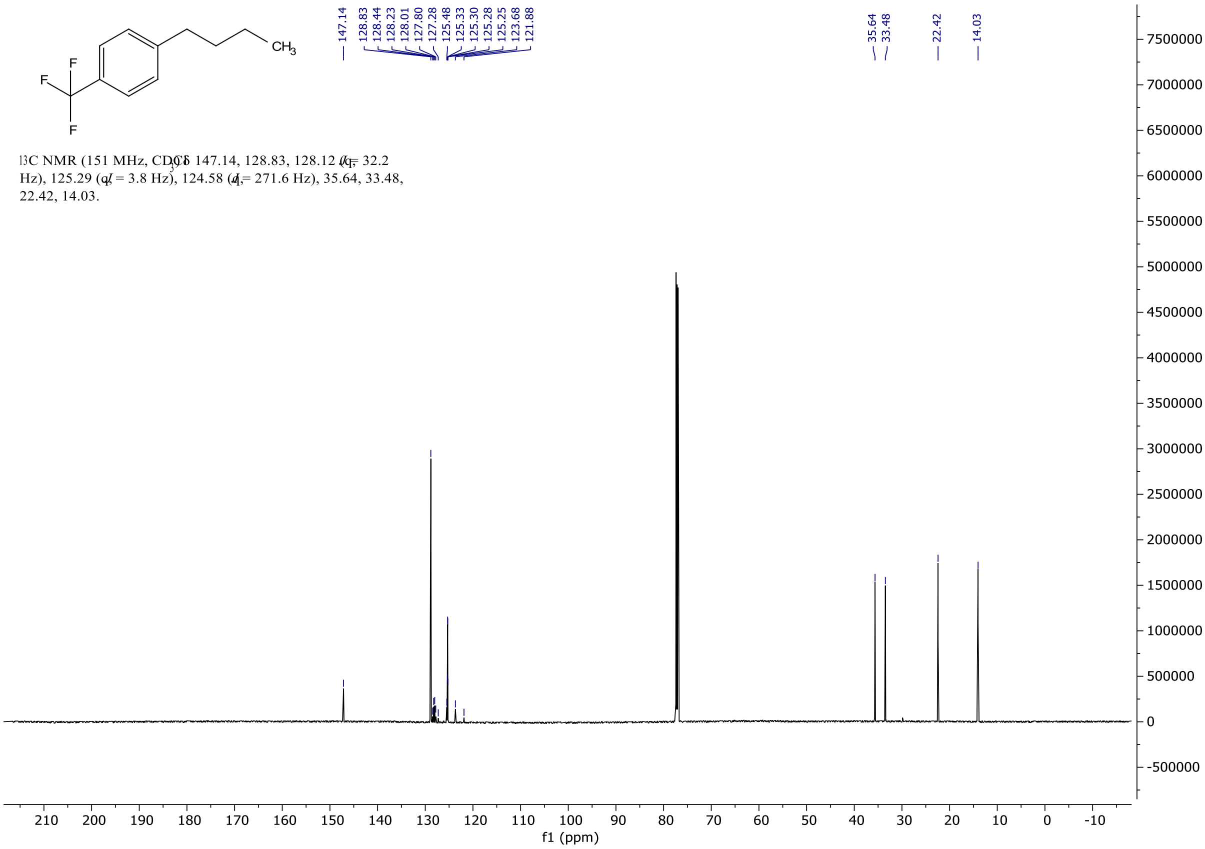
CH3
F
F
F
13C NMR (151 MHz, CDCl3) δ 147.14, 128.83, 128.12 (q, J = 32.2 Hz), 125.29 (q, J = 3.8 Hz), 124.58 (q, J = 271.6 Hz), 35.64, 33.48, 22.42, 14.03.
147.14
128.83
128.44
128.23
128.01
127.80
127.28
125.48
125.33
125.30
125.28
125.25
123.68
121.88
35.64
33.48
22.42
14.03
f1 (ppm)

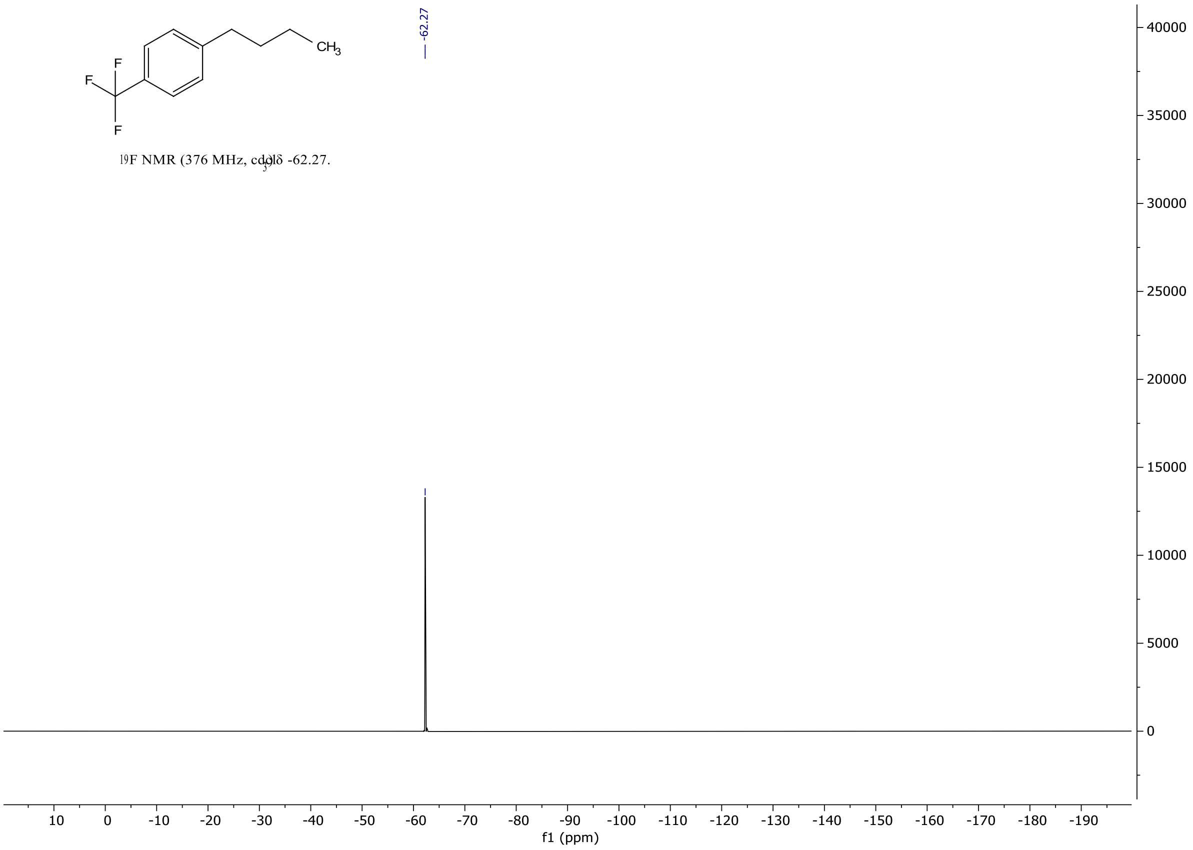

^{19}F NMR (376 MHz, $cdcl_3$) δ -62.27.

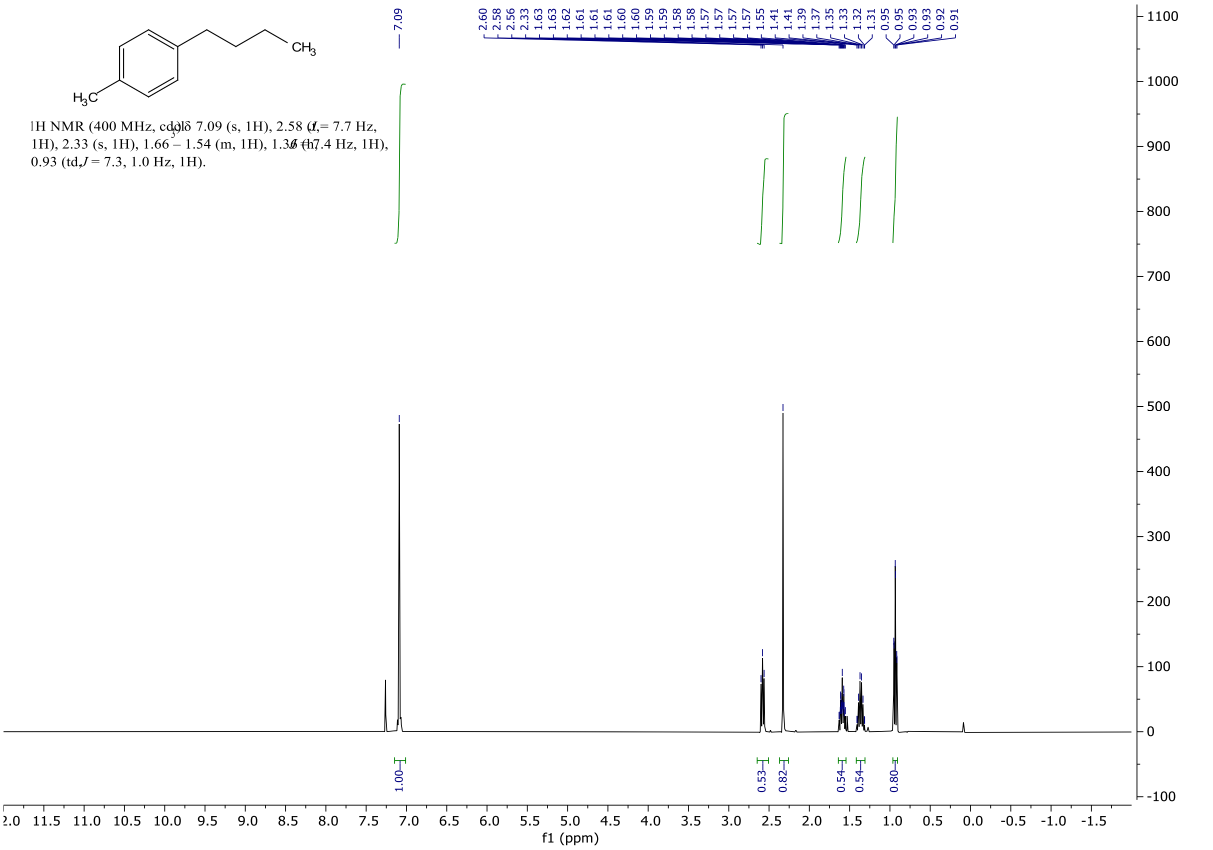

CH3
H3C
1H NMR (400 MHz, cdcl3) δ 7.09 (s, 1H), 2.58 (t, J = 7.7 Hz, 1H), 2.33 (s, 1H), 1.66 – 1.54 (m, 1H), 1.36 (h, J = 7.4 Hz, 1H), 0.93 (td, J = 7.3, 1.0 Hz, 1H).
f1 (ppm)

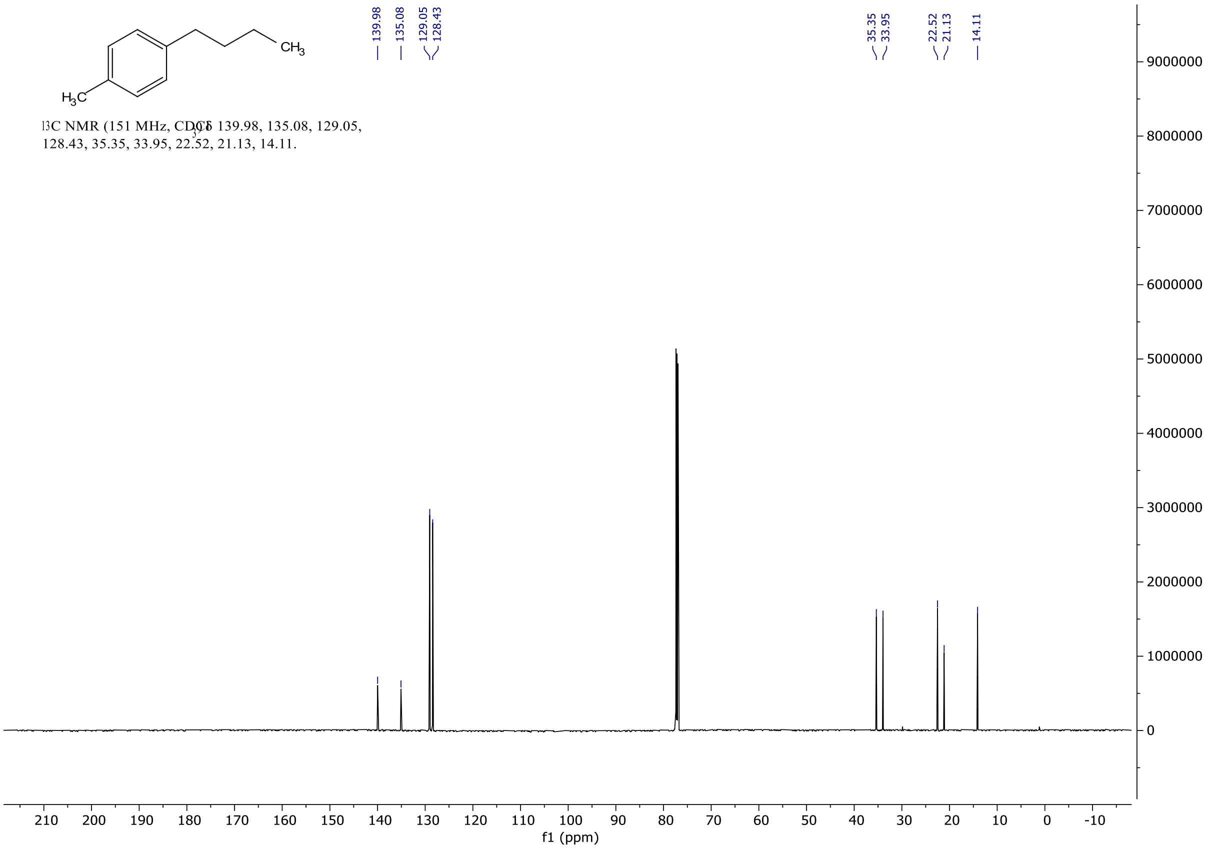

CH3
H3C
13C NMR (151 MHz, CDCl3) δ 139.98, 135.08, 129.05, 128.43, 35.35, 33.95, 22.52, 21.13, 14.11.
f1 (ppm)

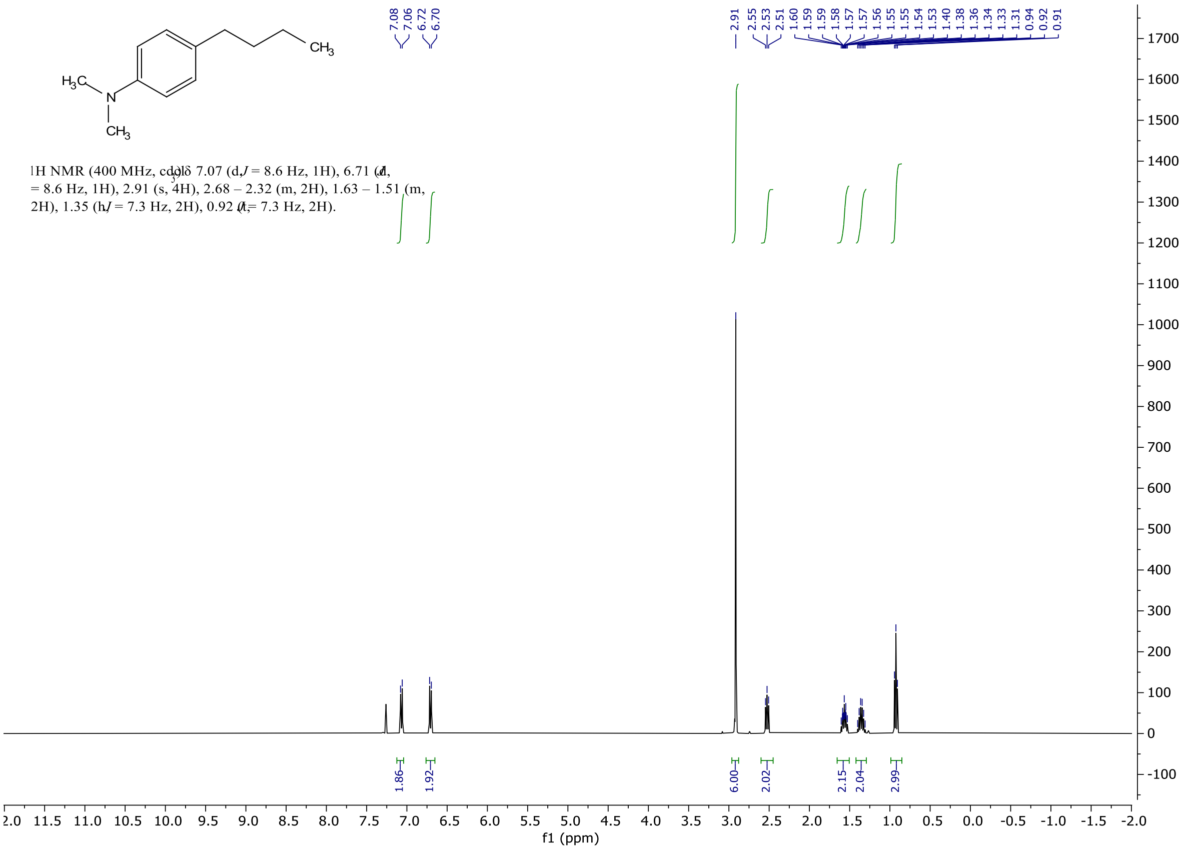
CH3
H3C
N
CH3
1H NMR (400 MHz, cdcl3) δ 7.07 (d, J = 8.6 Hz, 1H), 6.71 (d, J = 8.6 Hz, 1H), 2.91 (s, 4H), 2.68 – 2.32 (m, 2H), 1.63 – 1.51 (m, 2H), 1.35 (h, J = 7.3 Hz, 2H), 0.92 (t, J = 7.3 Hz, 2H).
f1 (ppm)

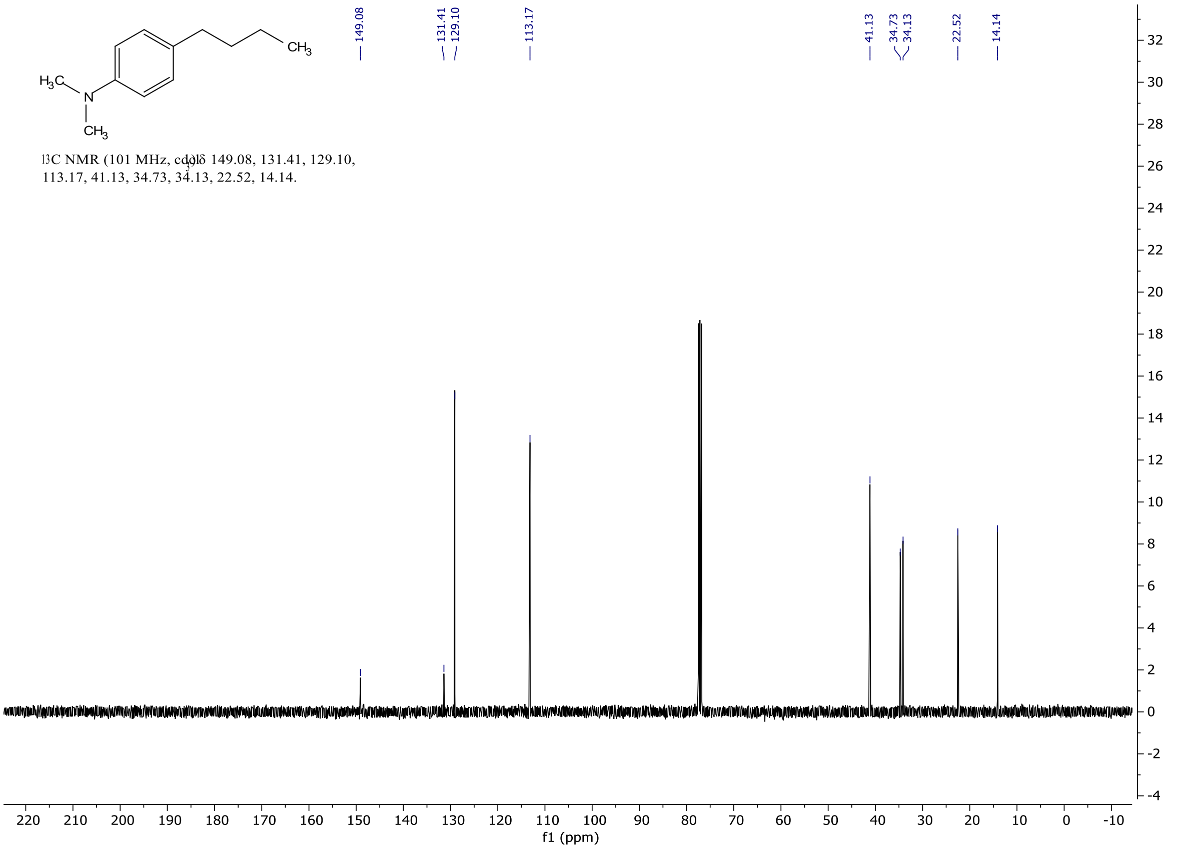
CH3
H3C
N
CH3
13C NMR (101 MHz, cdcl3) δ 149.08, 131.41, 129.10, 113.17, 41.13, 34.73, 34.13, 22.52, 14.14.
f1 (ppm)

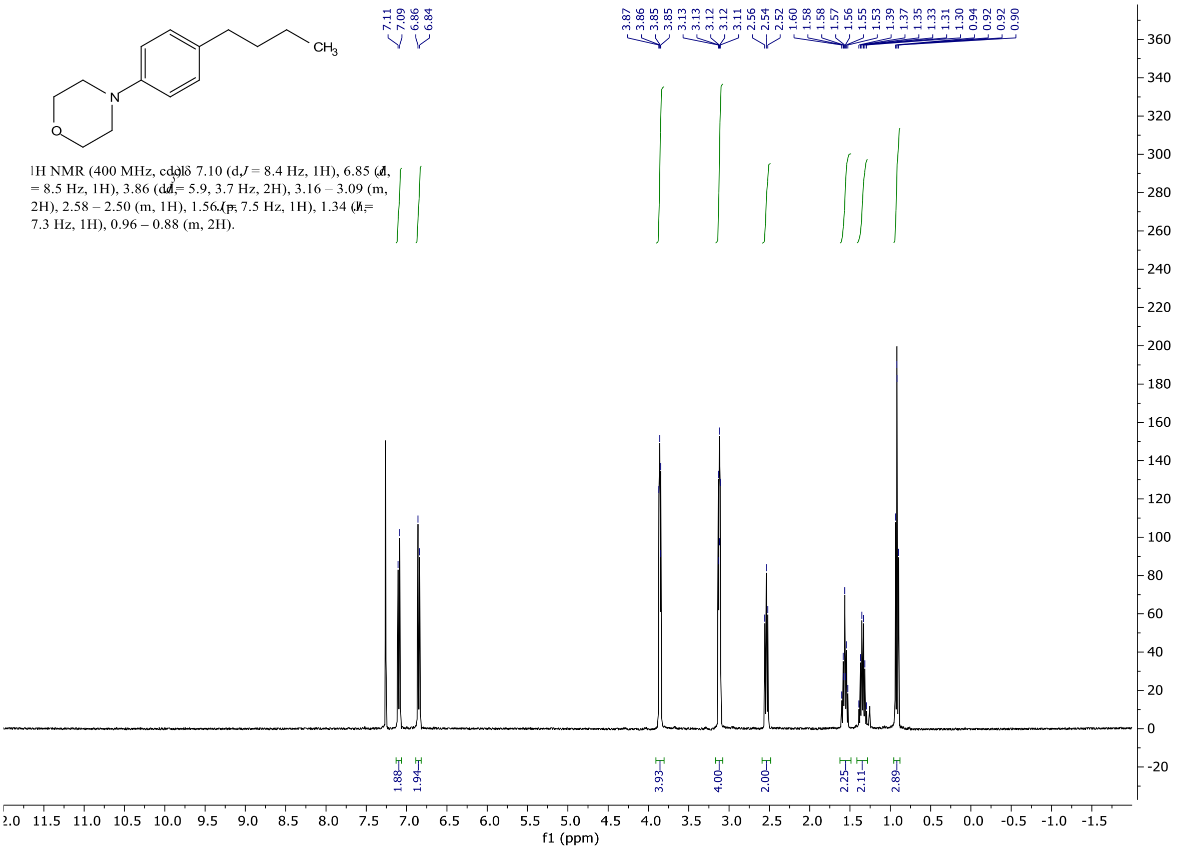
CH3
N
O
1H NMR (400 MHz, cdcl3) δ 7.10 (d, J = 8.4 Hz, 1H), 6.85 (d, J = 8.5 Hz, 1H), 3.86 (dd, J = 5.9, 3.7 Hz, 2H), 3.16 – 3.09 (m, 2H), 2.58 – 2.50 (m, 1H), 1.56 (p, J = 7.5 Hz, 1H), 1.34 (h, J = 7.3 Hz, 1H), 0.96 – 0.88 (m, 2H).
f1 (ppm)

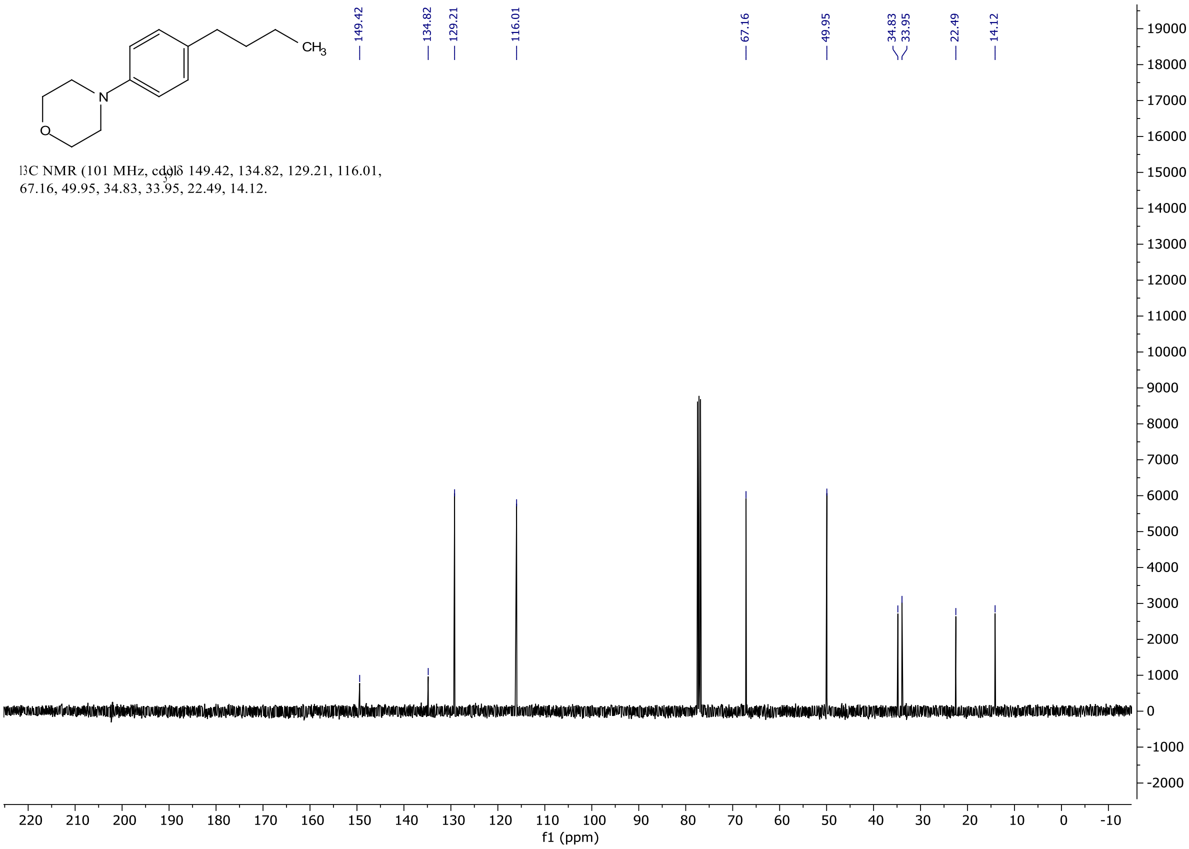
CH3
N
O
13C NMR (101 MHz, cdcl3) δ 149.42, 134.82, 129.21, 116.01, 67.16, 49.95, 34.83, 33.95, 22.49, 14.12.
f1 (ppm)

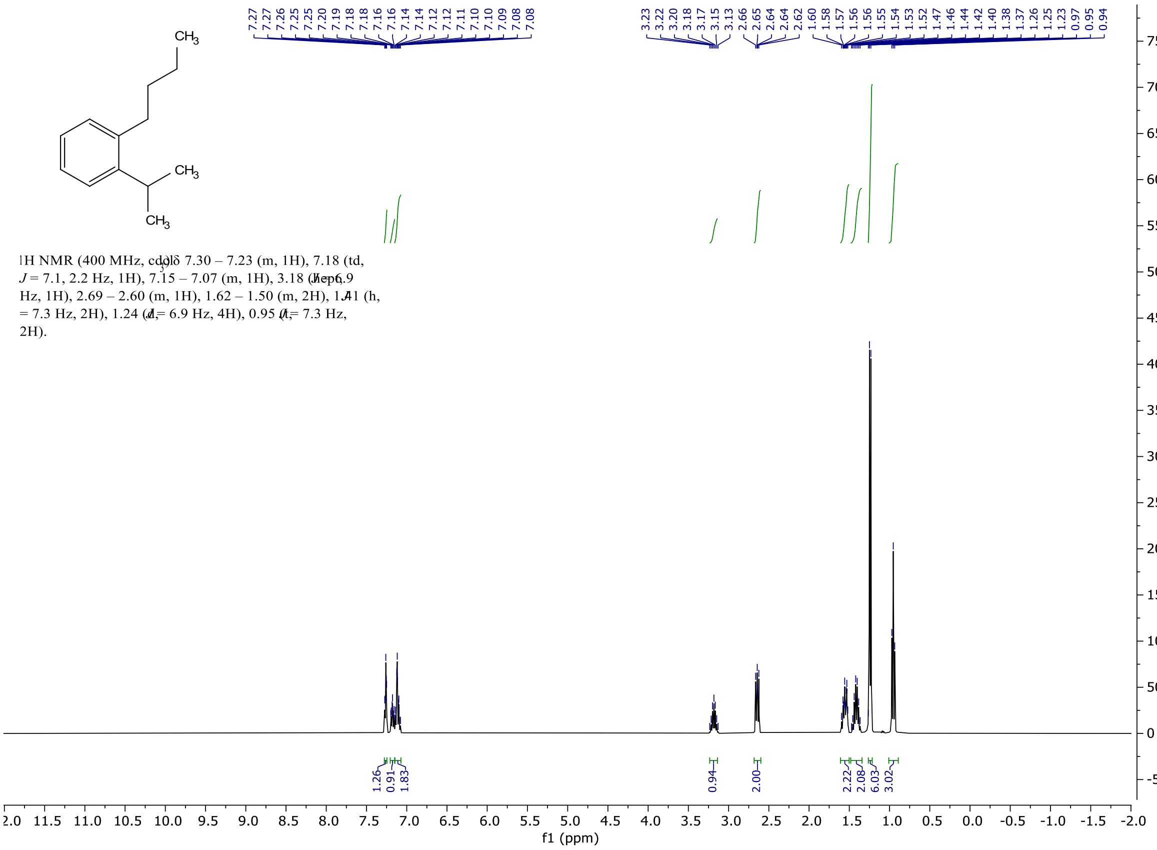

^{1}H NMR (400 MHz, cdcl$_3$) δ 7.30 – 7.23 (m, 1H), 7.18 (td, J = 7.1, 2.2 Hz, 1H), 7.15 – 7.07 (m, 1H), 3.18 (hept, J = 6.9 Hz, 1H), 2.69 – 2.60 (m, 1H), 1.62 – 1.50 (m, 2H), 1.41 (h, J = 7.3 Hz, 2H), 1.24 (d, J = 6.9 Hz, 4H), 0.95 (t, J = 7.3 Hz, 2H).

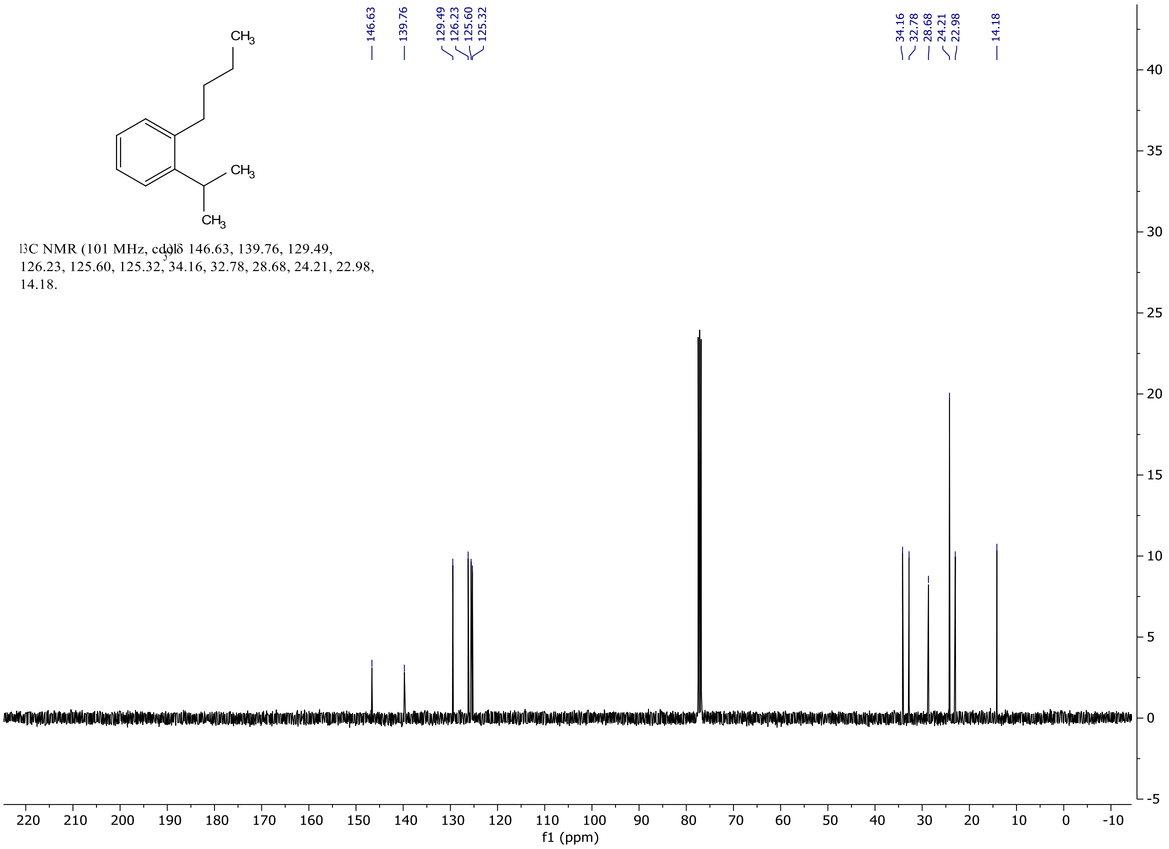

^{13}C NMR (101 MHz, cdcl$_3$) δ 146.63, 139.76, 129.49, 126.23, 125.60, 125.32, 34.16, 32.78, 28.68, 24.21, 22.98, 14.18.

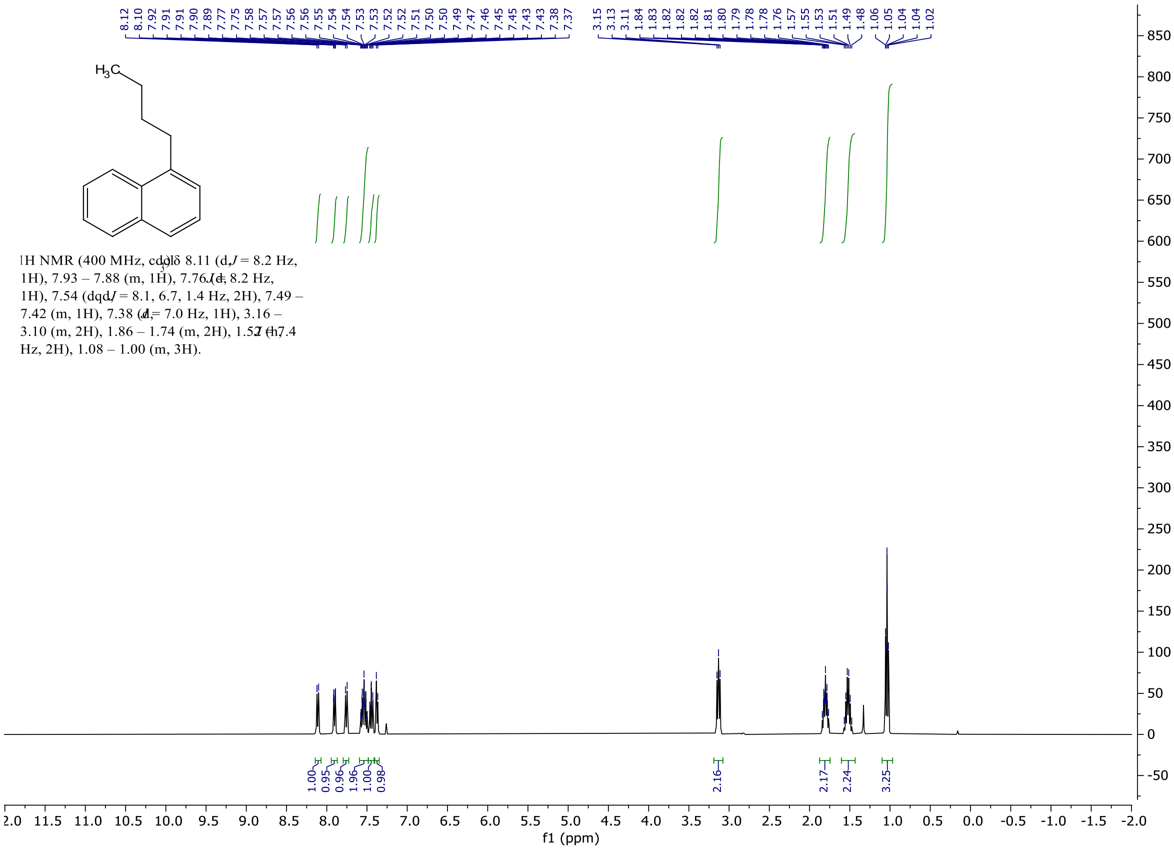

1H NMR (400 MHz, $cdcl_3$) δ 8.11 (d, J = 8.2 Hz, 1H), 7.93 – 7.88 (m, 1H), 7.76 (d, J = 8.2 Hz, 1H), 7.54 (dqd, J = 8.1, 6.7, 1.4 Hz, 2H), 7.49 – 7.42 (m, 1H), 7.38 (d, J = 7.0 Hz, 1H), 3.16 – 3.10 (m, 2H), 1.86 – 1.74 (m, 2H), 1.52 (h, J = 7.4 Hz, 2H), 1.08 – 1.00 (m, 3H).

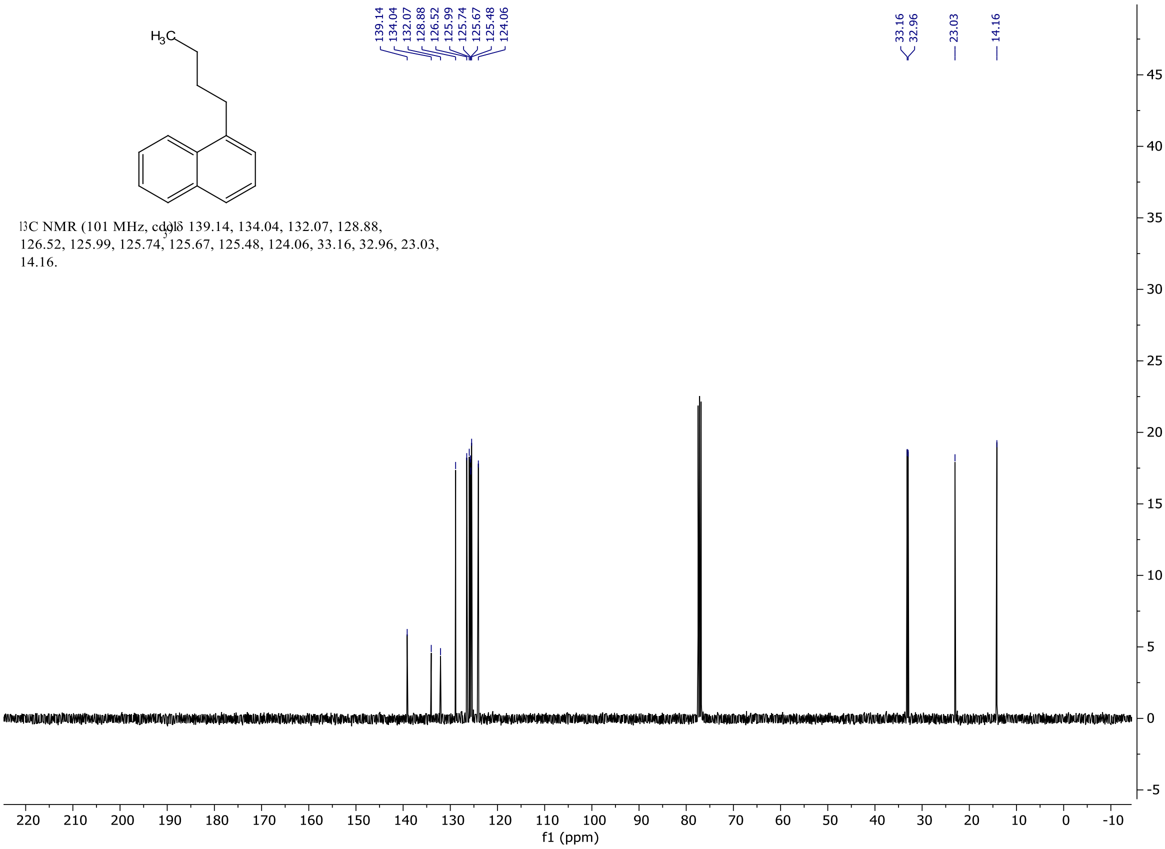

13C NMR (101 MHz, $cdcl_3$) δ 139.14, 134.04, 132.07, 128.88, 126.52, 125.99, 125.74, 125.67, 125.48, 124.06, 33.16, 32.96, 23.03, 14.16.

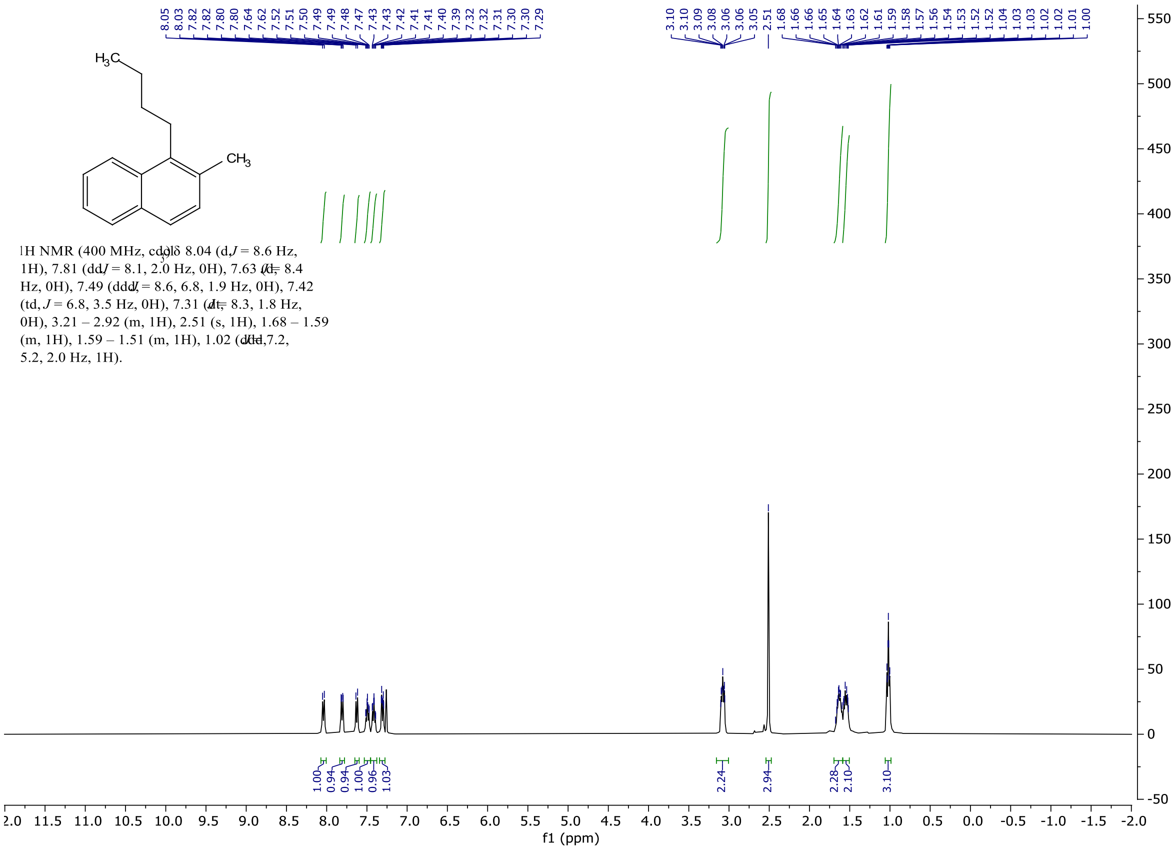

1H NMR (400 MHz, $cdcl_3$) δ 8.04 (d, J = 8.6 Hz, 1H), 7.81 (dd, J = 8.1, 2.0 Hz, 0H), 7.63 (d, J = 8.4 Hz, 0H), 7.49 (ddd, J = 8.6, 6.8, 1.9 Hz, 0H), 7.42 (td, J = 6.8, 3.5 Hz, 0H), 7.31 (dt, J = 8.3, 1.8 Hz, 0H), 3.21 – 2.92 (m, 1H), 2.51 (s, 1H), 1.68 – 1.59 (m, 1H), 1.59 – 1.51 (m, 1H), 1.02 (ddd, J = 7.2, 5.2, 2.0 Hz, 1H).

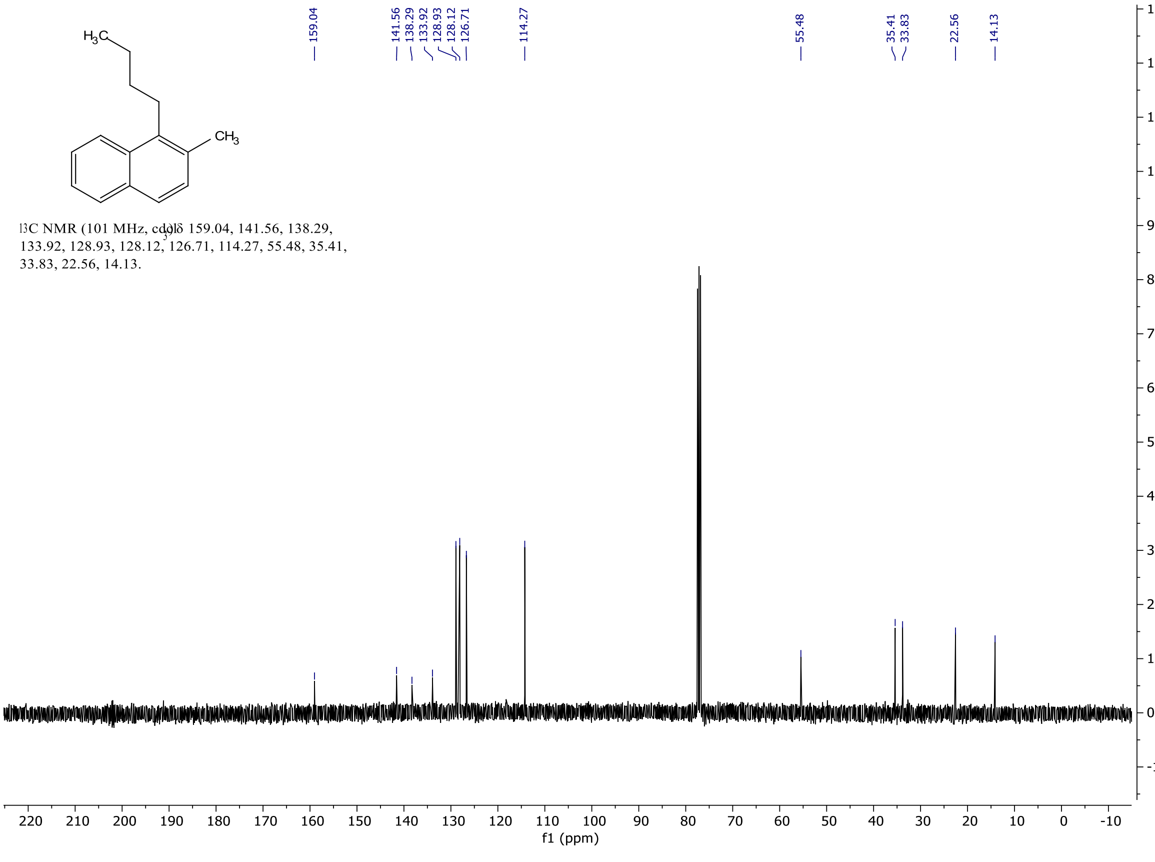

13C NMR (101 MHz, $cdcl_3$) δ 159.04, 141.56, 138.29, 133.92, 128.93, 128.12, 126.71, 114.27, 55.48, 35.41, 33.83, 22.56, 14.13.

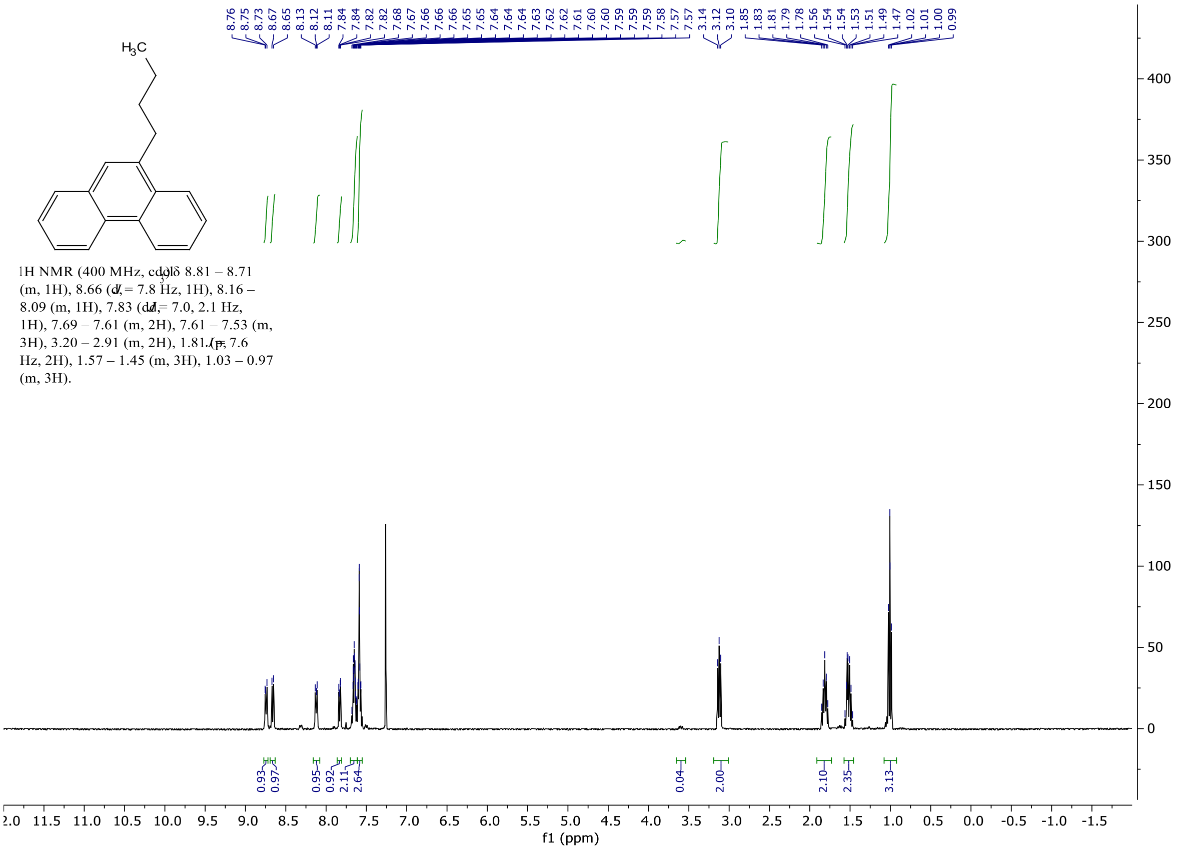

1H NMR (400 MHz, cdcl3) δ 8.81 – 8.71 (m, 1H), 8.66 (d, J = 7.8 Hz, 1H), 8.16 – 8.09 (m, 1H), 7.83 (dd, J = 7.0, 2.1 Hz, 1H), 7.69 – 7.61 (m, 2H), 7.61 – 7.53 (m, 3H), 3.20 – 2.91 (m, 2H), 1.81 (p, J = 7.6 Hz, 2H), 1.57 – 1.45 (m, 3H), 1.03 – 0.97 (m, 3H).

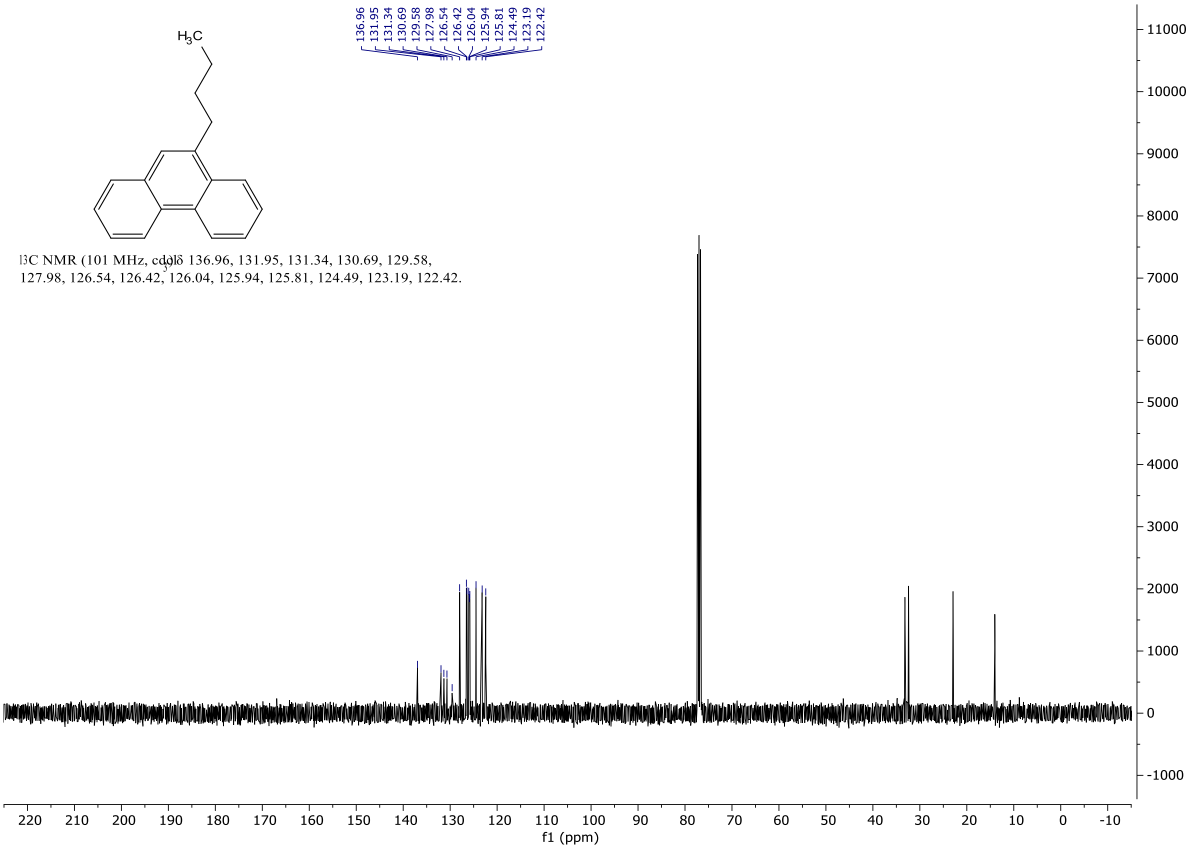

13C NMR (101 MHz, cdcl3) δ 136.96, 131.95, 131.34, 130.69, 129.58, 127.98, 126.54, 126.42, 126.04, 125.94, 125.81, 124.49, 123.19, 122.42.

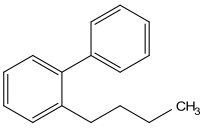

1H NMR (400 MHz, cdcl3) δ 7.44 – 7.38 (m, 1H), 7.37 – 7.34 (m, 0H), 7.34 – 7.28 (m, 1H), 7.25 – 7.19 (m, 1H), 2.63 – 2.54 (m, 1H), 1.50 – 1.41 (m, 1H), 1.22 (h, J = 7.3 Hz, 1H), 0.80 (t, J = 7.3 Hz, 1H).

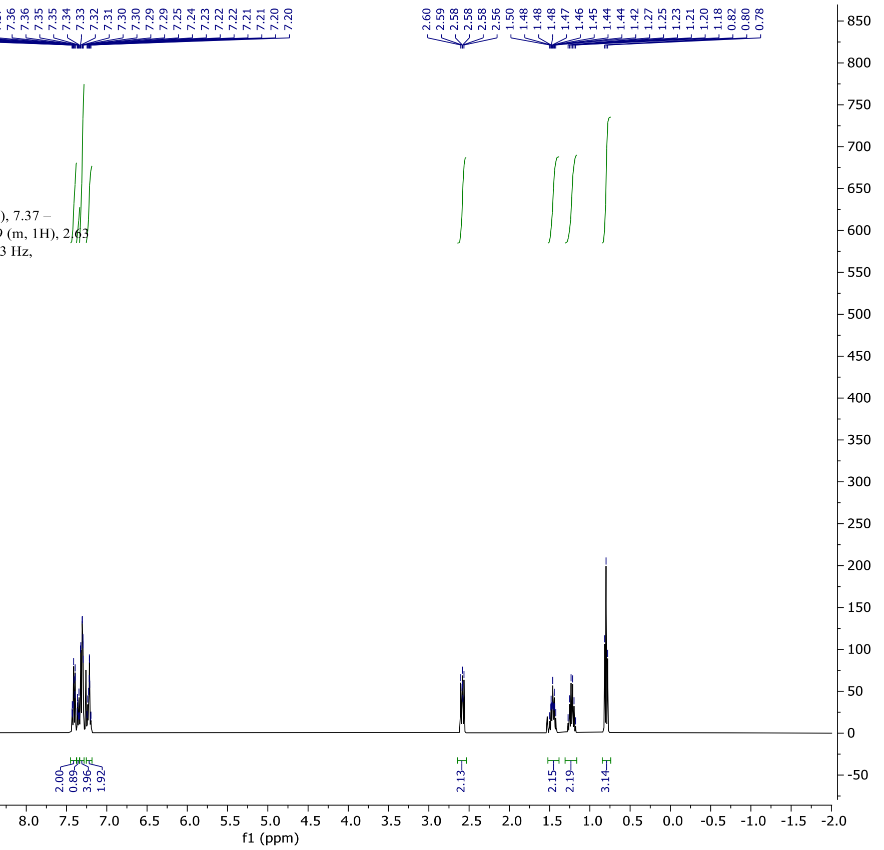

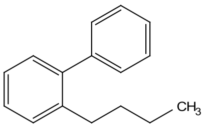

13C NMR (101 MHz, cdcl3) δ 142.19, 141.98, 140.48, 130.15, 129.40, 129.36, 128.10, 127.43, 126.83, 125.64, 33.71, 32.82, 22.64, 13.97.

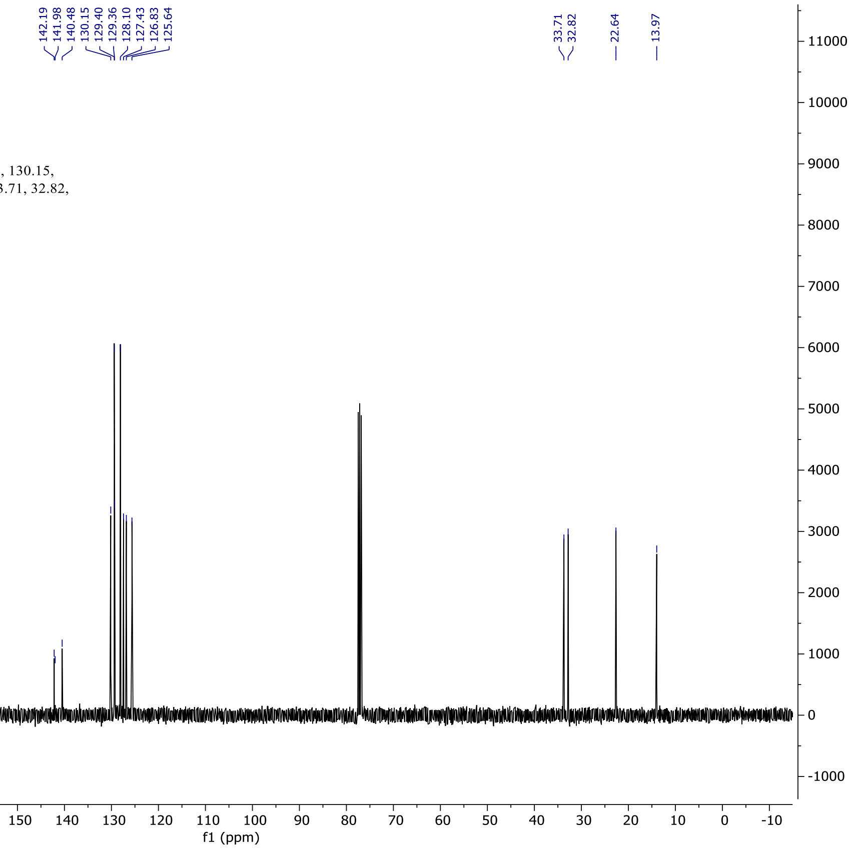

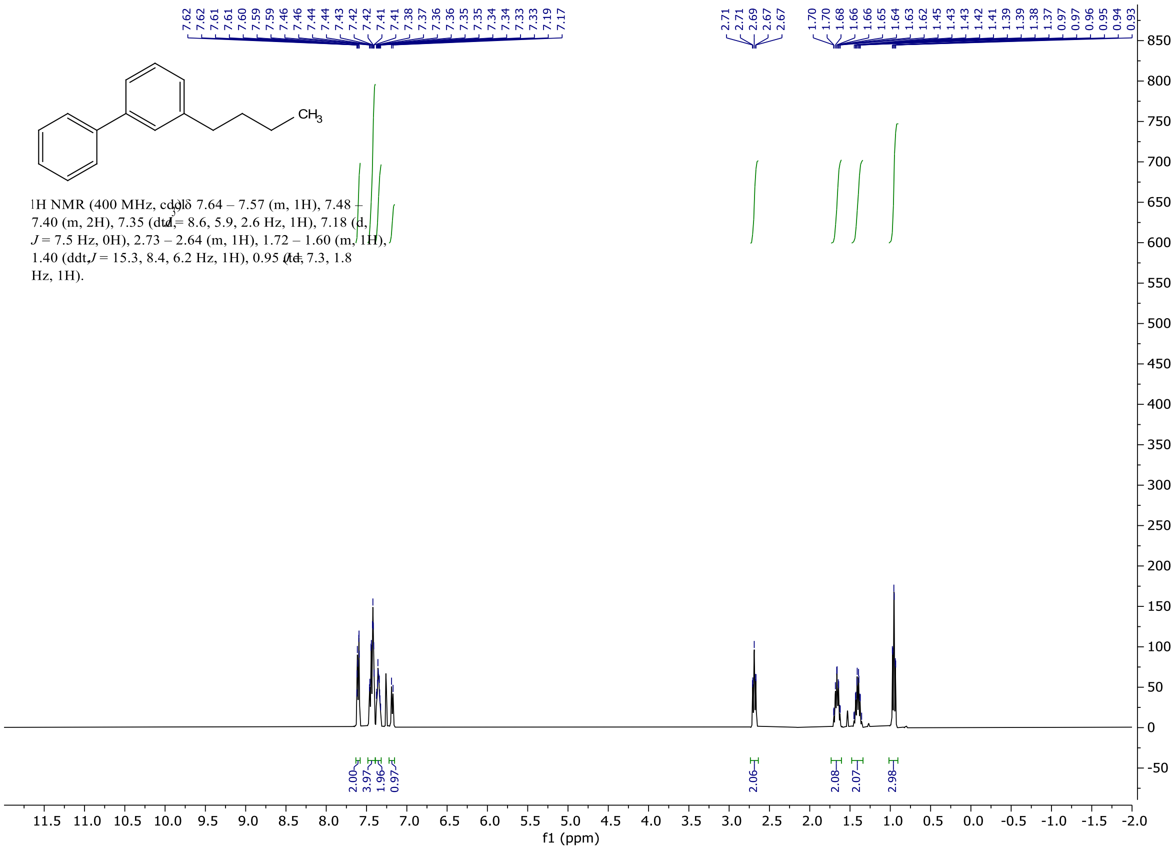

1H NMR (400 MHz, cdcl3) δ 7.64 – 7.57 (m, 1H), 7.48 – 7.40 (m, 2H), 7.35 (dtd, *J* = 8.6, 5.9, 2.6 Hz, 1H), 7.18 (d, *J* = 7.5 Hz, 0H), 2.73 – 2.64 (m, 1H), 1.72 – 1.60 (m, 1H), 1.40 (ddt, *J* = 15.3, 8.4, 6.2 Hz, 1H), 0.95 (td, *J* = 7.3, 1.8 Hz, 1H).

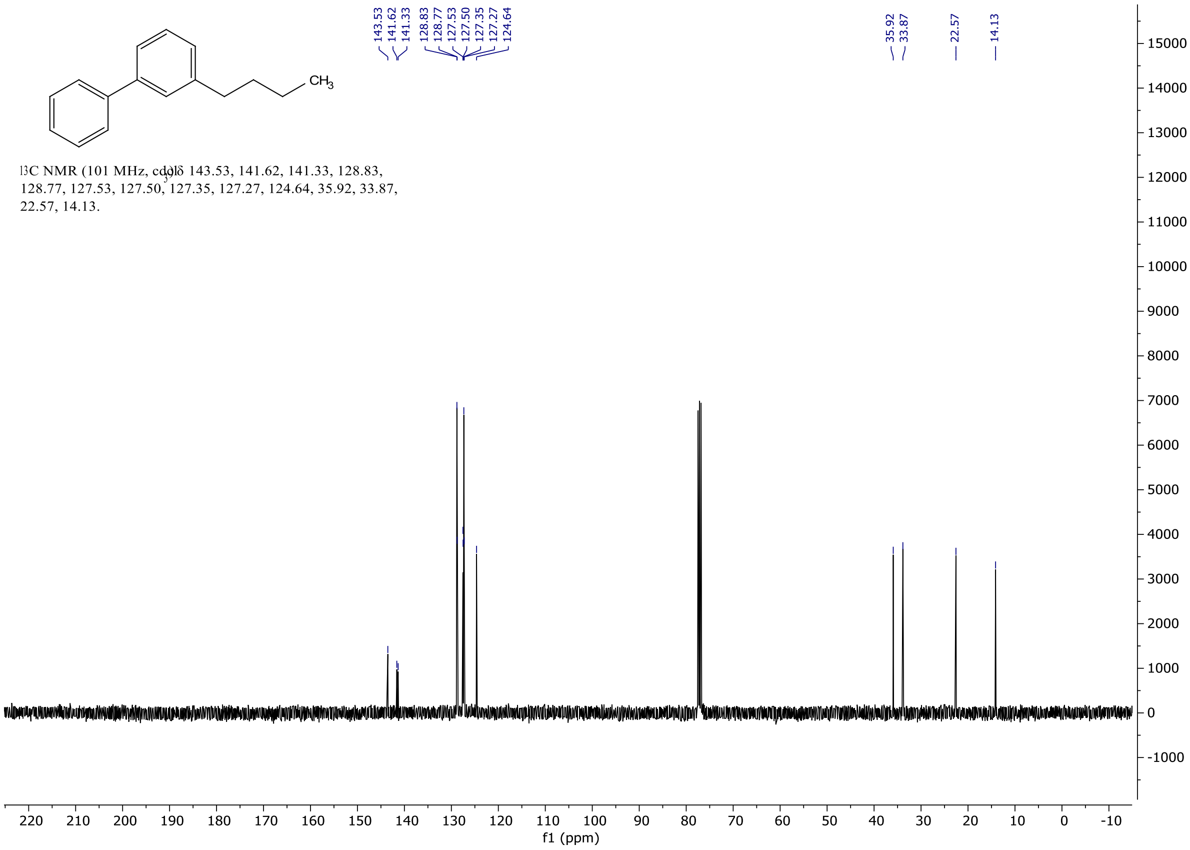

13C NMR (101 MHz, cdcl3) δ 143.53, 141.62, 141.33, 128.83, 128.77, 127.53, 127.50, 127.35, 127.27, 124.64, 35.92, 33.87, 22.57, 14.13.

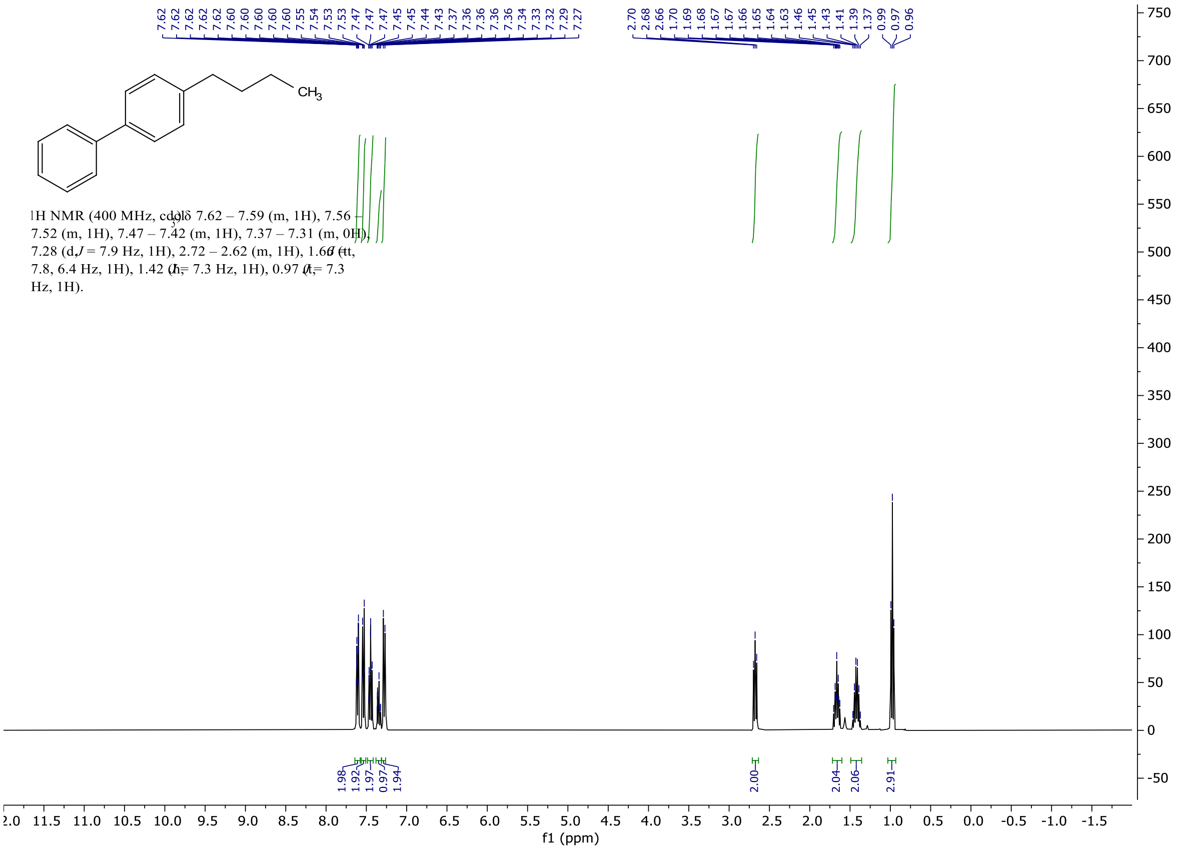
CH3
1H NMR (400 MHz, cdcl3) δ 7.62 – 7.59 (m, 1H), 7.56 – 7.52 (m, 1H), 7.47 – 7.42 (m, 1H), 7.37 – 7.31 (m, 0H), 7.28 (d, J = 7.9 Hz, 1H), 2.72 – 2.62 (m, 1H), 1.66 (tt, J = 7.8, 6.4 Hz, 1H), 1.42 (h, J = 7.3 Hz, 1H), 0.97 (t, J = 7.3 Hz, 1H).
f1 (ppm)

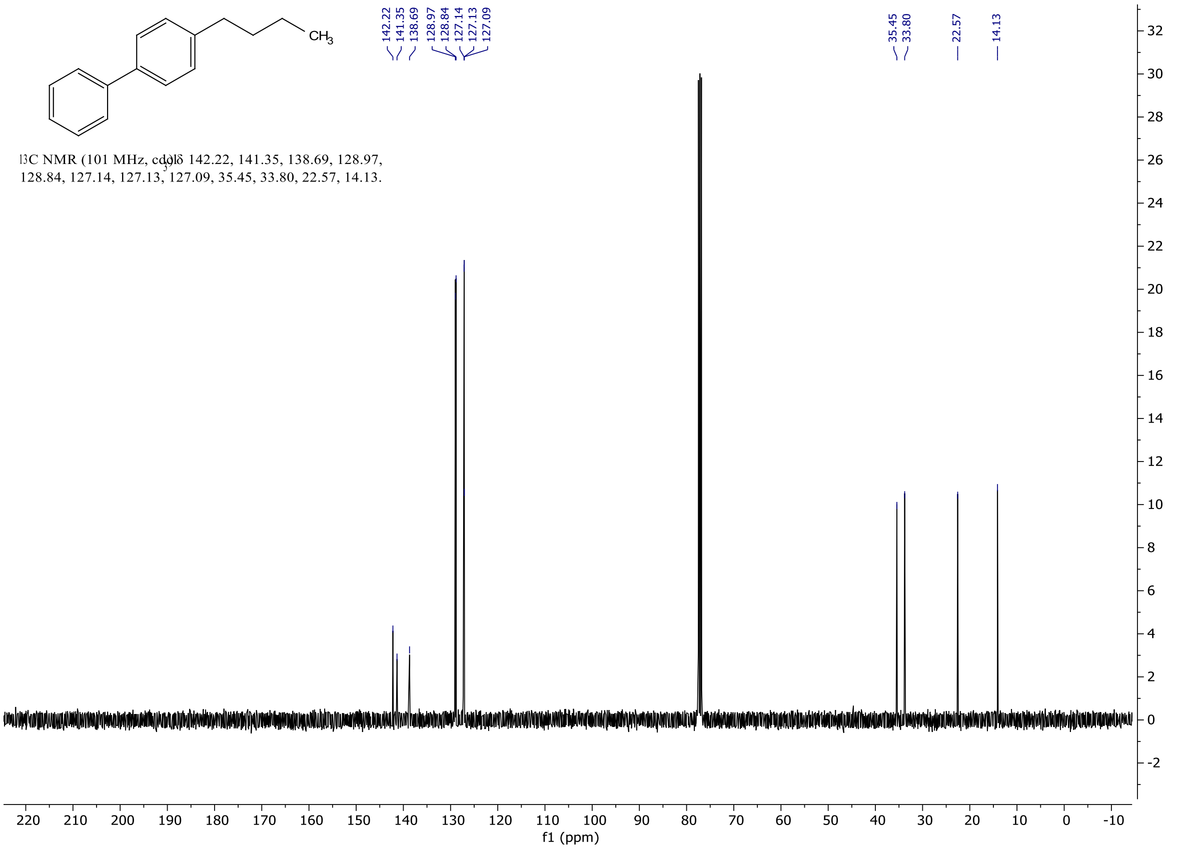
CH3
13C NMR (101 MHz, cdcl3) δ 142.22, 141.35, 138.69, 128.97, 128.84, 127.14, 127.13, 127.09, 35.45, 33.80, 22.57, 14.13.
f1 (ppm)

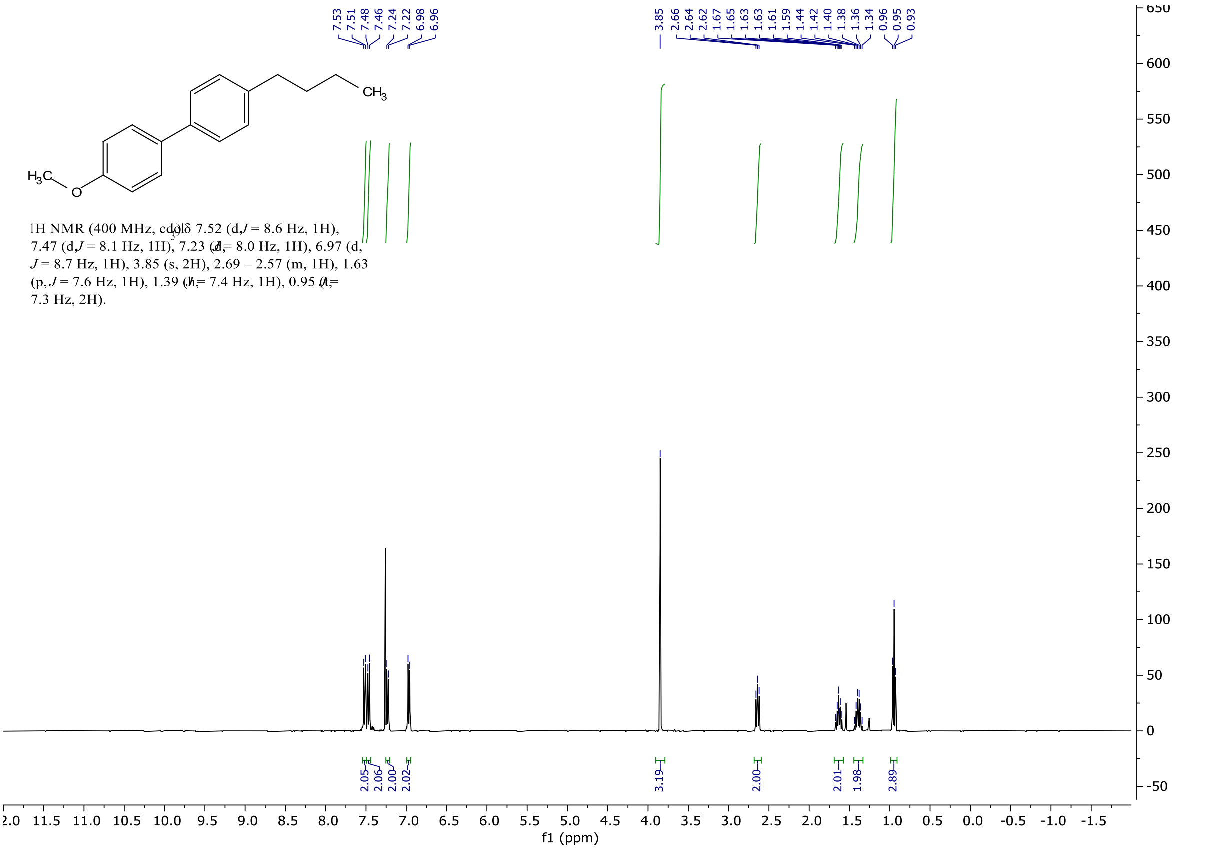
CH3
H3C
O
1H NMR (400 MHz, cdcl3) δ 7.52 (d, J = 8.6 Hz, 1H), 7.47 (d, J = 8.1 Hz, 1H), 7.23 (d, J = 8.0 Hz, 1H), 6.97 (d, J = 8.7 Hz, 1H), 3.85 (s, 2H), 2.69 – 2.57 (m, 1H), 1.63 (p, J = 7.6 Hz, 1H), 1.39 (h, J = 7.4 Hz, 1H), 0.95 (t, J = 7.3 Hz, 2H).
f1 (ppm)

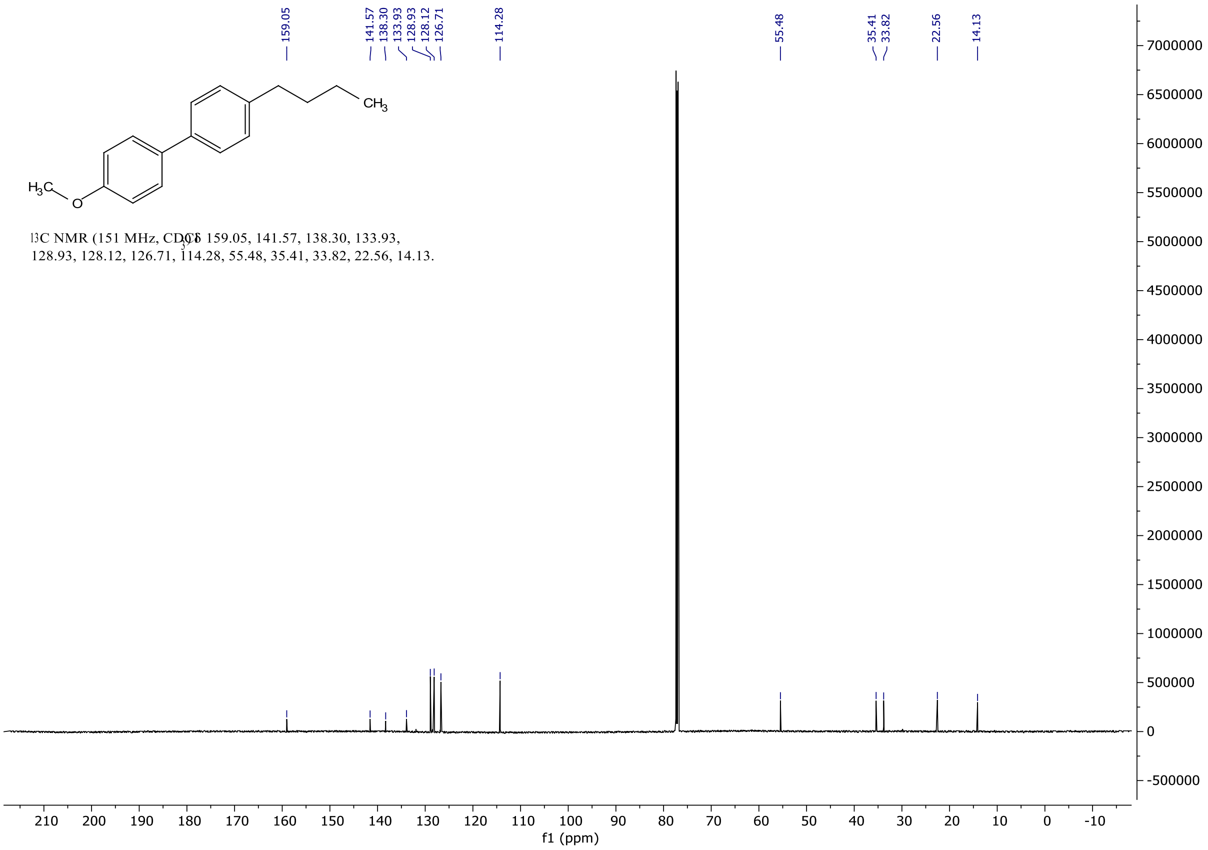
CH3
H3C
O
13C NMR (151 MHz, CDCl3) δ 159.05, 141.57, 138.30, 133.93, 128.93, 128.12, 126.71, 114.28, 55.48, 35.41, 33.82, 22.56, 14.13.
f1 (ppm)

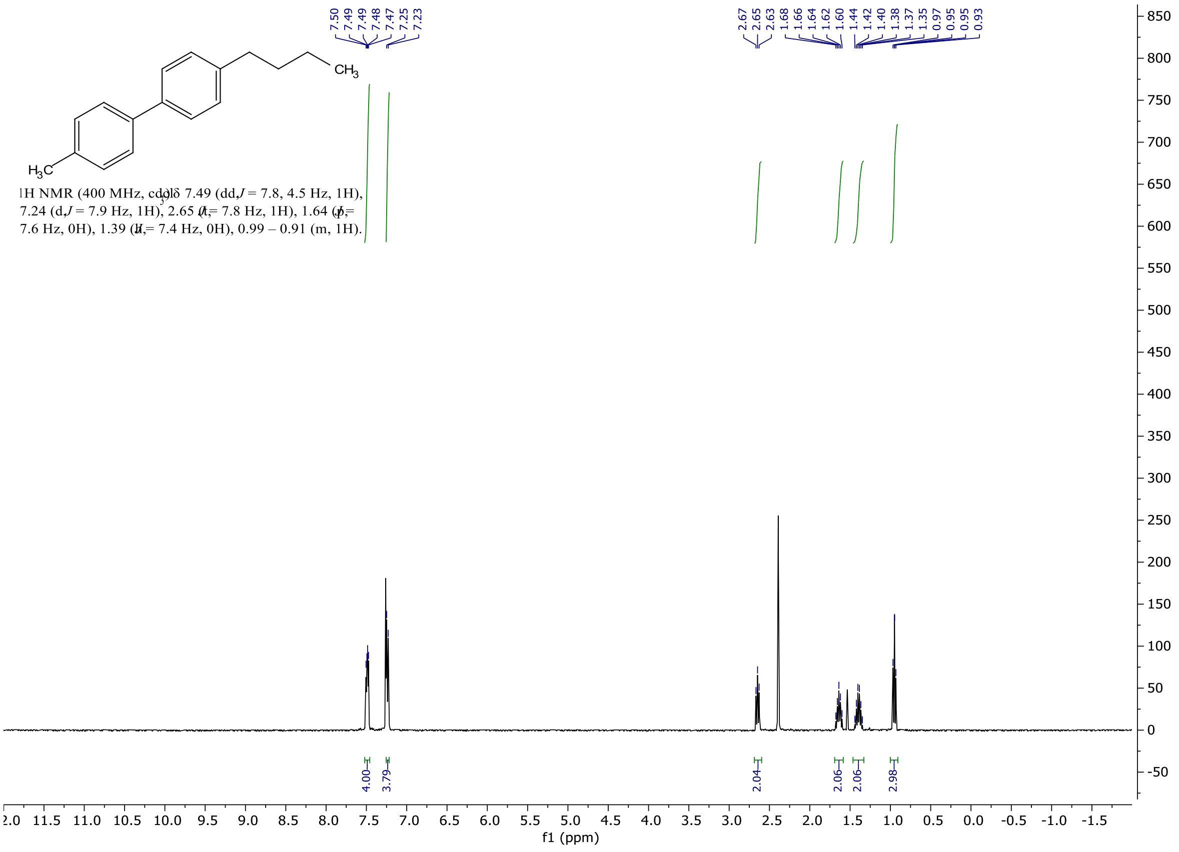
CH3
H3C
1H NMR (400 MHz, cdcl3) δ 7.49 (dd, J = 7.8, 4.5 Hz, 1H),
7.24 (d, J = 7.9 Hz, 1H), 2.65 (t, J = 7.8 Hz, 1H), 1.64 (p, J =
7.6 Hz, 0H), 1.39 (h, J = 7.4 Hz, 0H), 0.99 – 0.91 (m, 1H).
7.50 7.49 7.49 7.48 7.47 7.25 7.23
2.67 2.65 2.63 1.68 1.66 1.64 1.62 1.60 1.44 1.42 1.40 1.38 1.37 1.35 0.97 0.95 0.95 0.93
4.00 3.79 2.04 2.06 2.06 2.98
f1 (ppm)

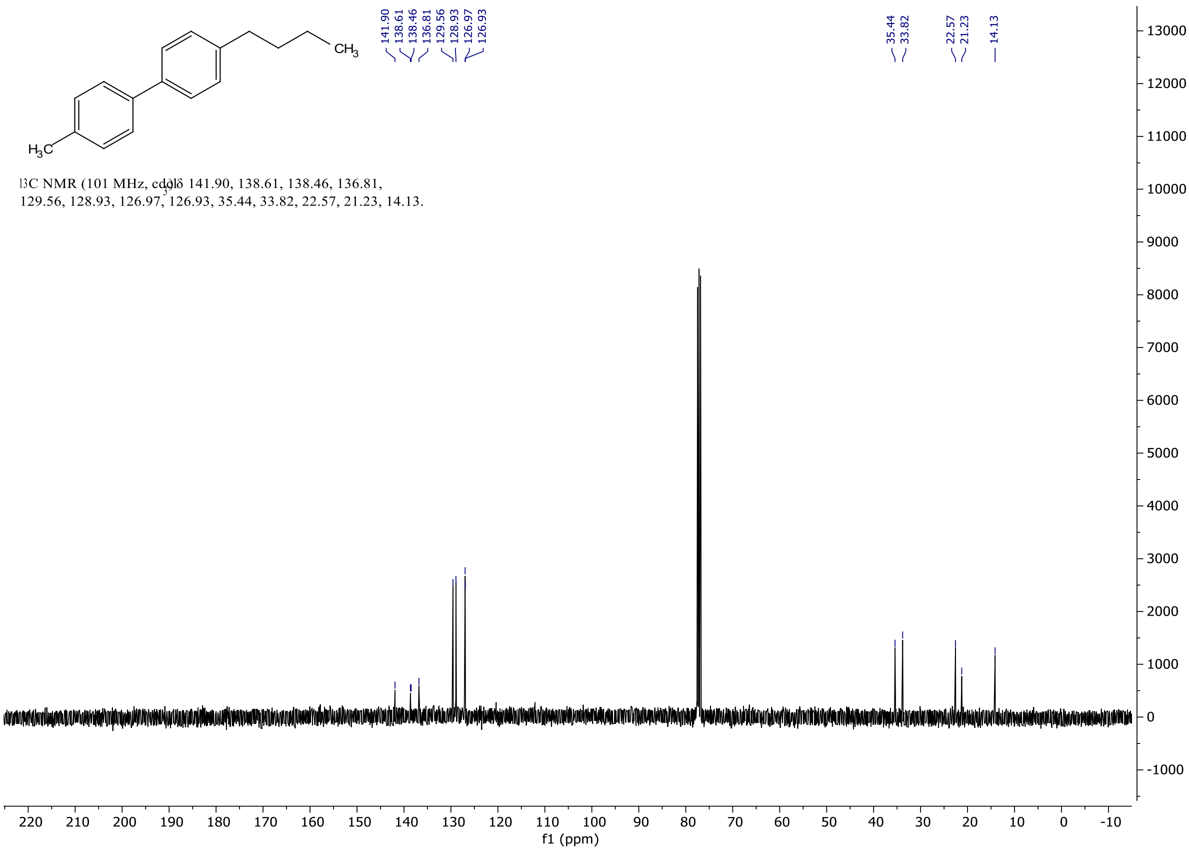
CH3
H3C
13C NMR (101 MHz, cdcl3) δ 141.90, 138.61, 138.46, 136.81,
129.56, 128.93, 126.97, 126.93, 35.44, 33.82, 22.57, 21.23, 14.13.
141.90 138.61 138.46 136.81 129.56 128.93 126.97 126.93
35.44 33.82 22.57 21.23 14.13
f1 (ppm)

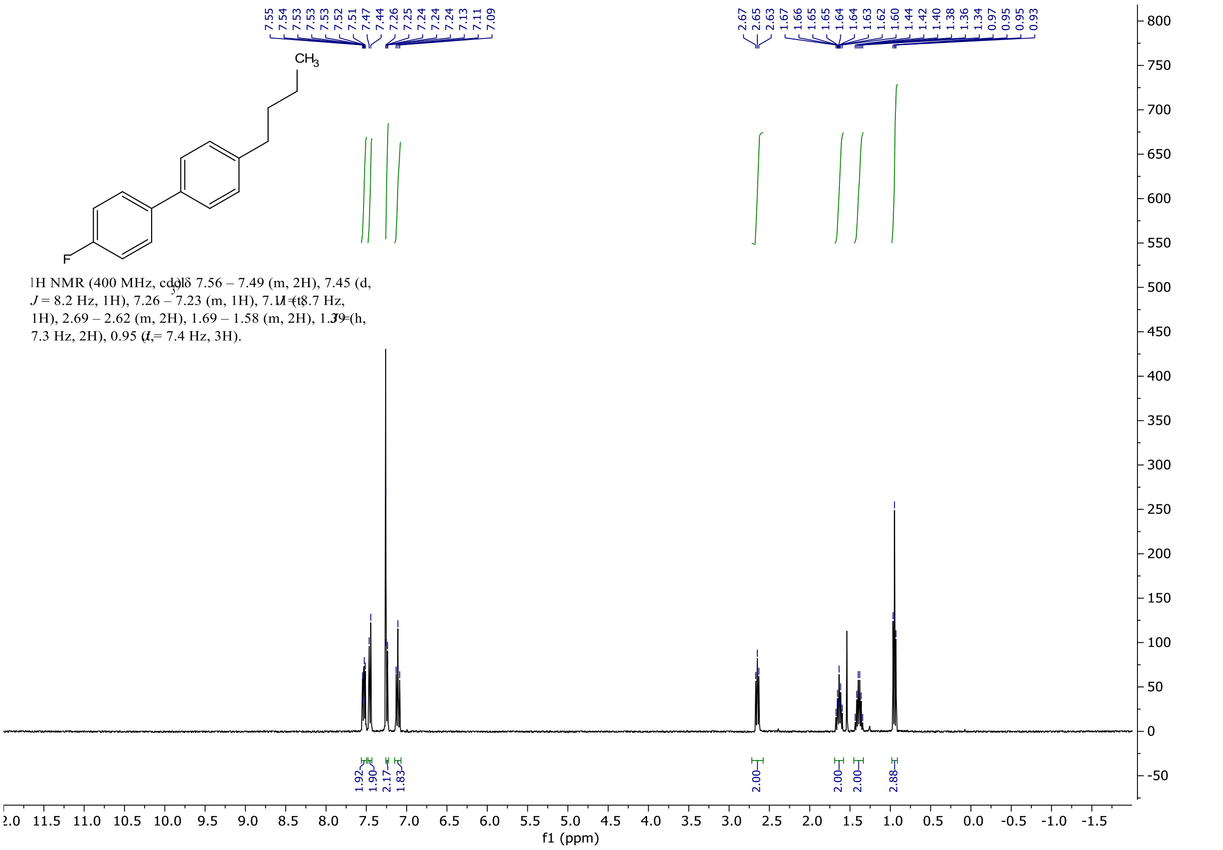
CH3
F
1H NMR (400 MHz, cdcl3) δ 7.56 – 7.49 (m, 2H), 7.45 (d, J = 8.2 Hz, 1H), 7.26 – 7.23 (m, 1H), 7.11 (t, J = 8.7 Hz, 1H), 2.69 – 2.62 (m, 2H), 1.69 – 1.58 (m, 2H), 1.39 (h, J = 7.3 Hz, 2H), 0.95 (t, J = 7.4 Hz, 3H).
f1 (ppm)

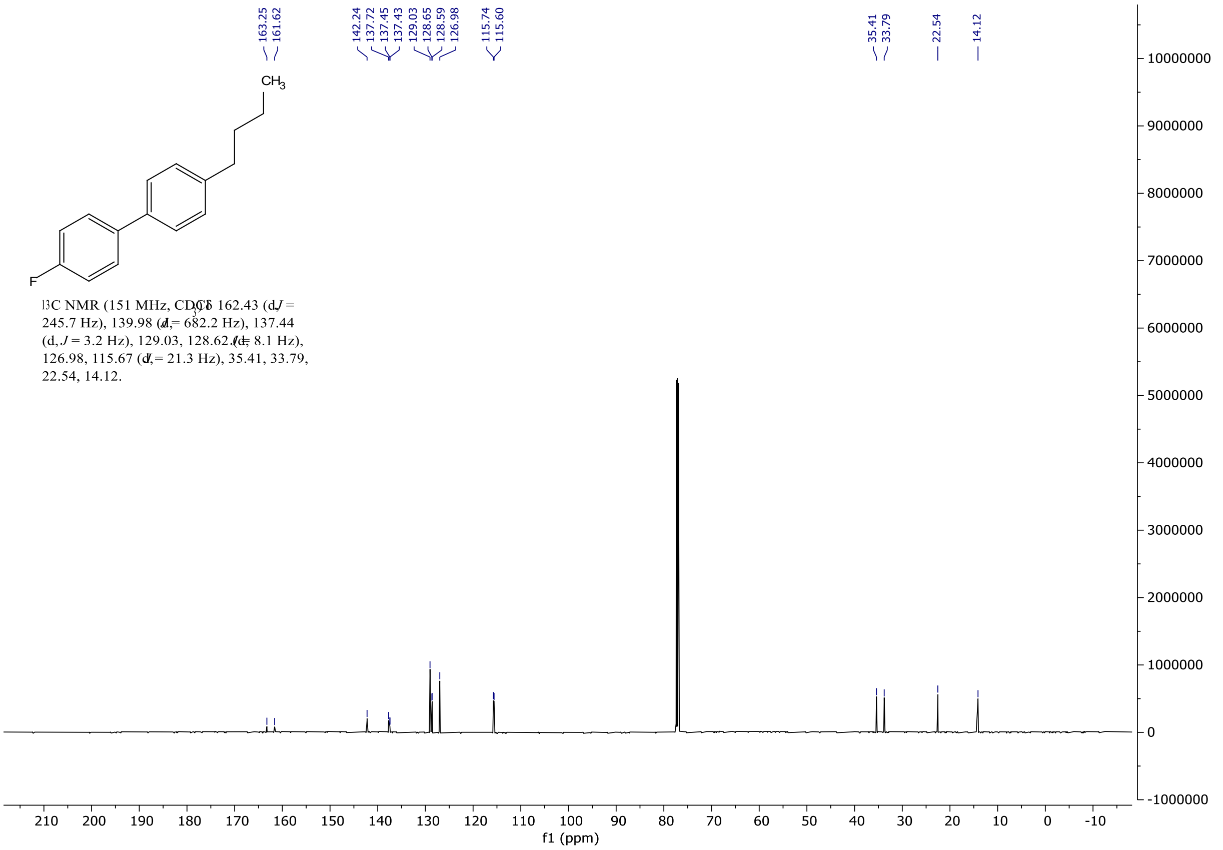
CH3
F
13C NMR (151 MHz, CDCl3) δ 162.43 (d, J = 245.7 Hz), 139.98 (d, J = 682.2 Hz), 137.44 (d, J = 3.2 Hz), 129.03, 128.62 (d, J = 8.1 Hz), 126.98, 115.67 (d, J = 21.3 Hz), 35.41, 33.79, 22.54, 14.12.
f1 (ppm)

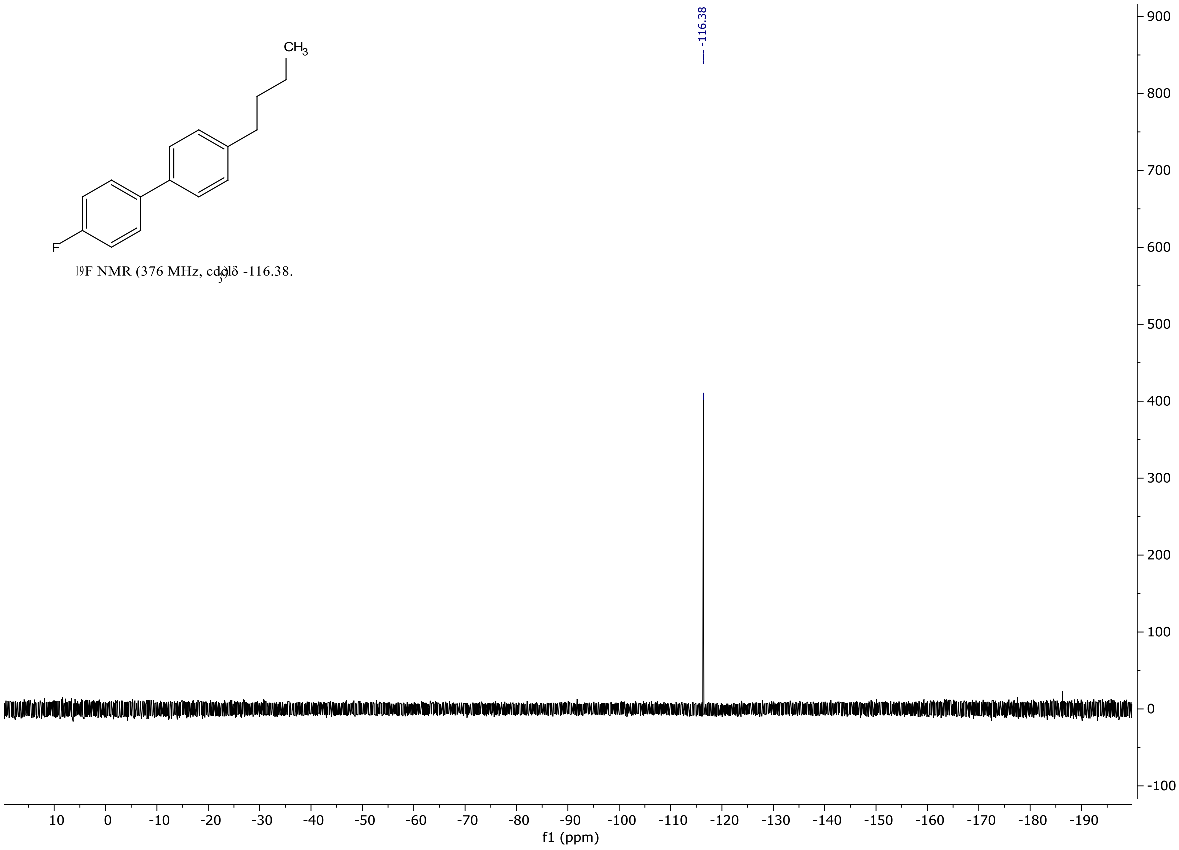

19F NMR (376 MHz, cdcl3) δ -116.38.

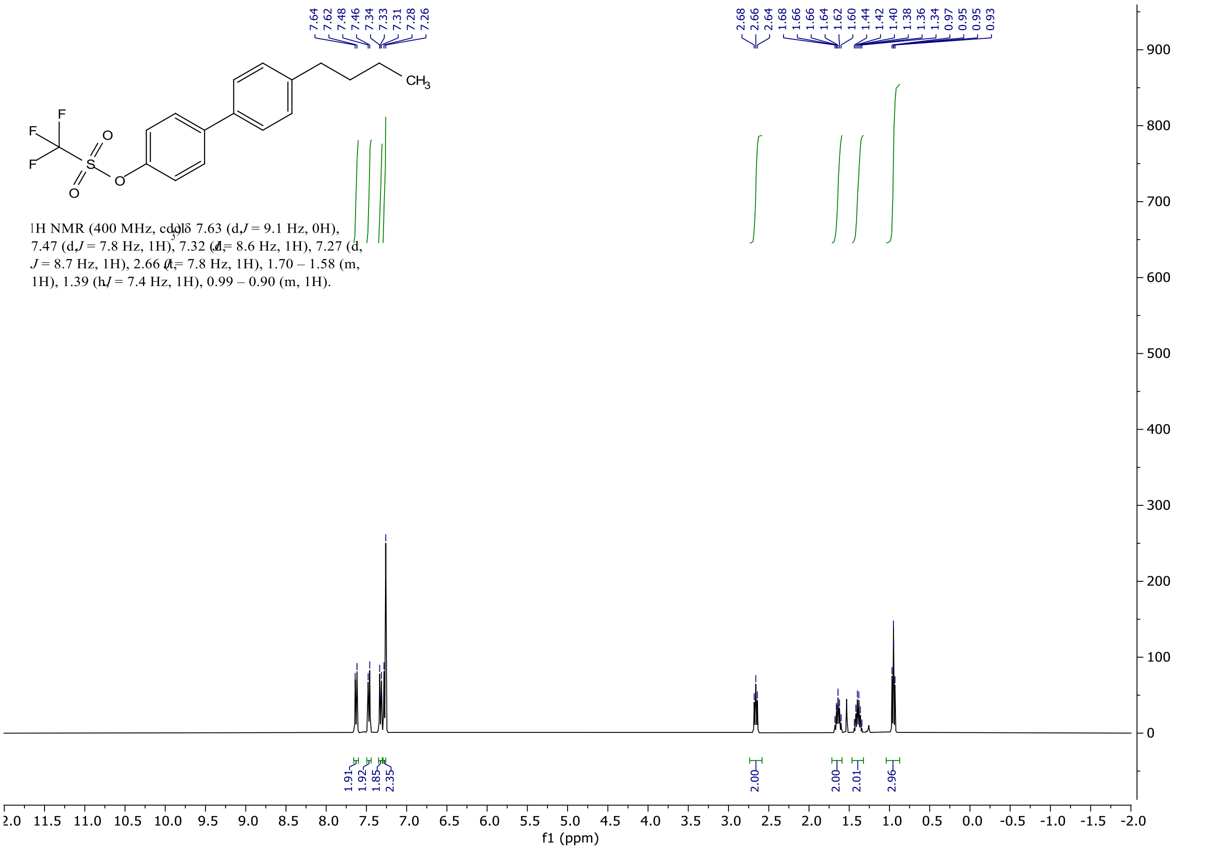
1H NMR (400 MHz, cdcl3) δ 7.63 (d, J = 9.1 Hz, 0H), 7.47 (d, J = 7.8 Hz, 1H), 7.32 (d, J = 8.6 Hz, 1H), 7.27 (d, J = 8.7 Hz, 1H), 2.66 (t, J = 7.8 Hz, 1H), 1.70 – 1.58 (m, 1H), 1.39 (h, J = 7.4 Hz, 1H), 0.99 – 0.90 (m, 1H).
f1 (ppm)

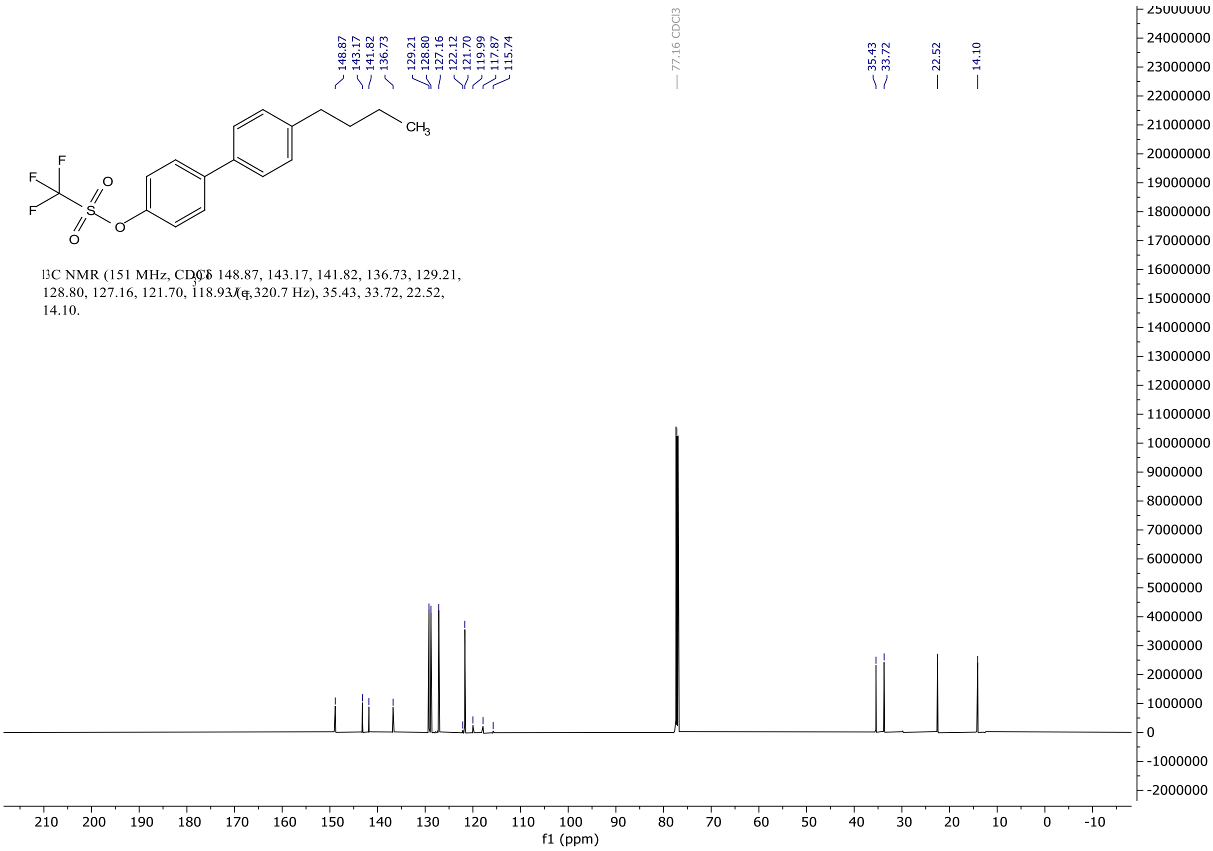
13C NMR (151 MHz, CDCl3) δ 148.87, 143.17, 141.82, 136.73, 129.21, 128.80, 127.16, 121.70, 118.93 (q, J = 320.7 Hz), 35.43, 33.72, 22.52, 14.10.
f1 (ppm)

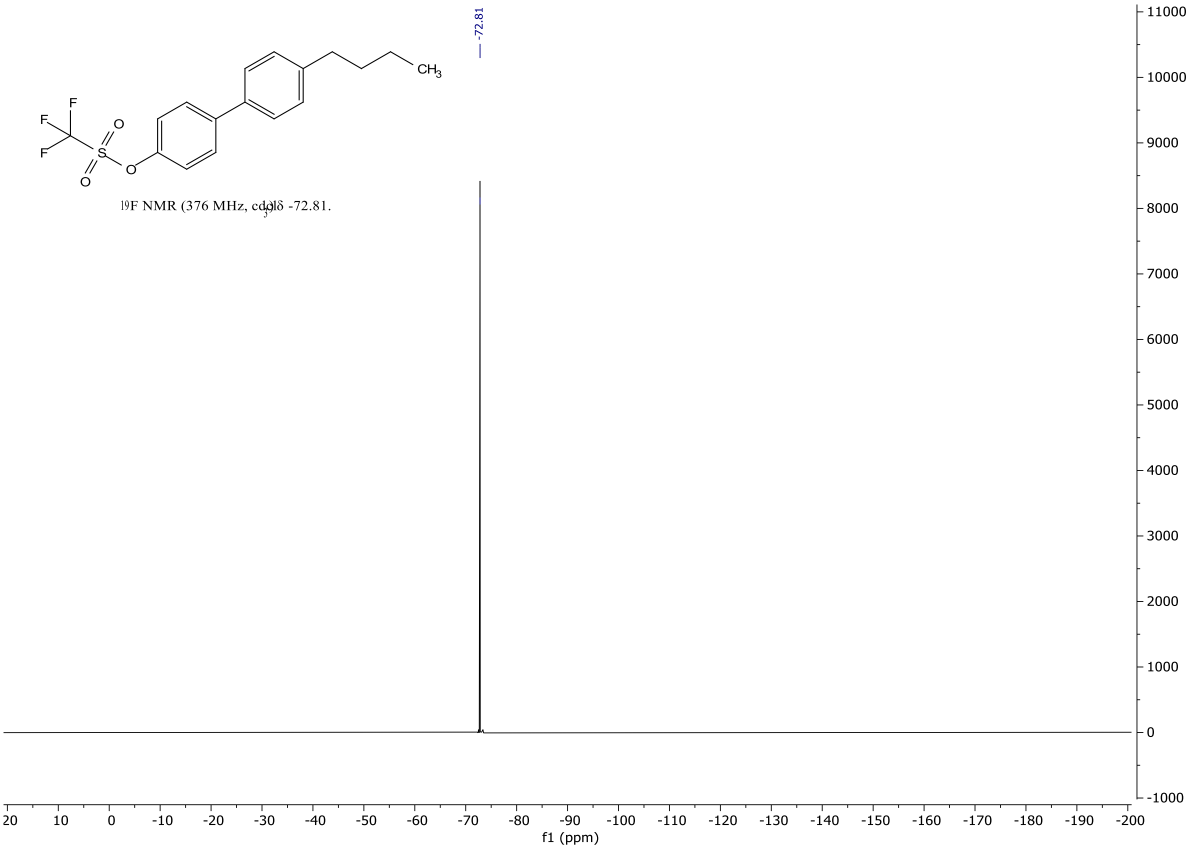

-72.81
CH3
F
F
F
O
S
O
O
19F NMR (376 MHz, cdcl3) δ -72.81.
11000
10000
9000
8000
7000
6000
5000
4000
3000
2000
1000
0
-1000
20 10 0 -10 -20 -30 -40 -50 -60 -70 -80 -90 -100 -110 -120 -130 -140 -150 -160 -170 -180 -190 -200
f1 (ppm)

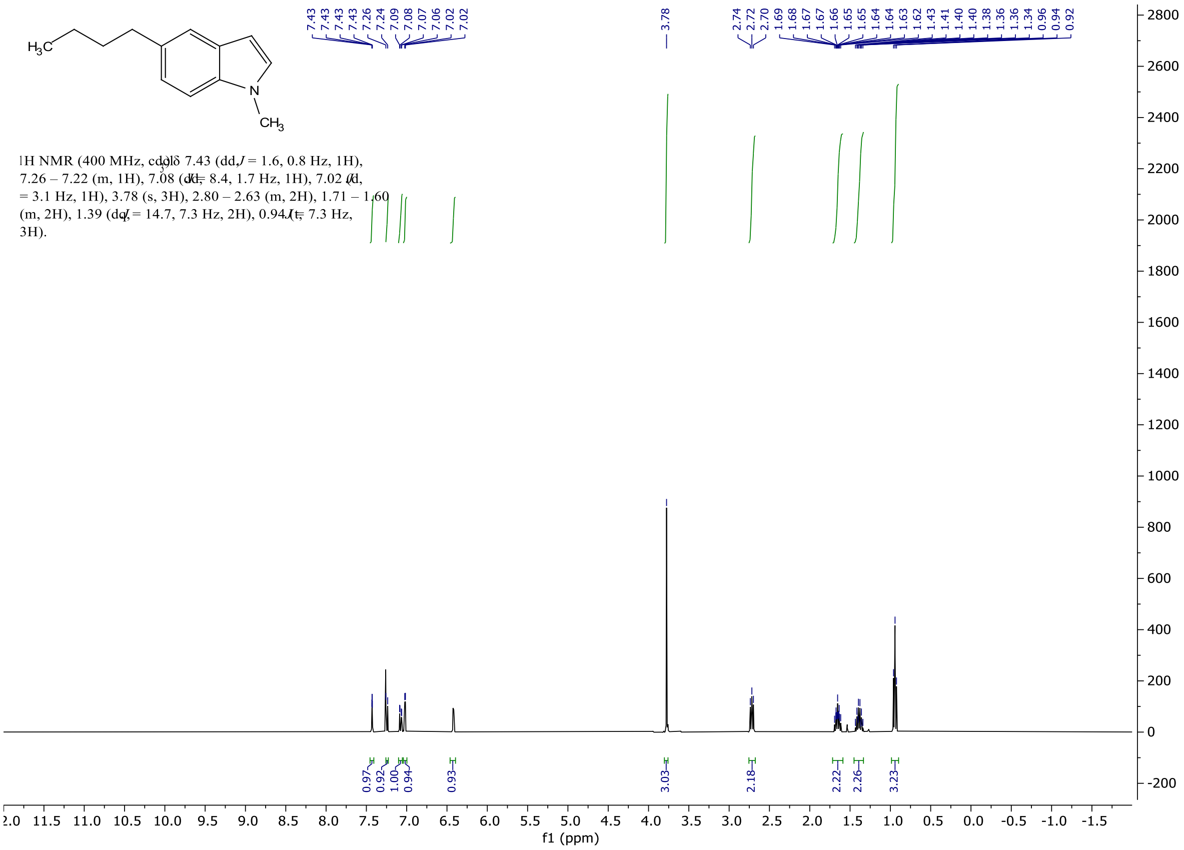
H3C
N
CH3
1H NMR (400 MHz, cdcl3) δ 7.43 (dd, J = 1.6, 0.8 Hz, 1H), 7.26 – 7.22 (m, 1H), 7.08 (dd, J = 8.4, 1.7 Hz, 1H), 7.02 (d, J = 3.1 Hz, 1H), 3.78 (s, 3H), 2.80 – 2.63 (m, 2H), 1.71 – 1.60 (m, 2H), 1.39 (dq, J = 14.7, 7.3 Hz, 2H), 0.94 (t, J = 7.3 Hz, 3H).
f1 (ppm)

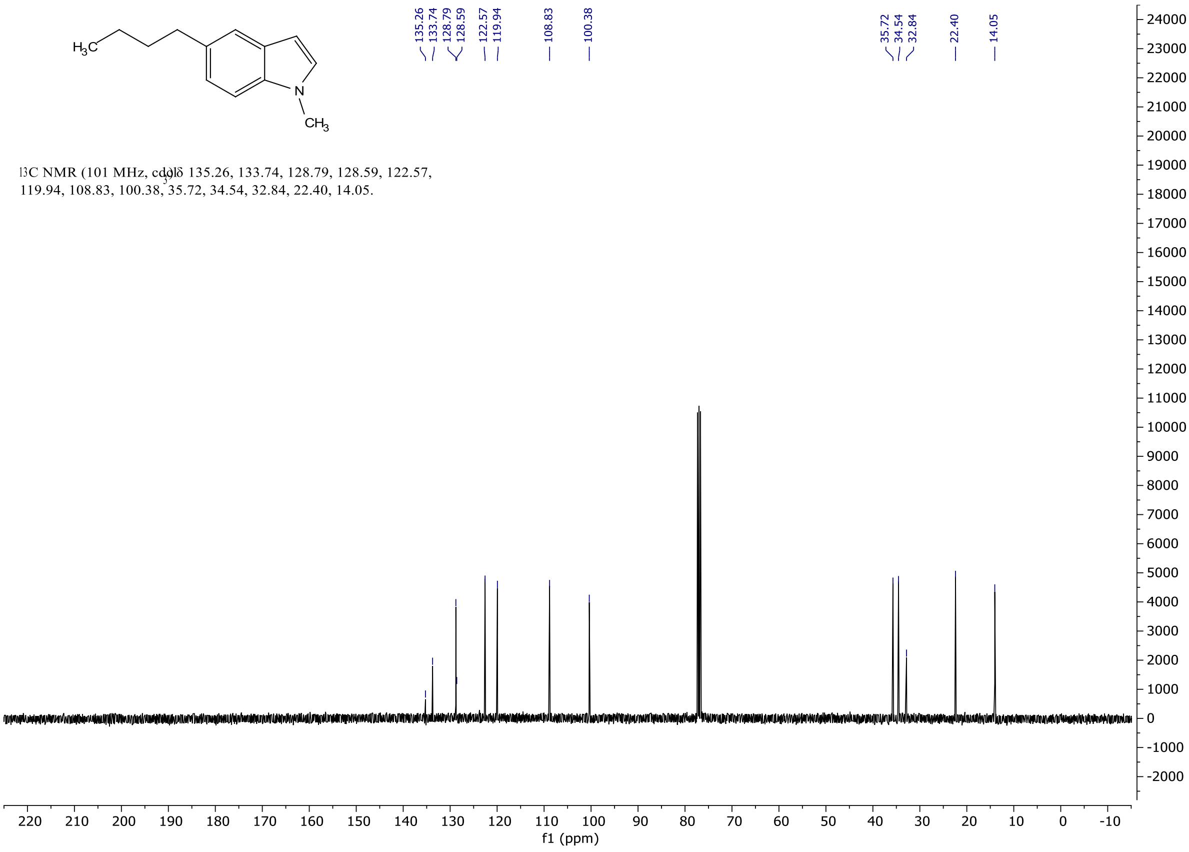
H3C
N
CH3
13C NMR (101 MHz, cdcl3) δ 135.26, 133.74, 128.79, 128.59, 122.57, 119.94, 108.83, 100.38, 35.72, 34.54, 32.84, 22.40, 14.05.
f1 (ppm)

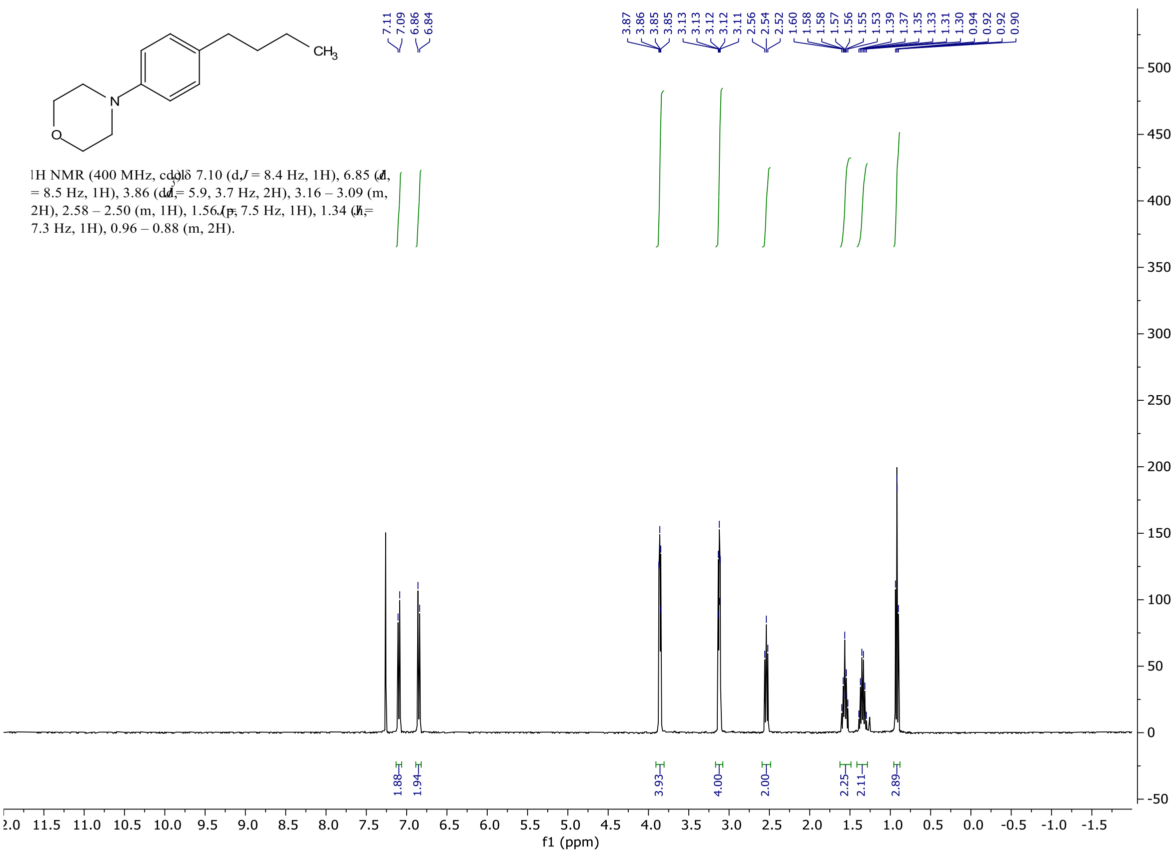

1H NMR (400 MHz, cdcl$_3$) δ 7.10 (d, J = 8.4 Hz, 1H), 6.85 (d, J = 8.5 Hz, 1H), 3.86 (dd, J = 5.9, 3.7 Hz, 2H), 3.16 – 3.09 (m, 2H), 2.58 – 2.50 (m, 1H), 1.56 (p, J = 7.5 Hz, 1H), 1.34 (h, J = 7.3 Hz, 1H), 0.96 – 0.88 (m, 2H).

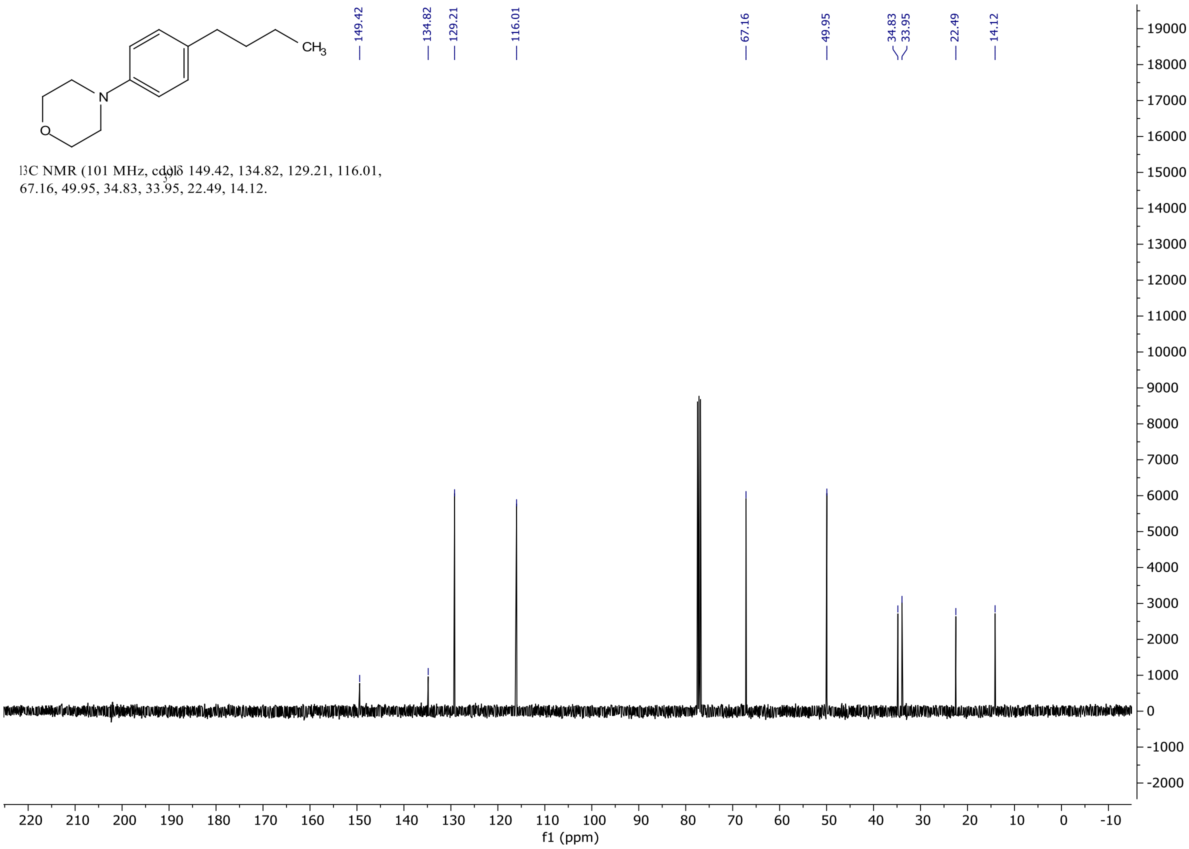

13C NMR (101 MHz, cdcl$_3$) δ 149.42, 134.82, 129.21, 116.01, 67.16, 49.95, 34.83, 33.95, 22.49, 14.12.

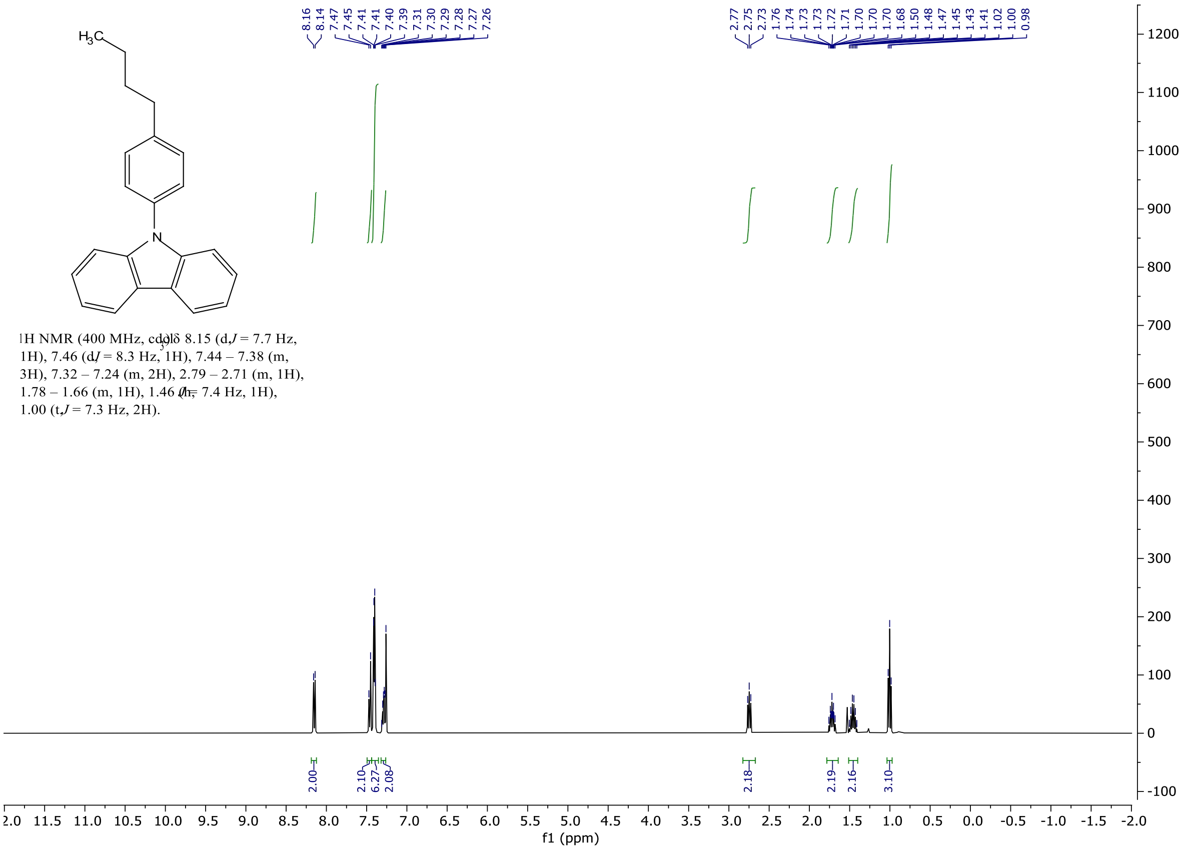

1H NMR (400 MHz, cdcl3) δ 8.15 (d, J = 7.7 Hz, 1H), 7.46 (d, J = 8.3 Hz, 1H), 7.44 – 7.38 (m, 3H), 7.32 – 7.24 (m, 2H), 2.79 – 2.71 (m, 1H), 1.78 – 1.66 (m, 1H), 1.46 (h, J = 7.4 Hz, 1H), 1.00 (t, J = 7.3 Hz, 2H).

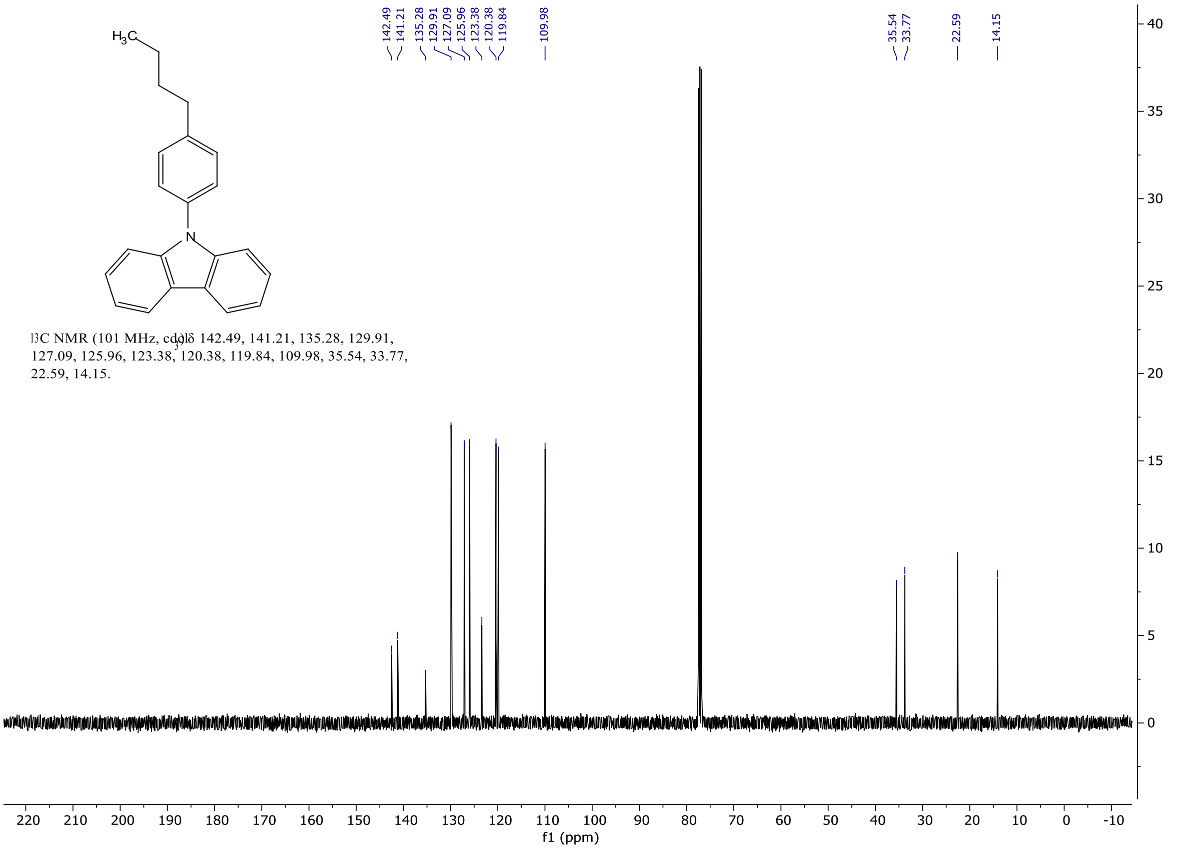

13C NMR (101 MHz, cdcl3) δ 142.49, 141.21, 135.28, 129.91, 127.09, 125.96, 123.38, 120.38, 119.84, 109.98, 35.54, 33.77, 22.59, 14.15.

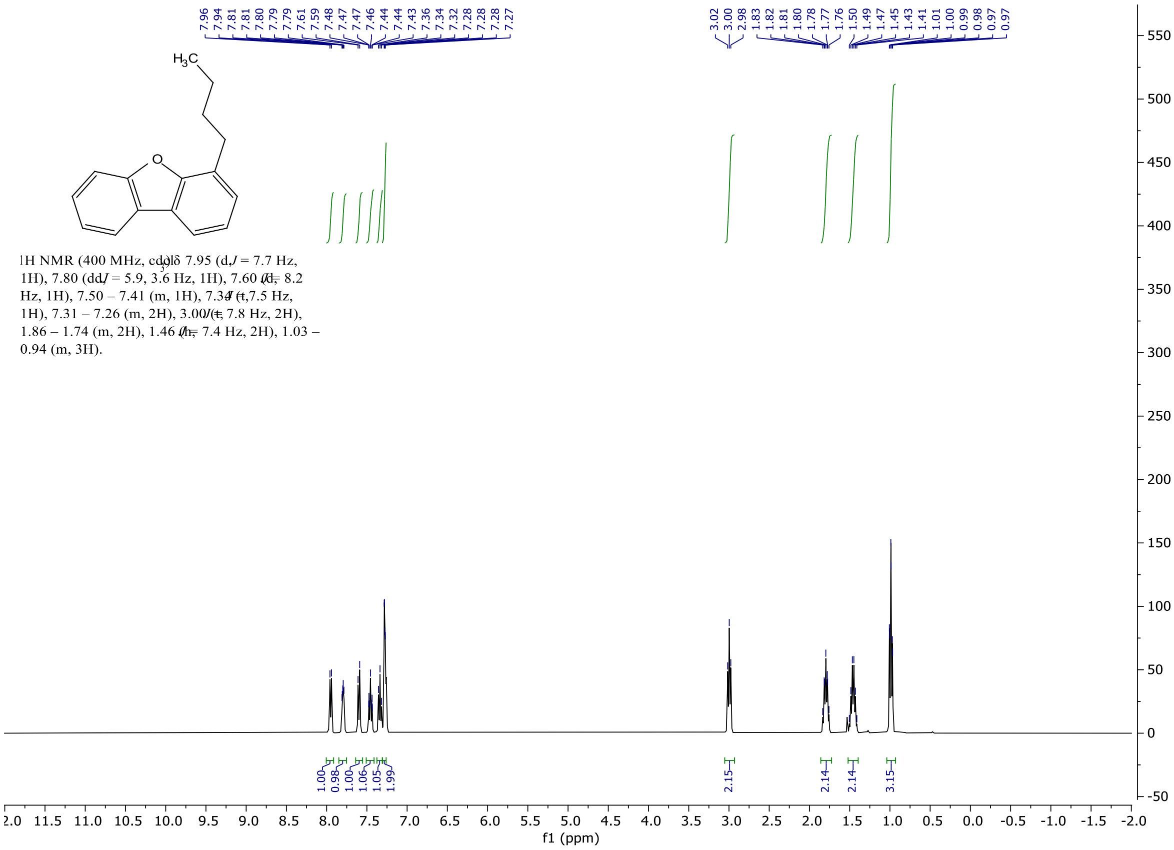

1H NMR (400 MHz, $cdcl_3$) δ 7.95 (d, J = 7.7 Hz, 1H), 7.80 (dd, J = 5.9, 3.6 Hz, 1H), 7.60 (d, J = 8.2 Hz, 1H), 7.50 – 7.41 (m, 1H), 7.34 (t, J = 7.5 Hz, 1H), 7.31 – 7.26 (m, 2H), 3.00 (t, J = 7.8 Hz, 2H), 1.86 – 1.74 (m, 2H), 1.46 (h, J = 7.4 Hz, 2H), 1.03 – 0.94 (m, 3H).

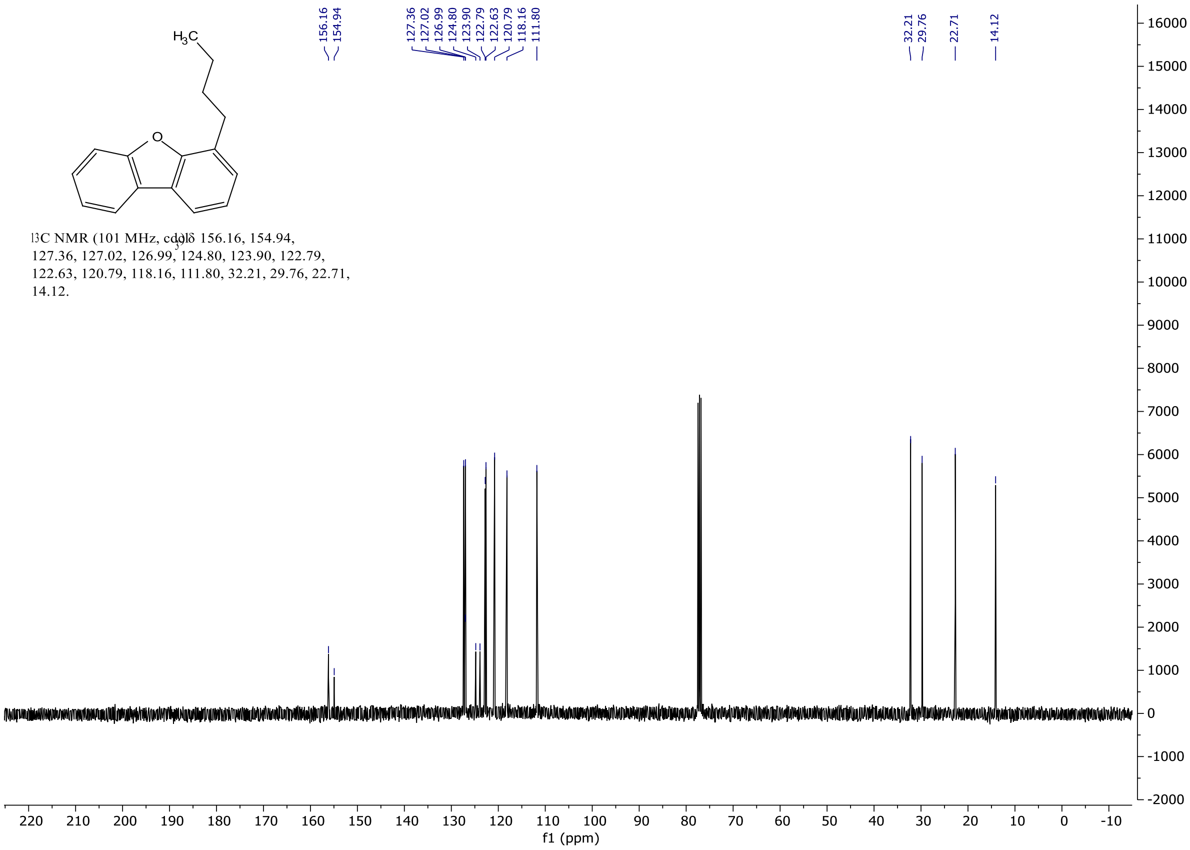

13C NMR (101 MHz, $cdcl_3$) δ 156.16, 154.94, 127.36, 127.02, 126.99, 124.80, 123.90, 122.79, 122.63, 120.79, 118.16, 111.80, 32.21, 29.76, 22.71, 14.12.

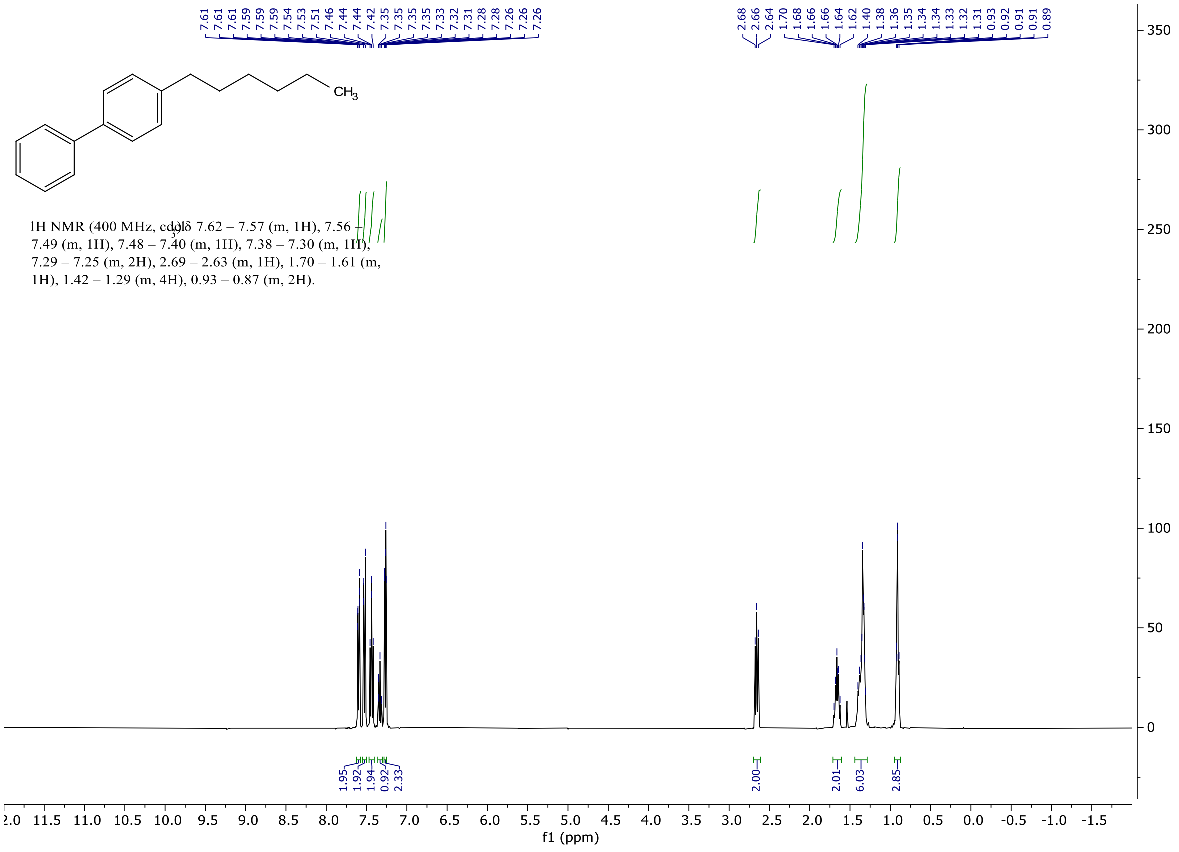
CH3
1H NMR (400 MHz, cdcl3) δ 7.62 – 7.57 (m, 1H), 7.56 – 7.49 (m, 1H), 7.48 – 7.40 (m, 1H), 7.38 – 7.30 (m, 1H), 7.29 – 7.25 (m, 2H), 2.69 – 2.63 (m, 1H), 1.70 – 1.61 (m, 1H), 1.42 – 1.29 (m, 4H), 0.93 – 0.87 (m, 2H).
f1 (ppm)

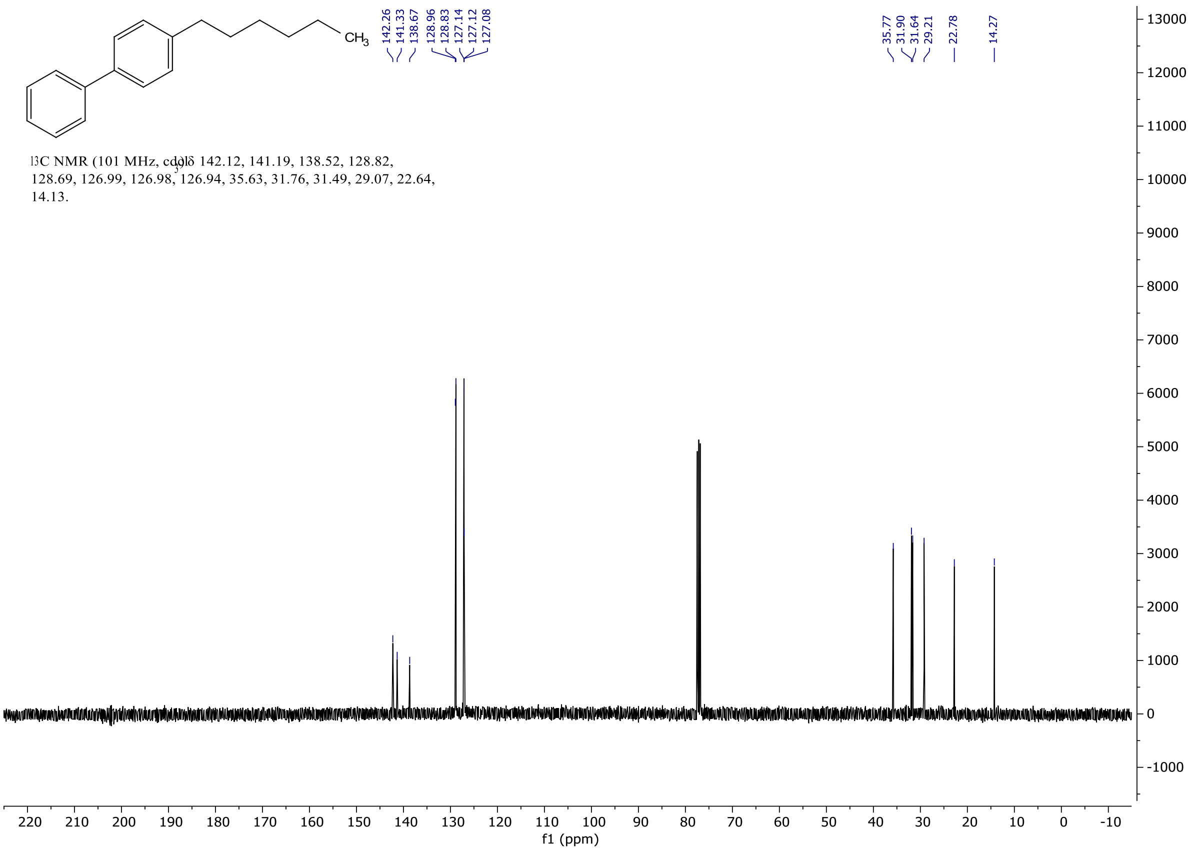
CH3
13C NMR (101 MHz, cdcl3) δ 142.12, 141.19, 138.52, 128.82, 128.69, 126.99, 126.98, 126.94, 35.63, 31.76, 31.49, 29.07, 22.64, 14.13.
142.26
141.33
138.67
128.96
128.83
127.14
127.12
127.08
35.77
31.90
31.64
29.21
22.78
14.27
f1 (ppm)

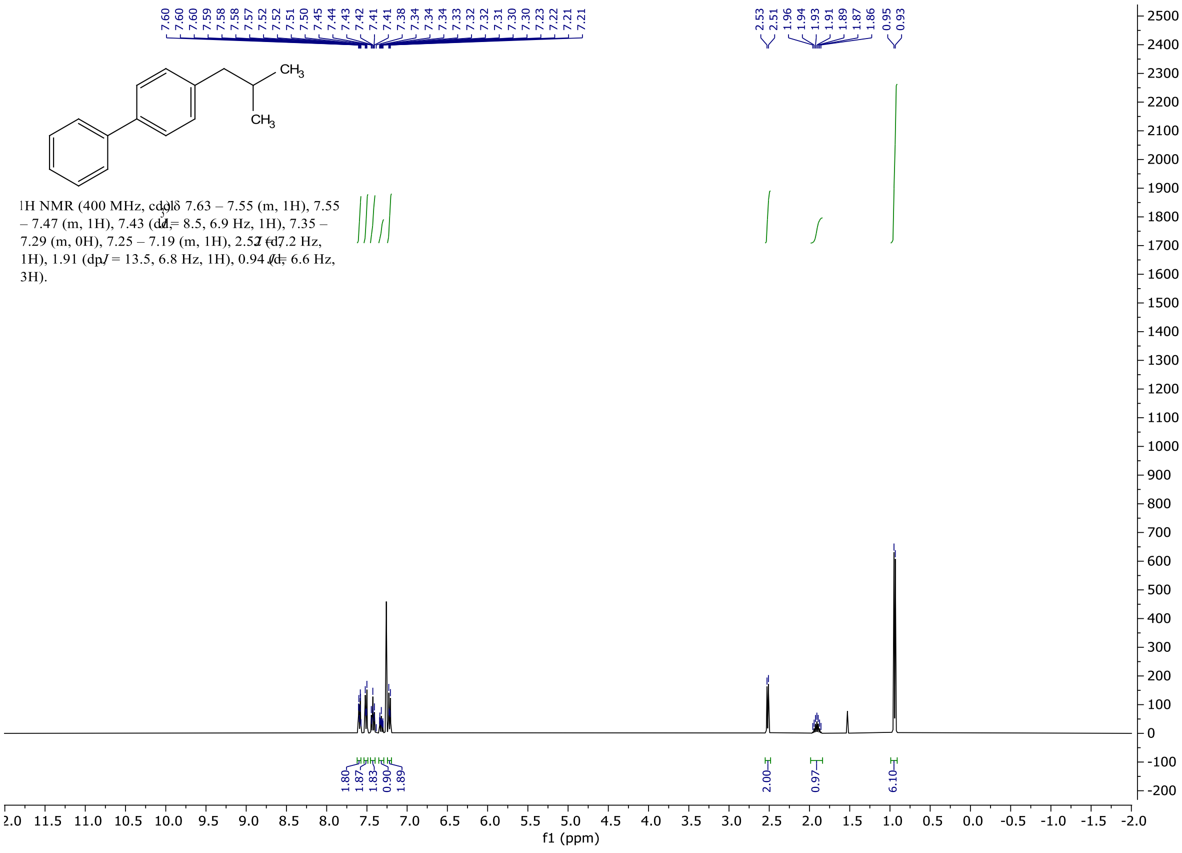
CH3
CH3
1H NMR (400 MHz, cdcl3) δ 7.63 – 7.55 (m, 1H), 7.55 – 7.47 (m, 1H), 7.43 (dd, J = 8.5, 6.9 Hz, 1H), 7.35 – 7.29 (m, 0H), 7.25 – 7.19 (m, 1H), 2.52 (d, J = 7.2 Hz, 1H), 1.91 (dp, J = 13.5, 6.8 Hz, 1H), 0.94 (d, J = 6.6 Hz, 3H).
f1 (ppm)

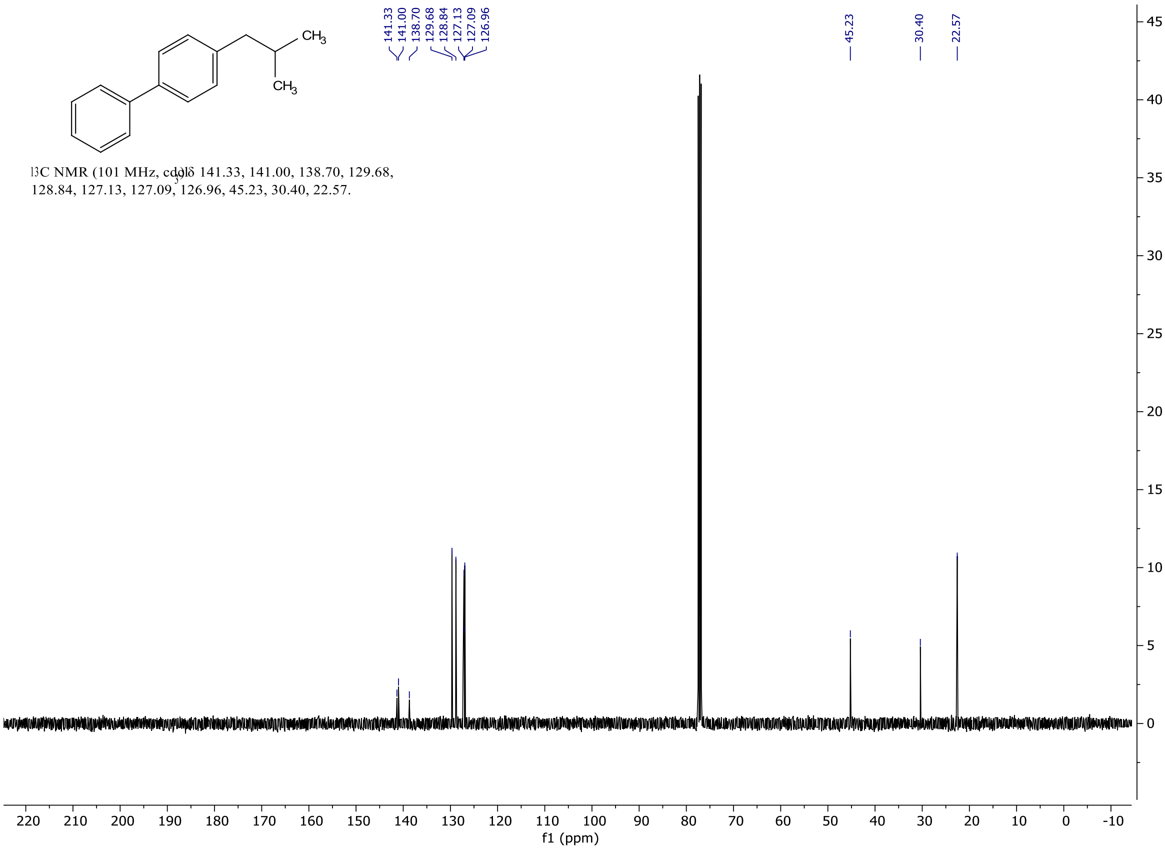
CH3
CH3
13C NMR (101 MHz, cdcl3) δ 141.33, 141.00, 138.70, 129.68, 128.84, 127.13, 127.09, 126.96, 45.23, 30.40, 22.57.
f1 (ppm)

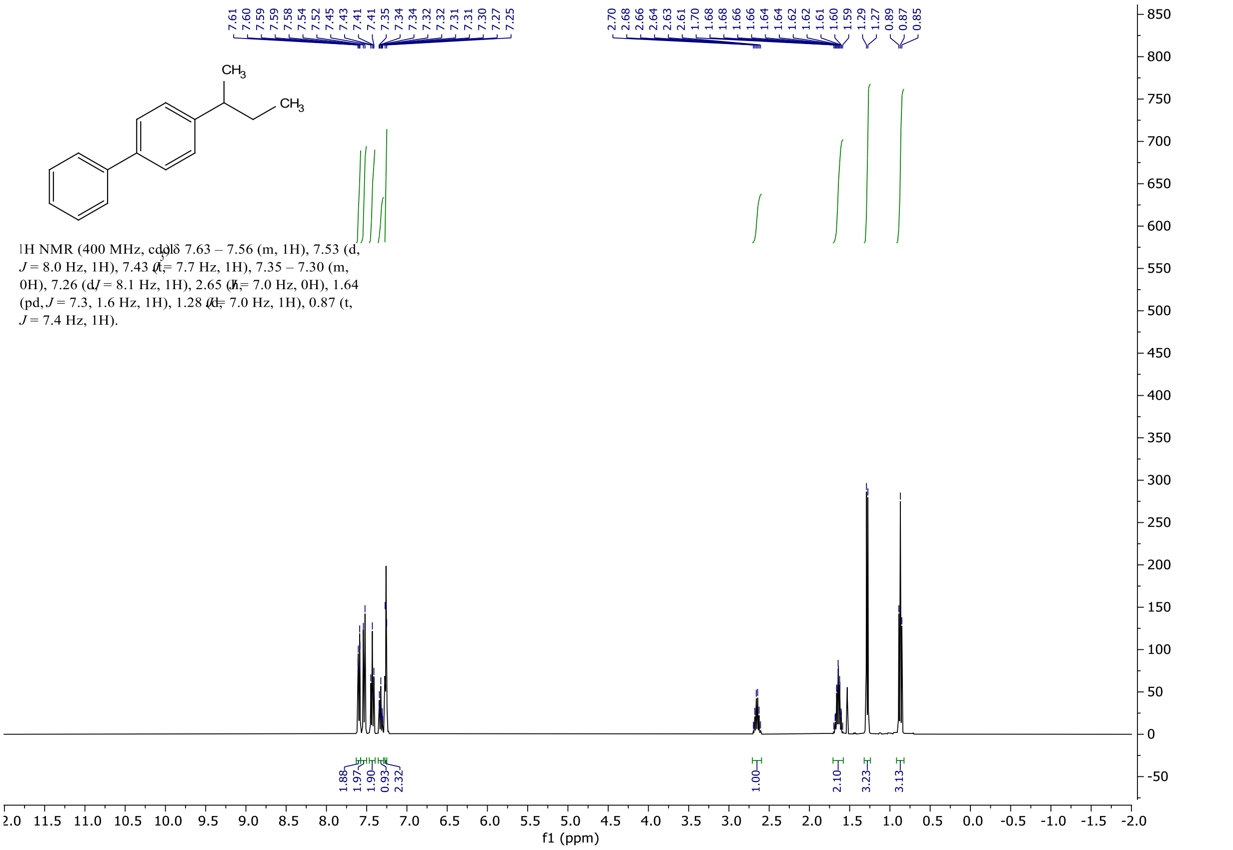

^{1}H NMR (400 MHz, cdcl3) δ 7.63 – 7.56 (m, 1H), 7.53 (d, *J* = 8.0 Hz, 1H), 7.43 (t, *J* = 7.7 Hz, 1H), 7.35 – 7.30 (m, 0H), 7.26 (d, *J* = 8.1 Hz, 1H), 2.65 (h, *J* = 7.0 Hz, 0H), 1.64 (pd, *J* = 7.3, 1.6 Hz, 1H), 1.28 (d, *J* = 7.0 Hz, 1H), 0.87 (t, *J* = 7.4 Hz, 1H).

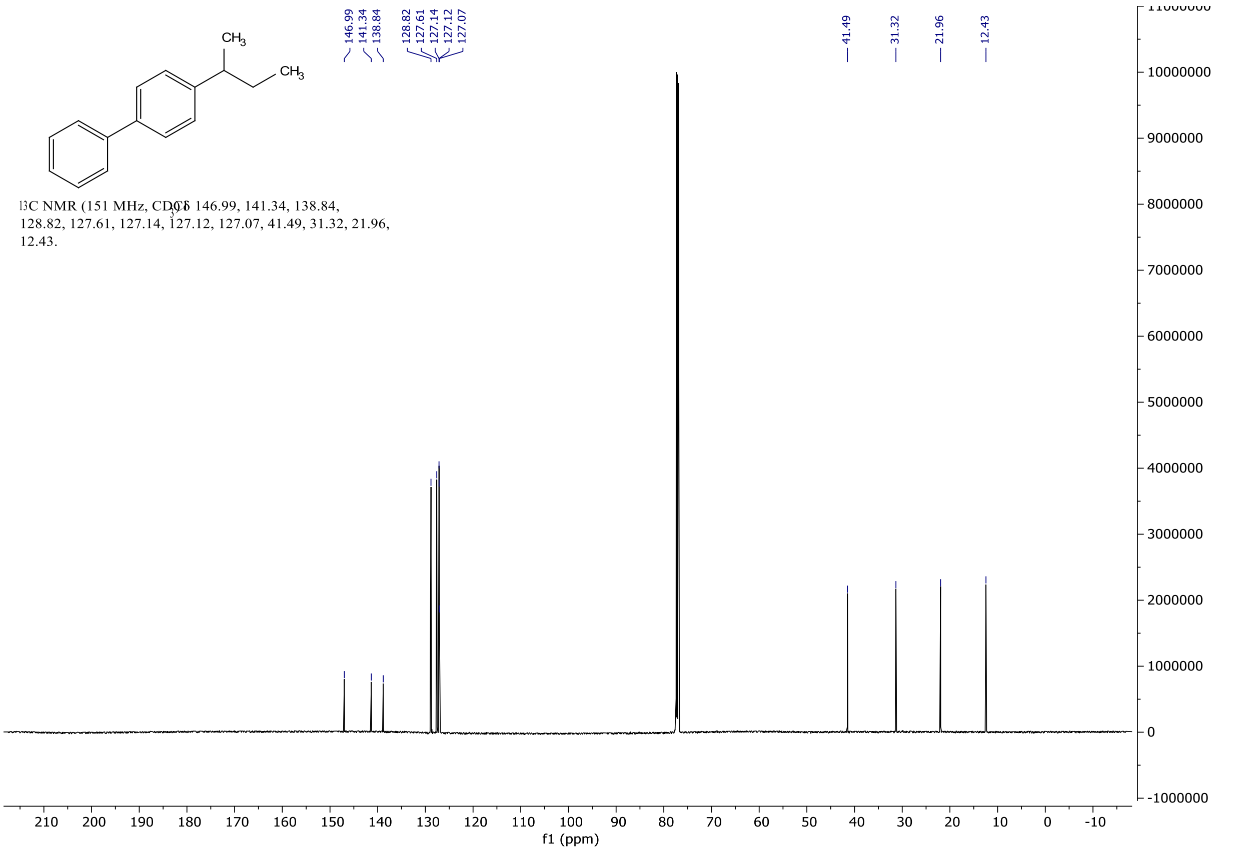

^{13}C NMR (151 MHz, $CDCl_3$) δ 146.99, 141.34, 138.84, 128.82, 127.61, 127.14, 127.12, 127.07, 41.49, 31.32, 21.96, 12.43.

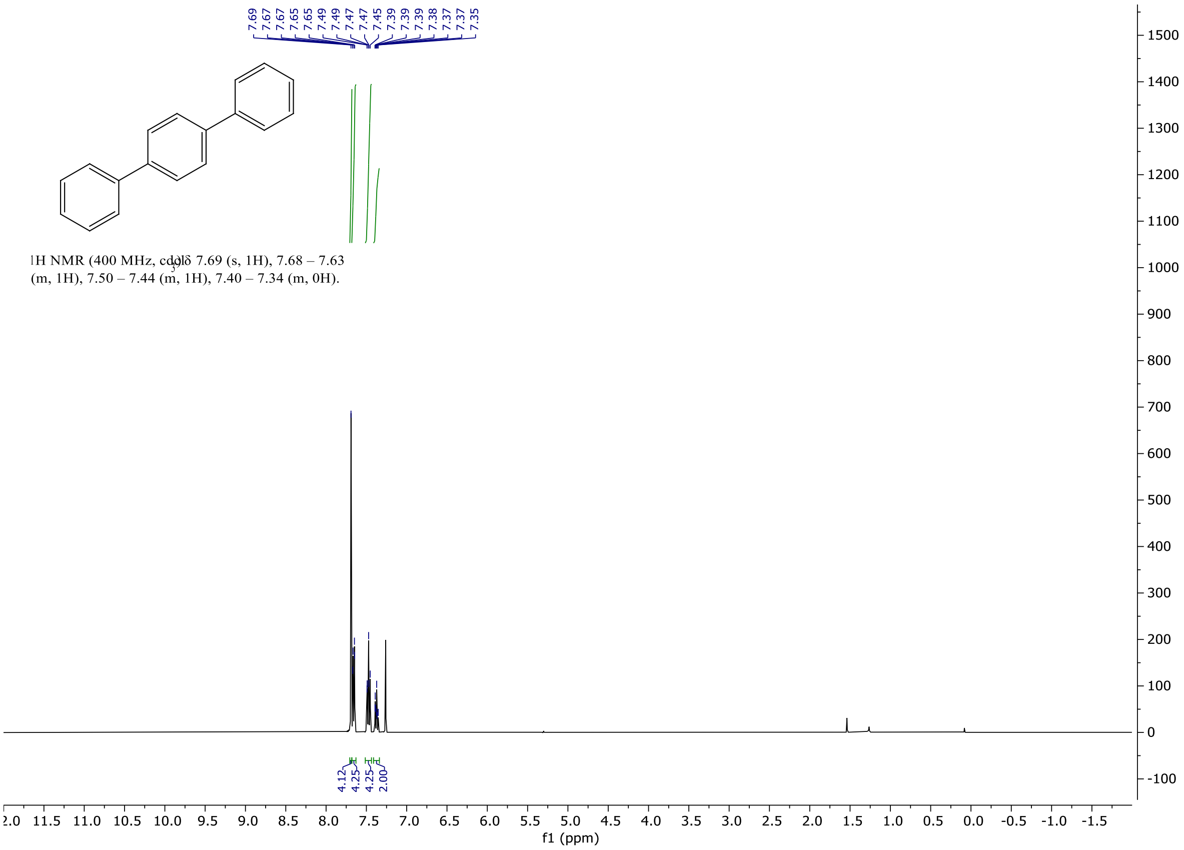
7.69 7.67 7.67 7.65 7.65 7.49 7.49 7.47 7.47 7.45 7.39 7.39 7.39 7.38 7.37 7.37 7.35
1H NMR (400 MHz, cdcl3) δ 7.69 (s, 1H), 7.68 – 7.63 (m, 1H), 7.50 – 7.44 (m, 1H), 7.40 – 7.34 (m, 0H).
4.12 4.25 4.25 2.00
f1 (ppm)

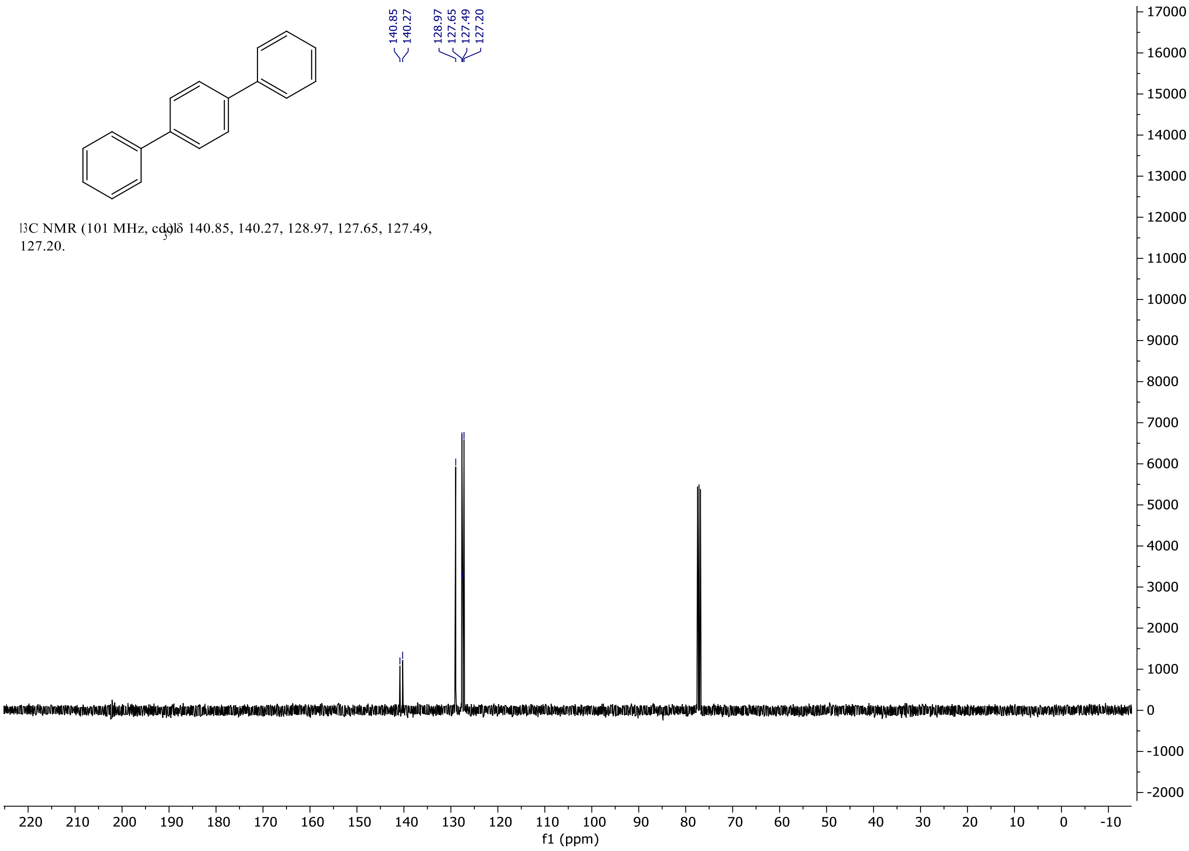
140.85 140.27 128.97 127.65 127.49 127.20
13C NMR (101 MHz, cdcl3) δ 140.85, 140.27, 128.97, 127.65, 127.49, 127.20.
f1 (ppm)

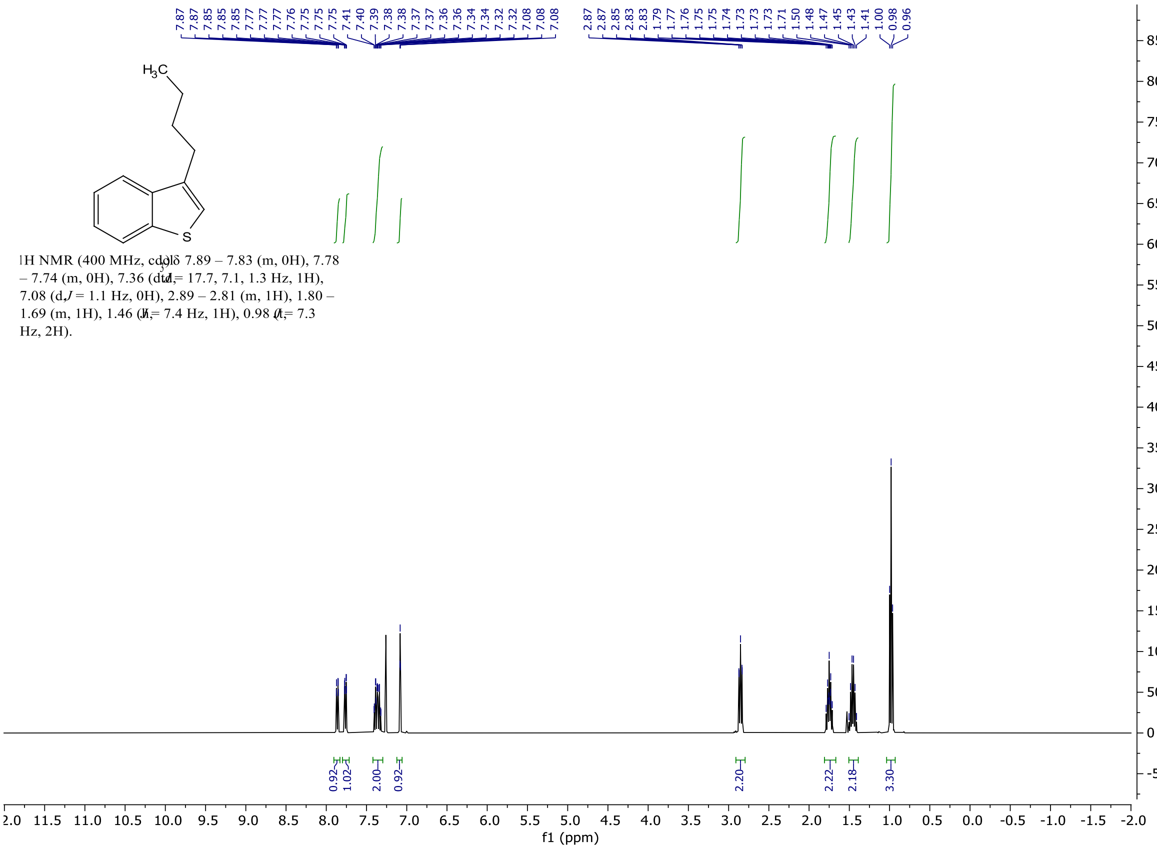

1H NMR (400 MHz, cdcl3) δ 7.89 – 7.83 (m, 0H), 7.78 – 7.74 (m, 0H), 7.36 (dtd, J = 17.7, 7.1, 1.3 Hz, 1H), 7.08 (d, J = 1.1 Hz, 0H), 2.89 – 2.81 (m, 1H), 1.80 – 1.69 (m, 1H), 1.46 (h, J = 7.4 Hz, 1H), 0.98 (t, J = 7.3 Hz, 2H).

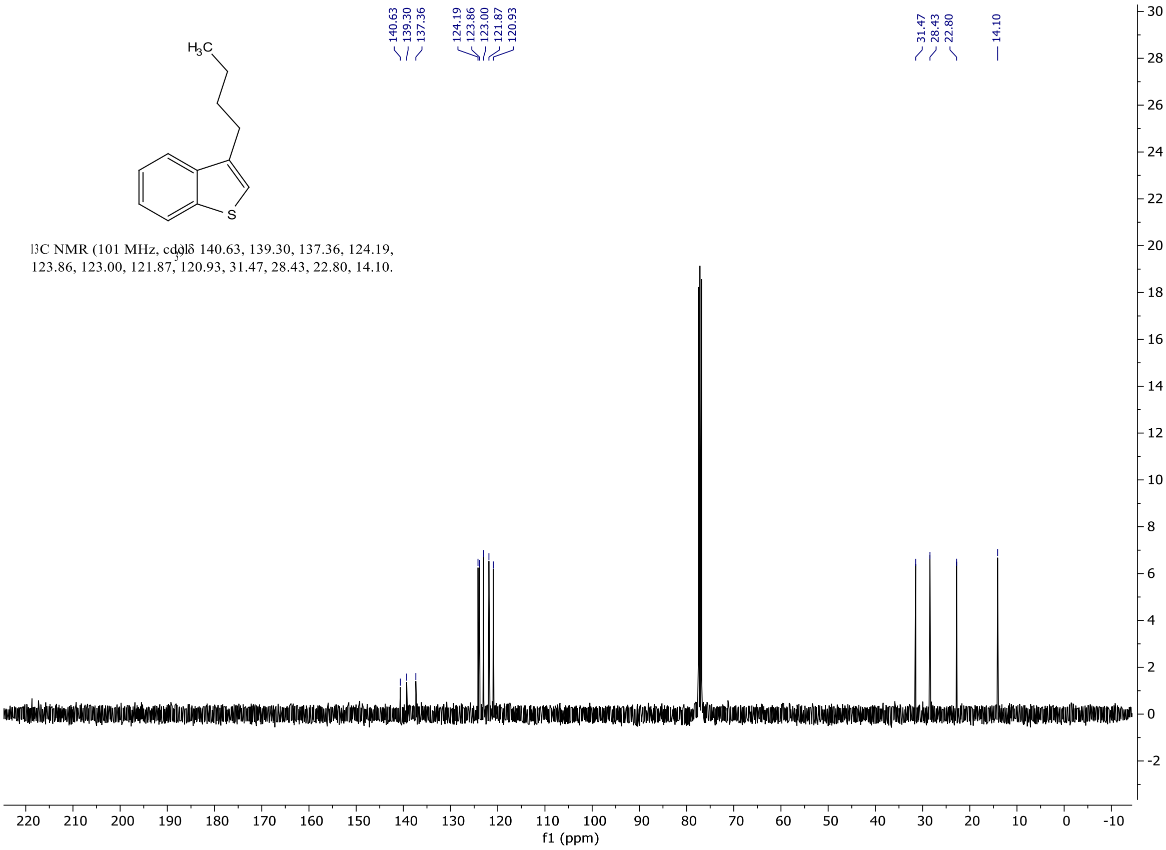

13C NMR (101 MHz, cdcl3) δ 140.63, 139.30, 137.36, 124.19, 123.86, 123.00, 121.87, 120.93, 31.47, 28.43, 22.80, 14.10.
